# Supported Ionic Liquid Phase (SILP) Allylic Alkylation of Amines in Continuous Flow

**DOI:** 10.1002/cctc.202300381

**Published:** 2023-05-11

**Authors:** Kristof Stagel, Ádám Márk Pálvölgyi, Clémence Delmas, Michael Schnürch, Katharina Bica‐Schröder

**Affiliations:** ^1^ Institute of Applied Synthetic Chemistry TU Wien Getreidemarkt 9/163 1060 Wien Austria

**Keywords:** ionic liquid, chiral catalyst, SILP, Tsuji-Trost reaction, continuous flow

## Abstract

We present the use of Pd‐complex‐containing supported ionic liquid phases (SILPs) as a novel approach for continuous‐flow allylic alkylation of *N*‐nucleophiles. This immobilization strategy gave simple access to air‐tolerating catalyst frameworks, providing rapid and convenient access to various achiral and chiral *N*‐allylation products. Under optimized conditions, the flow‐reaction could be maintained for 3.5 hours with constant product output; meanwhile, only a marginal 0.7 wt % of ionic liquid leaching and no detectable palladium‐complex leaching could be observed.

## Graphical abstract

We present a novel strategy for the **amination of various allylic acetate derivatives** in continuous mode. The developed SILP‐based method provides simple and rapid access to allylic amine derivatives, relies on mild conditions and environmentally benign solvents such as 2‐methyltetrahydrofuran and heptane.

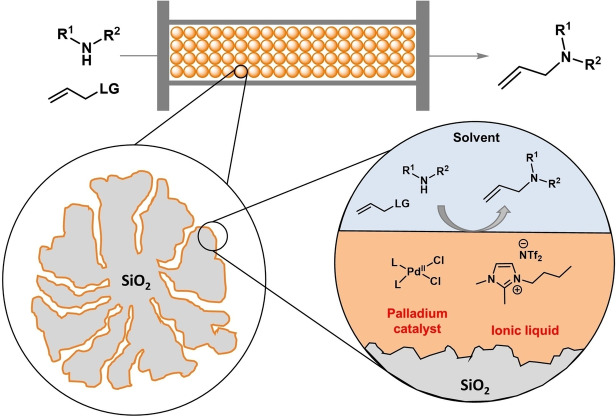

## Introduction

Transition metal‐catalyzed allylic alkylation reactions provide a versatile tool for various C−C and C−X bond formations and play an important role in synthesizing numerous biologically active compounds.^[1]^ Their use for synthesizing alkyl amines became particularly attractive: in contrast to classical alkylations with alkyl halides, such reactions require not just much milder reaction conditions but also results in significantly better product selectivity.^[2]^ In this field, the nucleophilic substitution of an activated allylic acetate or carbonate electrophile represents the most straightforward and widely investigated alkylation strategy. Apart from palladium‐catalyzed *Tsuji‐Trost*‐type reactions,^[3]^ other advances, including iridium^[4]^‐ or ruthenium‐catalysis,^[5]^ have also been reported.

The allylic alkylation of amines is mostly carried out *via* homogeneous catalysis and therefore hampered by the difficult catalyst separation, recycling, or reuse. However, most catalyst immobilization strategies require tedious multi‐step synthetic procedures, which might also lead to decreased catalytic efficiency. These issues can be effectively overcome with supported ionic liquid phase (SILP) catalysis:^[6]^ as such, a homogeneous catalyst is dissolved in an ionic liquid (IL), which is impregnated on a porous solid support. This concept efficiently combines the advantages of classical homogeneous and heterogeneous catalysis. Moreover, the thin impregnation of a highly porous surface with the liquid/catalyst also leads to significantly decreased ionic liquid and catalyst loadings compared to classical organic solvent/ionic liquid biphasic catalysis.^[7]^

Valkenberg and co‐workers summarized various methods for immobilizing ionic liquids.^[8]^ The synthesized Lewis‐acidic catalysts demonstrated good activities in Friedel‐Crafts alkylations. The concept of SILP catalysis is already applied on larger scales in gas‐ or supercritical phase reactions, including hydroformylations, hydrogenations, carbonylations,^[9]^ as well as continuous CO_2_ conversion.^[10]^ In terms of asymmetric catalysis, an approach from 2013 employs supercritical carbon dioxide as mobile phase in a continuous process for the asymmetric hydrogenation of dimethyl itaconate. The SILP catalyst system contains a chiral rhodium complex core and showed excellent reactivity and enantioselectivity in the asymmetric transformation.^[11]^ Additionally, Rufete‐Beneite *et al*. established a methodology that relies on the immobilization of a chiral complex, RhDuphos, with the aid of the SILP strategy. This catalyst system provided high conversions and with increased IL loadings, also excellent enantioselectivities in asymmetric hydrogenations.^[12]^

In comparison to reactions in gas or supercritical phase, the adaptation of the SILP concept to liquid‐phase reactions was found to be more challenging as the catalyst and/or the ionic liquid might be leached into the organic reaction media.^[13]^ However, some approaches successfully combine transition metal catalysis and SILPs in liquid‐phase reactions. In 2014, Urbán and his co‐workers reported the utilization of a SILP‐Pd catalyst framework in continuous Heck reactions, which demonstrated stable performance for several hours, providing high conversions.^[14]^ More *et al*. developed a Pd‐SILP‐system immobilized on Merrifield resin, which proved to be efficient and stable for Suzuki coupling reactions, and provided the corresponding products with excellent yields.^[15]^

**Figure 1:**
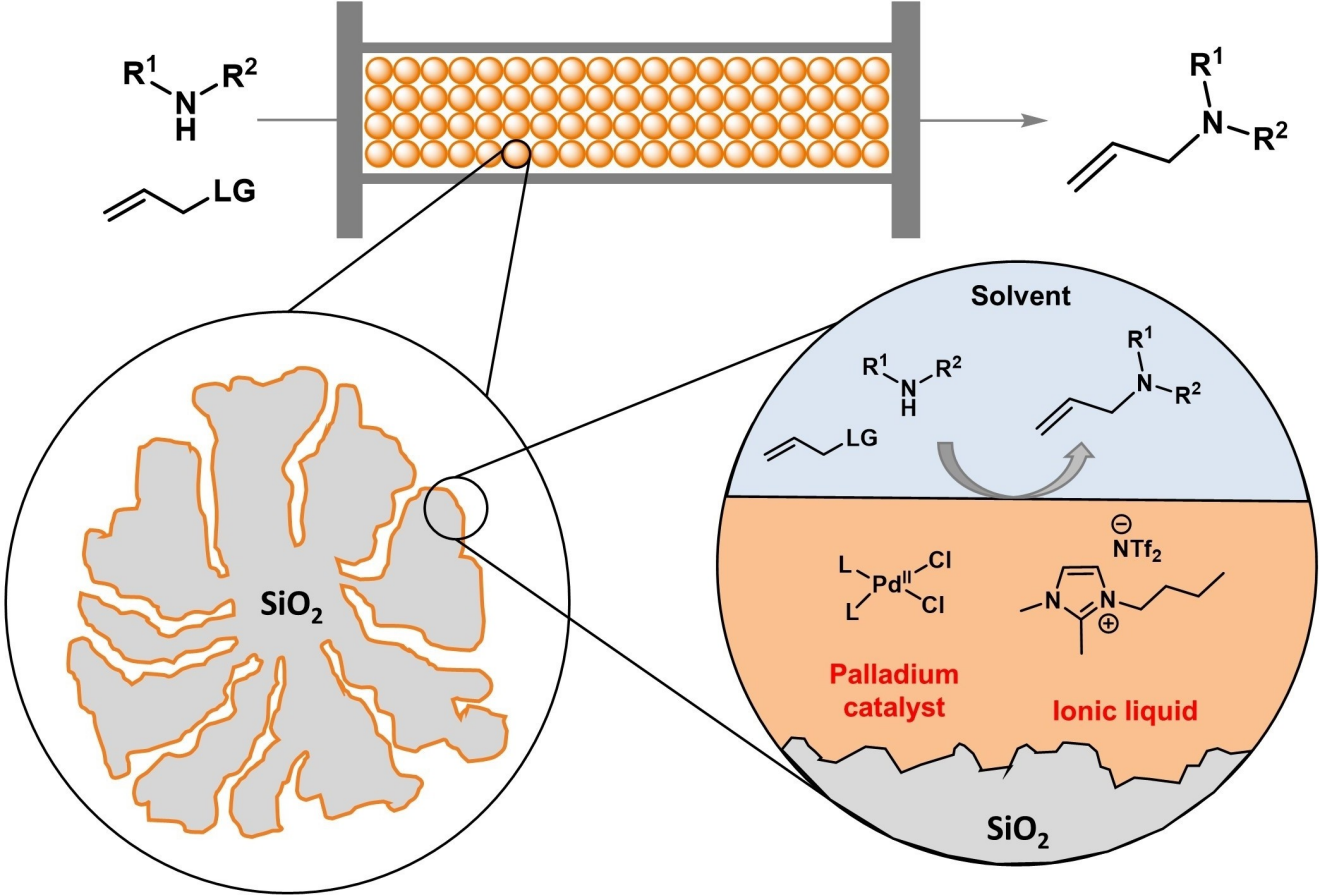
The concept of palladium‐based SILP catalysis for continuous‐flow allylation of *N*‐nucleophiles.

Based on our previous work on asymmetric allylations,^[16]^ we report herein the use of the SILP concept for the continuous‐flow allylic alkylation towards various achiral and chiral *N*‐allylation products. The simple physisorption of a palladium‐complex in hydrophobic ionic liquid onto silica surface provided straightforward access to air‐tolerant catalyst frameworks (Figure 1), which were successfully employed for allylic alkylation of *N*‐nucleophiles. This methodology requires neither an additional base nor an inert atmosphere and provides fast access to various achiral and chiral allylic amines.

## Results and Discussion

Based on our previous experiences with SILP‐catalysis for liquid‐phase reactions,[^13a^, ^17^] we aimed for a fast and simple preparation of the heterogeneous catalysts relying on hydrophobic ionic liquids. Because of the general insensitivity of palladium‐catalysts towards mildly acidic environment such as the OH groups of the silica gel,^[18]^ no deactivation or pre‐coating of the silanol groups was required. Using the cheap and readily available [Pd(C_3_H_5_)Cl]_2_ catalyst precursor, 1,3‐bis(diphenylphosphino)propane (dppp) and three different ionic liquds (**IL1**–**3**); a series of SILPs (**SILP 1**–**3**) with 10, 15, 20, and 30 wt % IL‐loading was prepared by simple physisorption of the IL/PdLn on the silica surface, respectively.

With these SILPs in hand, we initially tested their catalytic efficiency in the achiral continuous‐flow allylic substitution of cinnamyl acetate (**1 a**) with pyrrolidine (**2 a**) (Table 1). For this purpose, a continuous flow set‐up consisting of a cartridge employed as a fix‐bed reactor (0.707 mL) and a syringe pump was used (Supporting Information, Figure S5). A flow rate that corresponds to a residence time of 30 minutes was chosen. The crude product was directly analyzed to determine conversion *via* GC‐MS. The leaching of the ionic liquid was further quantified by ^19^F NMR spectroscopy with 2,2’‐difluorobenzophenone as internal standard, allowing a limit of detection in the order of >0.05 wt %.

**Table 1:** Parameter screening for the continuous‐flow synthesis of **3 a**.

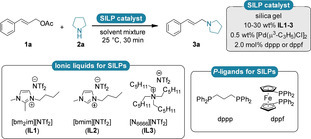			
Entry	Solvent	Ionic liquid	Conv. [%]^[b]^
1^[a]^	*n*‐heptane/2‐MeTHF 4/1	**IL1**	83
2^[c]^	*n*‐heptane/2‐MeTHF 4/1	**IL1**	73
3^[d]^	*n*‐heptane/2‐MeTHF 4/1	**IL1**	78
4	*n*‐heptane/2‐MeTHF 7/3	**IL1**	81
5^[e]^	toluene/2‐MeTHF 4/1	**IL1**	n. d.
6	*n*‐heptane/2‐MeTHF 4/1	**IL2**	77
7	*n*‐heptane/2‐MeTHF 4/1	**IL3**	69
8^[f]^	*n*‐heptane/2‐MeTHF 4/1	**IL1**	0
9^[g]^	*n*‐heptane/2‐MeTHF 4/1	**IL1**	83
10^[f]^	*n*‐heptane/2‐MeTHF 4/1	**IL2**	0

[a] Reaction was performed on a 0.6 mmol scale using cinnamyl acetate (**1 a**, 1.0 equiv.), pyrrolidine (**2 a**, 2.0 equiv.) with 450 mg **SILP1** (15 wt % **IL**, 0.5 wt % [Pd(C_3_H_5_)Cl]_2_ and 1.13 wt % dppp). The residence time was 30 minutes. [b] Determined by GC‐MS analysis. No side‐product was observed. [c] Performed with 30 wt % IL. [d] 45 min residence time. [e] Leaching of the ionic liquid was observed (3 wt % of total IL amount), determined by ^19^F NMR spectroscopy (limit of detection: >0.05 wt %). [f] No *P*‐ligand for SILP preparation. [g] dppf as *P*‐ligand for SILP preparation.

The choice of an appropriate solvent is a key issue for liquid‐phase reactions with supported catalysts. On the one hand, the polarity needs to be sufficiently high to dissolve starting materials and maintain homogenous conditions through the reaction. On the other hand, polarity needs to be balanced in order to avoid leaching of the ionic liquid – an aspect that would rather favor apolar conditions.^[19]^ Keeping environmental aspects in mind, we decided to use a mixture of *n*‐heptane with ether‐type solvents as polar modifier. When using 20 V/V % of 2‐MeTHF, – a bio‐based solvent – in *n*‐heptane with the **SILP1**, the allylation product 1‐cinnamylpyrrolidine (**3**) was formed in 83 % conversion within 30 minutes of residence time (Table 1, entry 1); meanwhile, **SILP1** with higher IL‐loading or a longer residence time did not improve the conversion (Table 1, entries 1 vs. 2–3). Using an increased amount of 2‐MeTHF led to a similar conversion (Table 1, entry 4), whereas using toluene as the apolar component resulted in leaching (Table 1, entry 5). Furthermore, a SILP with 1‐butyl‐2,3‐dimethyl‐imidazolium bis(trifluoromethylsulfonyl)imide ([bm_2_im][NTf_2_], **IL1**) was indeed beneficial, as the SILPs with [bmim][NTf_2_] (**IL2**) or with the quaternary ammonium‐based **IL3** both resulted in slightly lower conversions (Table 1, entries 6–7). Imidazolium‐based ILs are known to form carbenes, as their C2 atom can undergo deprotonation under basic conditions.^[20]^ Such a species can coordinate to the Pd‐atom, resulting in the decoordination of the original ligand, which can lead to slightly decreased activity. If the C2 atom is protected with an alkyl group, deprotonation can be prevented. This can explain the slightly decreased activity of the SILP with **IL2** compared to **IL1**. While a phosphine ligand was indeed found to be crucial (Table 1, entry 8); no difference between dppp and 1,1’‐bis(diphenylphosphino)ferrocene (dppf) was observed (Table 1, entries 1 vs. 9); leaving the cheaper dppp as a preferential option. To further investigate the possibility of a Pd‐NHC strategy, the catalytic effect of the **IL2**‐based SILP in the absence of a *P*‐ligand was also investigated; however, no conversion was observed (entry 8).

**Figure 2:**
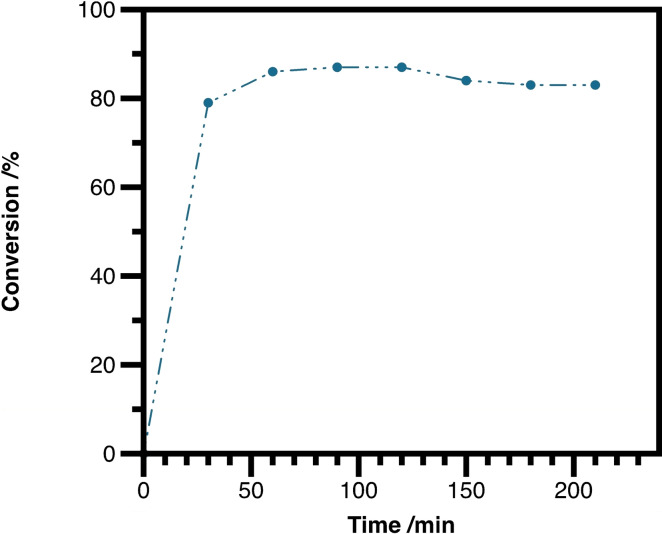
Long‐term stability of the developed SILP‐system. Reactions were carried out on a 3.2 mmol scale using 1.0 equiv. cinnamyl acetate (**1 a**) and 2.0 equiv. pyrrolidine (**2 a**) with 470 mg of **SILP1** (15 wt % **IL1**, 0.5 wt % [Pd(C_3_H_5_)Cl]_2_ and 1.13 wt % dppp) in *n*‐heptane/2‐MeTHF 4/1. The residence time was 30 minutes.

After identifying **SILP1** as the best‐performing catalyst and with improved reaction conditions in hand, we investigated the longer‐term usability of the SILP‐system. After equilibrating the reaction mixture for 30 minutes as previously optimized, the system was stabilized. A constant product output could be observed for a total of 3.5 hours, thus corresponding to seven reaction cycles (Figure 2) without any loss of catalytic activity. Furthermore, only a marginal 0.7 wt % ionic liquid leaching, and no palladium‐complex leaching could be observed.

**Scheme 1:**
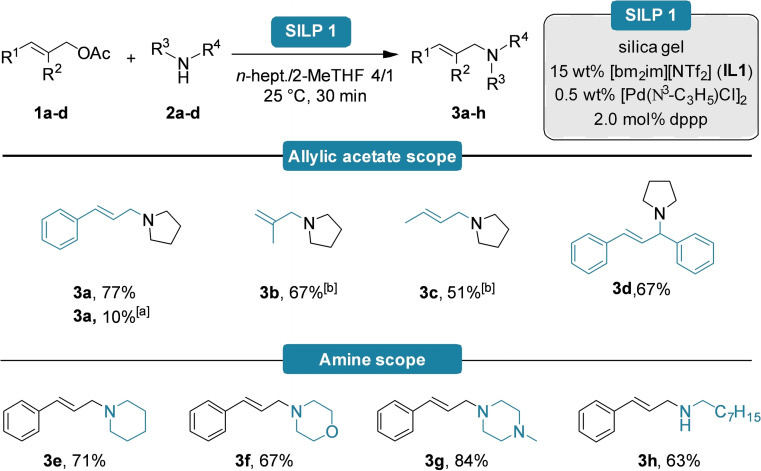
Substrate and reagent scope for the continuous‐flow allylic alkylation. Reactions were carried out on a 0.6 mmol scale using 1.0 equiv. allylic acetate (**1 a**–**d**) and 2.0 equiv. amine (**2 a**–**d**) with 450 mg of **SILP1** (15 wt % **IL1**, 0.5 wt % [Pd(C_3_H_5_)Cl]_2_ and 1.13 wt % dppp) in *n*‐heptane/2‐MeTHF 4/1. The residence time was 30 minutes. Yields refer to isolated products after column chromatographic purification. [a] Reaction was performed with cinnamyl alcohol, GC‐MS conversion. [b] GC‐MS conversion due to the extremely volatile nature.

We then explored the scope and limitations of the continuous‐flow *N*‐allylation reaction. A small set of allylic acetate electrophiles with different sterics were investigated, and the corresponding products **3 a**–**d** were obtained in good to excellent yields (Scheme 1). It is worth mentioning that the reaction with cinnamyl alcohol instead of cinnamyl acetate (**1 a**) still provided product **3 a** in 10 % yield, indicating that the silanol OH‐groups might have a moderate co‐catalytic effect on increasing the leaving group ability through H‐bonding. Despite this moderate and non‐optimized result, it could still be an important aspect, as the direct functionalization of allylic alcohols mostly requires harsh reaction conditions and/or strong Lewis or Brønsted acidic additives.^[21]^ Besides screening different allylic electrophiles, a few different *N*‐nucleophiles, including primary and secondary amines, have also been tested and the corresponding *N*‐allylated products (**3 e**–**h**) could be isolated in good to excellent yields (Scheme 1). Meanwhile, slightly higher yields could be achieved in the literature in batch mode; these methods mostly rely on exotic reagents and/or harsh reaction conditions.^[22]^ In comparison, this method uses common reagents, applies mild reaction conditions, and results in short reaction times in continuous‐flow operation.

Aiming to adapt the established SILP‐system to asymmetric allylic alkylations, we further envisioned expanding the system for the continuous‐flow asymmetric allylic alkylations of pyrrolidines. In general, palladium‐catalyzed enantioselective allylic alkylations have emerged as versatile and powerful chemical reactions for asymmetric carbon‐carbon and carbon‐heteroatom bond formation.^[23]^ Due to its numerous advantages, such as mild conditions, variable chiral ligands or nucleophiles, simple implementation, and good functional group tolerance; such allylations have found wide application in the synthesis of valuable optically active compounds. Since the seminal examples of Trost,^[24]^ a wide range of chiral ligands, including phosphinooxazolines,^[25]^ amino‐acid derivatives,^[26]^ amino‐phosphinites,^[27]^ phosphoramidites,^[28]^ and aminoalkyl‐phosphines^[29]^ have been developed for the asymmetric allylation of stabilized *C*‐nucleophiles. Despite the large number of advancements, up until today, the Trost‐type ligands still have maintained a privileged position among the chiral modifiers in the field of asymmetric allylations.^[30]^ Although Trost‐type ligands are well suited for unhindered substrates, they have proven less efficient for the alkylation of hindered substrates, such as *rac*‐1,3‐diphenylally acetate.^[30]^ Hitchcock's group^[31]^ circumvented these problems by introducing various ester groups to the ligand, using a series of *tert*‐leucinol‐derived diphosphines, which provided good yields in the allylation of the previously mentioned hindered substrate. In 2000, Kim *et al*.^[32]^ reported the synthesis of various *P*,*N*‐type monophosphine ligands with (*R*,*R*)‐diaminocyclohexane (*R*,*R*‐DACH) backbone, which showed good reactivities and moderate enantioselectivities in asymmetric allylic alkylation of more hindered substrates. With most of the recent advancements being indeed reported for C−C bond formation, the asymmetric allylations of *N*‐nucleophiles attracted significantly less attention.

In accordance with the findings for the non‐asymmetric *N*‐allylations, we initially screened different chiral SILPs (**chSILP**s) for the asymmetric *N*‐allylations of pyrrolidine (**2 a**) with (*rac*)‐1,3‐diphenylallyl acetate (**1 d**) both in heterogeneous batch slurry phase, as well as in continuous flow (Table 2). By using the same ionic liquid **IL1** and palladium‐precursor [Pd(C_3_H_5_)Cl]_2_ as before, a small set of **chSILP** catalysts were prepared using different chiral ligands (**L1**–**4**), and their catalytic efficiency was investigated in the same solvent mixture as for the non‐asymmetric *N*‐allylations (*n*‐heptane/2‐MeTHF 4/1). The **chSILP** with the traditional Trost‐ligand **L1** and bis(oxazoline) **L4** provided comparable reactivities and ee in slurry‐phase, whereas **L4** proved inactive in the continuous‐flow process (Table 2, entry 4). **L4** – as the only non‐Trost‐type ligand‐ differs from the other employed ligands in its structure, as it does not contain a strongly coordinating *P*‐atom. This might result in the ligand's different coordination properties and its loss in the continuous process due to leaching. On the other hand, the allylation product **3 d** could be obtained in continuous flow with moderate conversion and enantioselectivity when using the more sterically demanding Trost‐type ligand **L2**. The introduction of a *tert*‐butyl‐carbamate‐modified Trost‐ligand **L3** led to a further improved conversion and to good ee in flow; yielding the product **3 d** in a promising 81 % conversion and 74 % ee within 30 minutes of residence time.

**Table 2:** Screening of different chiral ligands for the continuous‐flow asymmetric *N*‐allylation of pyrrolidine (**2 a**) with **1 d** using **chSILP** catalysts.

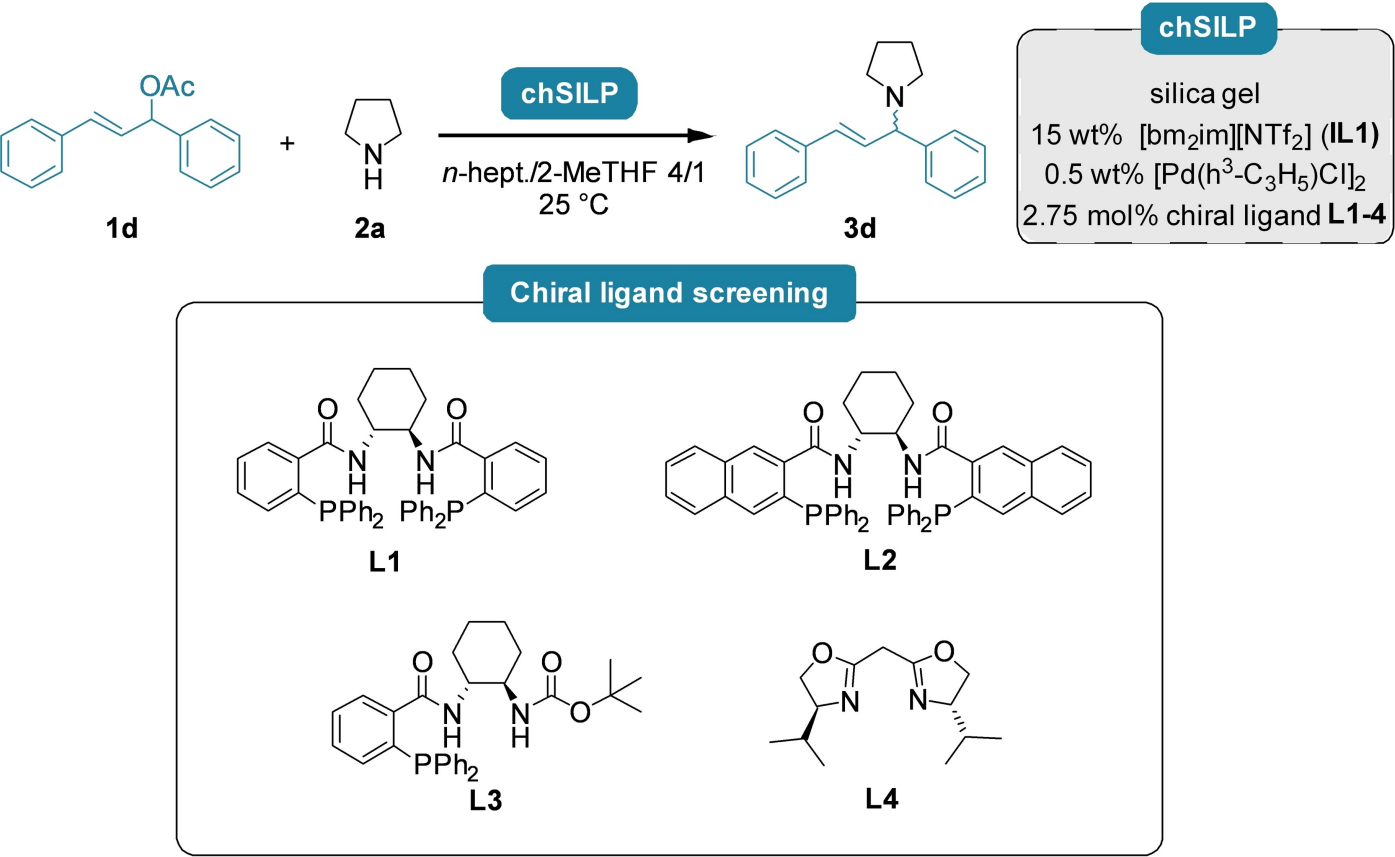					
Entry	Chiral ligand	Reaction in slurry phase		Reaction in flow	
		Conv. [%]^[a]^	ee [%]^[b]^	Conv. [%]^[a]^	ee [%]^[b]^
1	**L1**	44	44	39	30
2	**L2**	17	52	62	54
3	**L3**	70	65	81	74
4	**L4**	52	36	n.r.	n. d.

Reactions were carried out on 0.6 mmol scale using 1.0 equiv. allylic acetate **1 d** and 2.0 equiv. pyrrolidine (**2 a**) with 450 mg of **chSILP** (15 wt % **IL1**, 0.5 wt % [Pd(C_3_H_5_)Cl]_2_ and 2.75 wt % chiral ligand **L1**–**4**) in *n*‐heptane/2‐MeTHF 4/1. Slurry‐phase reactions were carried out in 8 mL screw‐cap vials at 25 °C for 24 hours. Continuous‐flow reactions were carried out at 25 °C with a residence time of 30 minutes. [a] Determined by GC‐MS analysis, no side‐product formation was observed. [b] Determined by chiral HPLC analysis using Daicel Chiralpak® IA‐3 column.

We then investigated the possible catalytic effect of the ionic liquid matrix. Using the palladium‐precursor [Pd(C_3_H_5_)Cl]_2_ and the chiral ligand **L3**, we prepared a series of **chSILP** catalysts featuring various hydrophobic ionic liquids. The introduction of **IL4** could not outperform the result with **IL1**, indicating that the ionic liquid chain length has basically no effect on the reactivity and on the level of stereodiscrimination (Table 3, entries 1 vs. 3). Inspired by the findings of Trost and co‐workers on the beneficial effect of tetraalkyl ammonium salts for *C*‐allylations, we also probed different quaternary ammonium salt‐based ILs (**IL3** and **IL5**–**6**). The **chSILP** catalysts prepared with the tetrahexylammonium‐ and DABCO‐based hydrophobic ionic liquids **IL3** and **IL6** could not improve the enantioselectivity; meanwhile, only moderate conversion was observed (Table 3, 1 vs. entries 2 and 5). When using the hydrophilic **IL5**, the corresponding **chSILP** catalyst leached entirely during the continuous‐flow pre‐conditioning phase (Table 3, entry 4), further underlining the importance of highly hydrophobic, NTf_2_‐based ionic liquids to tackle leaching issues efficiently.

**Table 3:** Effect of different ionic liquids for the continuous‐flow asymmetric *N*‐allylation of pyrrolidine (**2 a**) with **1 d** using **chSILP** catalysts.

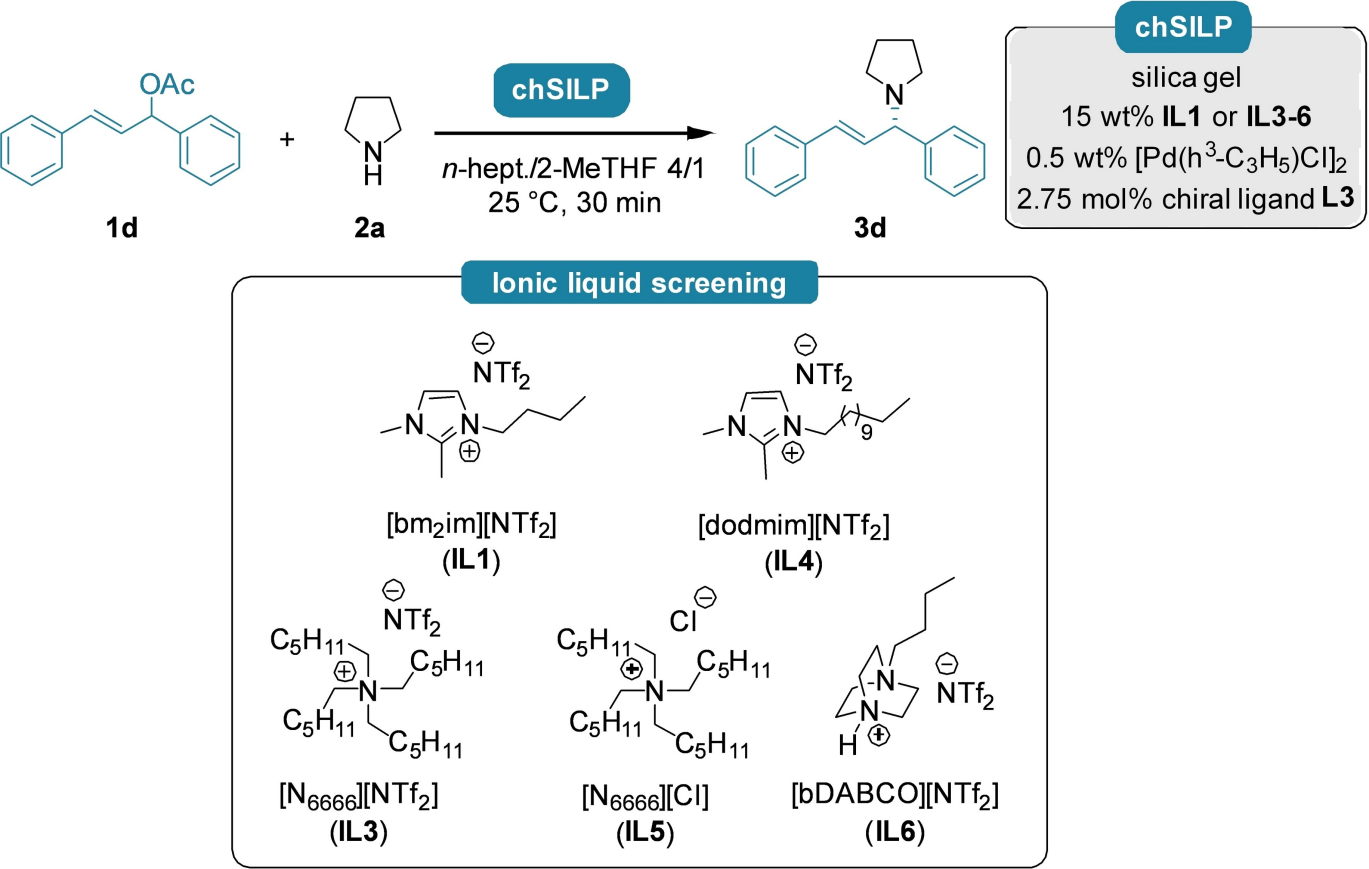			
Entry	Ionic liquid for chSILP preparation	Conv. [%]^[a]^	ee [%]^[b]^
1	**IL1**	81	74
2	**IL3**	54	68
3	**IL4**	76	73
4^[c]^	**IL5**	n. d.	n. d.
5	**IL6**	36	74

Reactions were carried out on 0.6 mmol scale using 1.0 equiv. allylic acetate **1 d** and 2.0 equiv. pyrrolidine (**2 a**) with 450 mg of **chSILP** (15 wt % **IL1** or **IL3**–**6**, 0.5 wt % [Pd(C_3_H_5_)Cl]_2_ and 2.0 wt % chiral ligand **L3**) in *n*‐heptane/2‐MeTHF 4/1 at 25 °C with a residence time of 30 minutes. [a] Determined by GC‐MS analysis, no side‐product formation was observed. [b] Determined by chiral HPLC analysis using a Daicel Chiralpak® IA‐3 column. n. d.: not determined. [c] Leaching of the ionic liquid (5 wt % of total IL amount) was detected by ^19^F NMR spectroscopy (limit of detection: >0.05 wt %).

**Scheme 2:**
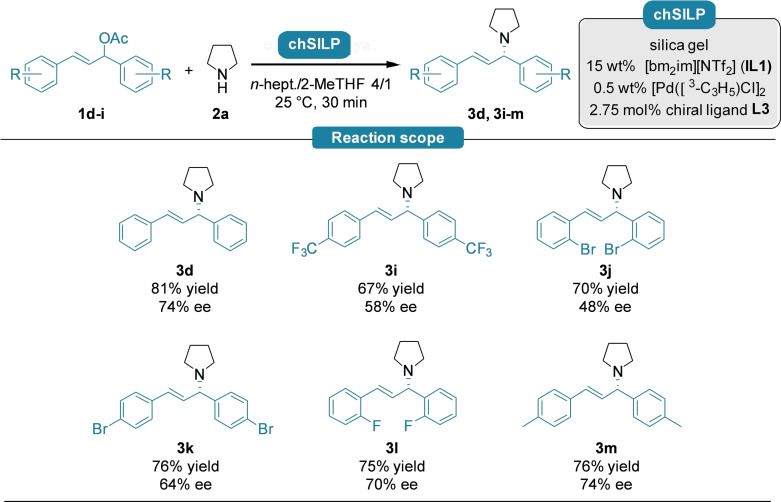
Reactions were carried out on 0.6 mmol scale using 1.0 equiv. substituted allylic acetate **1 d**–**i** and 2.0 equiv. pyrrolidine (**2 a**) with 450 mg of **chSILP** (15 wt % **IL1**, 0.5 wt % [Pd(C_3_H_5_)Cl]_2_ and 2.0 wt % chiral ligand **L3**) in *n*‐heptane/2‐MeTHF 4/1 at 25 °C with a residence time of 30 minutes. Yields refer to pure products isolated by column chromatography. The enantioselectivities were determined by chiral HPLC analysis using a Daicel Chiralpak® IA‐3 column.

With the optimized reaction conditions in hand, we investigated the asymmetric *N‐*allylation of pyrrolidine (**2 a**) with symmetrically substituted (*rac*)‐1,3‐diphenylallyl acetate derivatives featuring different steric and electronic properties (**1 d**–**i**). The corresponding enantioenriched *N*‐allylated products **3 d** and **3 i**–**m** were obtained in good to high yields and in good enantioselectivities (Scheme 2).

**Figure 3:**
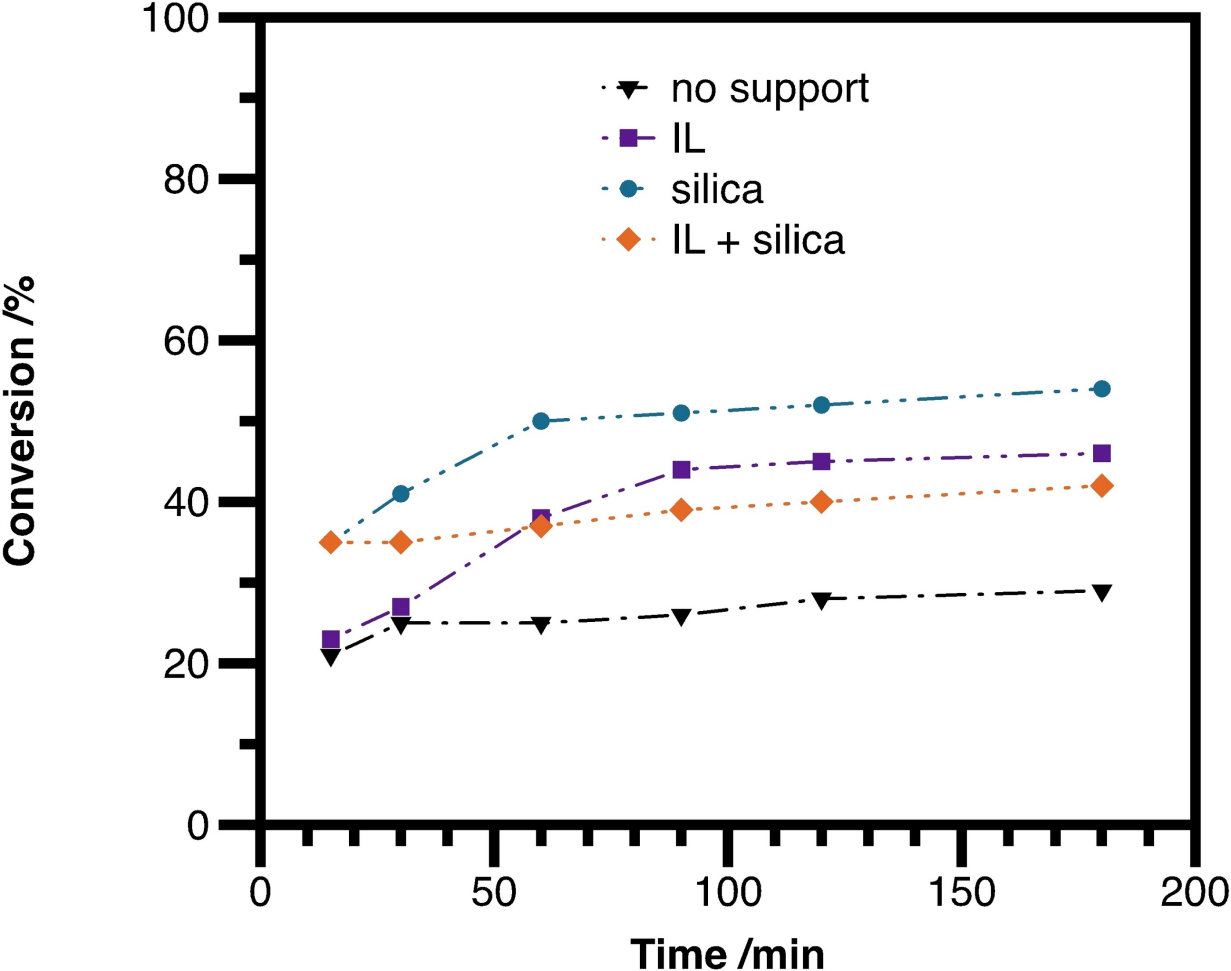
Effect of silica and ionic liquid on the *N*‐allylation of pyrrolidine (**2 a**) with **1 d** under batch conditions. Reactions were carried out on 0.6 mmol using 0.5 wt % [Pd(C_3_H_5_)Cl]_2_ and 2.0 wt % chiral ligand **L3** in *n*‐heptane/2‐MeTHF 4/1 at 25 °C.

In order to uncover the role of the silica and the ionic liquid in the catalytic system, control experiments for the reaction of pyrrolidine (**2 a**) with **1 d** have been carried out in batch mode using a **chSILP** with Pd/**L3** (Figure 3). The homogeneous phase allylation (black line) was very slow, providing only 25 % conversion within 3 hours. When performing the reaction in homogeneous phase in the presence of **IL1**, the reaction rate increases drastically (purple line). As the reaction proceeds *via* ionic mechanism, a polar co‐solvent, such as an ionic liquid might increase the reaction rate. Similarly, the addition of silica (blue line) also significantly accelerates the reaction. This might be explained by the acidity of the silanol groups, which can catalyze the cleavage of the acetate group. When using a **chSILP** (with **L3** and **IL1**, orange line); a slightly lowered reaction rate compared to the addition of pure IL and pure silica could be observed. The thin layer of ionic liquid on the silica surface might decrease the number of free silanol group sites, leading to a minor decrease in the activity of the catalyst system in batch mode. Nevertheless, using SILPs is crucial for continuous‐flow operations, as only the combination of these components can lead to efficient catalyst immobilization, therefore circumventing catalyst leaching.

In order to quantify the possible catalyst leaching for continuous‐flow reactions with chiral SILP‐systems, ^19^F NMR measurements were again carried out. When performing the *N*‐allylation of pyrrolidine (**2 a**) and **1 d** with the **chSILP** featuring the hydrophobic ionic liquid **IL1** and the chiral Pd/**L3** palladium complex, only a marginal 0.7 wt % of ionic liquid leaching (related to the total IL amount) and no detectable palladium‐complex leaching could be observed within an extended operation time of 3.5 hours.

## Conclusion

Herein we reported the use of palladium‐containing supported ionic liquid phases for the continuous‐flow allylic amination of various allylic acetate electrophiles. Apart from the fast and straightforward catalyst immobilization, this novel approach requires neither an additional base, nor inert atmosphere for efficient *N*‐allylation. Furthermore, the reactions could be carried out under mild conditions in benign reaction media. The optimal SILP catalyst could provide easy access to *N*‐allylation products within short reaction times (30 minutes). By extending the reaction time to 3.5 hours, such a SILP catalyst could maintain stable conversions, whereas only a marginal ionic liquid‐ and no palladium complex leaching could be observed. Furthermore, this approach could be used for the simple immobilization of chiral palladium complexes, enabling asymmetric *N*‐allylation reactions in continuous flow with good yields and enantioselectivities.

## Experimental Section

### Representative preparation of a chSILP catalyst

Allylpalladium(II)‐chloride dimer (0.055 mmol, 20 mg, 0.5 wt %), ligand **L3** (0.22 mmol, 110 mg, 2.75 wt %) and 1‐butyl‐2,3‐dimethylimidazolium bis(trifluoromethylsulfonyl)imide (**IL1**, 1.38 mmol, 600 mg, 15 wt %) were dissolved in anhydrous dichloromethane (1 mL) under argon atmosphere in a screw‐cap vial. The mixture was stirred for 30 minutes, and it was poured into a silica gel (3270 mg, 81.75 wt %) containing flask. The vial was rinsed with dichloromethane, and the suspension was stirred for 24 hours at room temperature under inert atmosphere. Then, the solvent was removed *in vacuo*, and the resulting fine powder was dried on high vacuum (0.4 mbar) at room temperature for several hours.

### Representative procedure for the continuous‐flow synthesis using chSILP catalysis

The corresponding diphenyl‐propenyl acetate derivative (**1 d**–**i**, 1.0 eq.) and pyrrolidine (**2 a**, 2.0 eq.) were dissolved in a mixture of *n*‐heptane: 2‐methyltetrahydrofuran (4 : 1) and it was stirred for 15 minutes. A cartridge was filled with the solid catalyst and was pre‐conditioned with the same solvent mixture. The reaction mixture was taken up with a syringe and pumped through the cartridge with the aid of a syringe pump. A flow rate which corresponds to 30 min residence time was chosen. The product was collected in a vial. After the whole volume was pumped through, the column was re‐washed with the solvent. The collected mixture was concentrated *in vacuo* and purified by flash column chromatography.

## Supporting Information

Additional references cited within the Supporting Information.[^33^, ^34^, ^35^, ^36^, ^37^, ^38^, ^39^, ^40^, ^41^, ^42^, ^43^, ^44^, ^45^, ^46^]

## Conflict of interest

The authors declare no conflict of interest.

1

## Supporting information

As a service to our authors and readers, this journal provides supporting information supplied by the authors. Such materials are peer reviewed and may be re‐organized for online delivery, but are not copy‐edited or typeset. Technical support issues arising from supporting information (other than missing files) should be addressed to the authors.

Supporting Information

## Data Availability

The data that support the findings of this study are available in the supplementary material of this article.
