# Supplementary material for: Supported Ionic Liquid Phase (SILP) Allylic Alkylation of Amines in Continuous Flow
Source: ChemCatChem. 2023 May 11;15(12):e202300381. doi: 10.1002/cctc.202300381 (PMC10947303; doi:10.1002/cctc.202300381)
Supplement: Supplementary file 1 — Supporting Information [file CCTC-15-0-s001.pdf]

ChemCatChem

Supporting Information

Supported Ionic Liquid Phase (SILP) Allylic Alkylation of Amines in Continuous Flow

Kristof Stagel, Ádám Márk Pálvölgyi, Clémence Delmas, Michael Schnürch, and Katharina Bica-Schröder*

Table of Contents

1. General remarks 3
2. Substrate synthesis for the non-enantioselective allylic alkylations (1a-d) 4
3. Substrate synthesis for the enantioselective allylic alkylations (1e-i) 6
4. Continuous-flow synthesis of allylic amines (3a-h) 9
   4.1 Synthesis of non-chiral supported ionic liquid phases (SILPs) 9
   4.2 General procedure for the synthesis of allylamines 3a-h 9
   4.3 Analytical data of allylamines 3a-h 10
5. Procedures for the synthesis of chiral ligands L1-L4 13
6. General procedure for the continuous-flow asymmetric allylation – synthesis of allylic amines 3d, 3i-m 16
   6.1 General procedure for the synthesis of racemic diphenyl-propenyl pyrrolidine derivatives 16
   6.2 Synthesis of chiral supported ionic liquid phases (chiral SILPs) 16
   6.3 General procedure for the synthesis for allylamines 3d, 3i-m 17
   6.4 Analytical data of allylamines 3d, 3i-m 17
7. TGA measurements of the SILP catalysts 21
8. SEM images of the chiral SILP catalyst 23
9. NMR spectra of allyl acetate derivatives 24
10. NMR spectra of amines 3a-h 33
11. NMR spectra of chiral ligands 40
12. NMR spectra of amines 3d, 3i-m 44
13. Chiral HPLC traces 51
14. References 57

1. General remarks

All purchased chemicals from commercial suppliers were used without further purification, unless otherwise noted. Dry CH_2Cl_2 was pre-distilled and desiccated on aluminum oxide columns (PURESOLV, Innovative Technology).

Column chromatography was performed on standard glass columns using Merck (40-60 µm) silica gel with pre-distilled solvents. For TLC analysis, pre-coated aluminium-backed plates were used (Merck, silica gel 60 F_{254}). All compounds were detected at 254 nm.

^{1}H-, ^{13}C-, ^{19}F- and ^{31}P-NMR spectra were recorded from $CDCl_3$ solutions on a Bruker Avance UltraShield 200 MHz (^{1}H: 200 MHz, ^{13}C: 50 MHz) or 400 MHz (^{1}H: 400 MHz, ^{13}C: 101 MHz, ^{19}F: 376 MHz) NMR instrument. Chemical shifts are reported in parts per million (ppm) and were calibrated to the residual solvent signal (e.g.: $CDCl_3$, ^{1}H: 7.26 ppm, ^{13}C: 77.0 ppm). Coupling constants are reported in hertz (Hz). The assignments are based on comparison with reported spectra.

Chiral HPLC measurements were carried out on a DIONEX UPLC equipped with a photoiodode array (PDA) plus detector (190-360 nm), using either a Daicel Chiralpak® IA-3 column (250 × 4.6 mm, 3 µm) or a Chiralcel® OD column (250 × 4.6 mm, 10 µm).

Optical rotation was measured on an Anton Paar MCP500 polarimeter at the specific conditions and the results were compared to literature values. Concentrations are given in g/100 mL.

HR-MS analysis was performed using a TC PAL system auto sampler, an Agilent 1100/1200 HPLC and an Agilent 6230 AJS ESI-TOF mass spectrometer. The spectra were recorded from methanolic solutions ($c = 3 – 50 \times 10^{-5}$ moldm^{-3}). The compounds were detected both in negative– and positive-ion mode.

Melting points were measured on an automated melting point system OPTI MELT of Stanford Research System and are uncorrected.

Continuous-flow experiments were performed with the aid of an Omnifit cartridge (bed length 10 cm). The reaction mixture was supplied with the aid of a New Era NE-1000 syringe pump.

2. Substrate synthesis for the non-enantioselective allylic alkylations (1a-d)

Cinnamyl acetate (**1a**) was purchased from commercial provider. Compounds **1c**[1] and **1d**[2] were synthesized according to literature procedures, respectively.

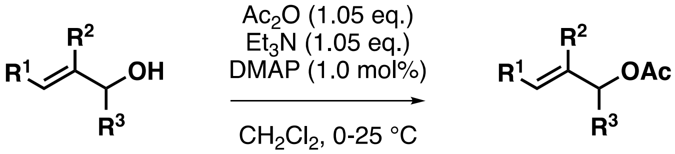

R^1 = H, R^2 = Me, R^3 = H
R^1 = Me, R^2 = H, R^3 = H
R^1 = Ph, R^2 = H, R^3 = Ph

1b R^1 = H, R^2 = Me, R^3 = H
1c R^1 = Me, R^2 = H, R^3 = H
1d R^1 = Ph, R^2 = H, R^3 = Ph

Figure S1. Synthesis of compounds **1a-d**

To a solution of the corresponding allylic alcohol (29.50 mmol, 1.0 equiv.) in dry CH_2Cl_2 (3.0 mL), acetic anhydride (2.52 mL, 30.94 mmol, 1.05 equiv.) and *N*,*N*-dimethylaminopyridine (36.2 mg, 0.295 mmol, 1.0 mol%) were added at 0 °C. A solution of Et_3N (4.30 mL, 30.94 mmol, 1.05 equiv.) in 4.0 mL dry CH_2Cl_2 was added dropwise at the same temperature and it was then allowed to warm up to room temperature. After 3 hours, the reaction mixture was poured onto 2M HCl/icewater and it was extracted with Et_2O (3 × 25 mL). The combined organic phases were washed with $NaHCO_3$ (1 × 20 mL) and brine (1 × 20 mL), dried over Na_2SO_4, filtered and concentrated *in vacuo*.

2-Methallyl acetate (1b)[1]

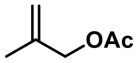

Prepared according to the general procedure using β-methallyl alcohol (2.50 mL, 29.50 mmol, 1.0 equiv.). *Concentrate in only to 200 mBar on the rotavapor, as the product is volatile! Kugelrohr* distillation (b.p. = 72 °C at 220 mBar) afforded **1b** as a colorless oil (2.43 g, 80% yield). **^{1}H NMR** (400 MHz, $CDCl_3$) δ 4.99 – 4.84 (m, 2H, $CH_3C(CH_2)C\mathit{H_2}OAc$), 4.49 (s, 2H, $CH_3C(C\mathit{H_2})CH_2OAc$ 2.09 (s, 3H, $C(O)C\mathit{H_3}$), 1.75 (d, *J* = 1.2 Hz, 3H, $C\mathit{H_3}C(CH_2)CH_2OAc$). **^{13}C NMR** (101 MHz, $CDCl_3$) δ 170.91, 140.07, 113.02, 67.88, 21.03, 19.62.

(*E*)-But-2-en-1-yl acetate (1c)[3]

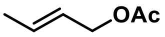

Prepared according to the general procedure using crotyl alcohol (3.41 mL, 40.0 mmol, 1.0 equiv.). *Concentrate in only to 200 mBar on the rotavapor, as the product is volatile! Kugelrohr* distillation (b.p. = 72 °C at 220 mBar) afforded **1c** as a colorless oil (3.11 g, 76% yield). **^{1}H NMR** (400 MHz, $CDCl_3$) δ 5.79 (dqt, *J* = 15.3, 6.4, 1.2 Hz, 1H, $CH_3C\mathit{H}CHCH_2OAc$), 5.58 (dtq, *J* = 16.3, 6.5, 1.6 Hz, 1H, $CH_3CHC\mathit{H}CH_2OAc$), 4.49 (dp, *J* = 6.5, 1.1 Hz, 2H, $CH_3CHCHC\mathit{H_2}OAc$), 2.05 (s, 3H, $C(O)C\mathit{H_3}$), 1.76 – 1.68 (m, 3H, $C\mathit{H_3}CHCHCH_2OAc$). **^{13}C NMR** (101 MHz, $CDCl_3$) δ 171.02, 131.60, 125.21, 65.37, 21.14, 17.89.

(*E*)-1,3-Diphenylallyl acetate (1d)[2]

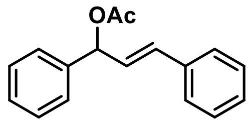

Prepared according to the general procedure using (*E*)-1,3-diphenylallyl alcohol (2.86 g, 28.0 mmol, 1.0 equiv.). *Kugelrohr* distillation (b.p. 165-170 °C at 0.4 mBar) afforded **1d** as yellowish, viscous oil (3.08 g, 87% yield). **^{1}H NMR** (400 MHz, $CDCl_3$) δ 7.33 – 7.10 (m, 10H, *H*-arom), 6.58 – 6.47 (m, 1H, Ar-C*H*=CH-CHO-Ar), 6.36 – 6.30 (m, 1H, Ar-CH=C*H*-CHO-Ar), 6.23 (dd, *J* = 15.8, 6.8 Hz, 1H, Ar-CH=CH-C*HO*-Ar), 2.02 (s, 3H, $C(O)C\mathit{H_3}$). **^{13}C NMR** (101 MHz, $CDCl_3$) δ 170.18, 139.38, 136.31, 132.74, 128.77, 128.72, 128.32, 128.20, 127.64, 127.19, 126.84, 76.28, 21.50.

3. Substrate synthesis for the enantioselective allylic alkylations (1e-i)

Compounds **1e-i** were prepared according the three-step procedure as follows:

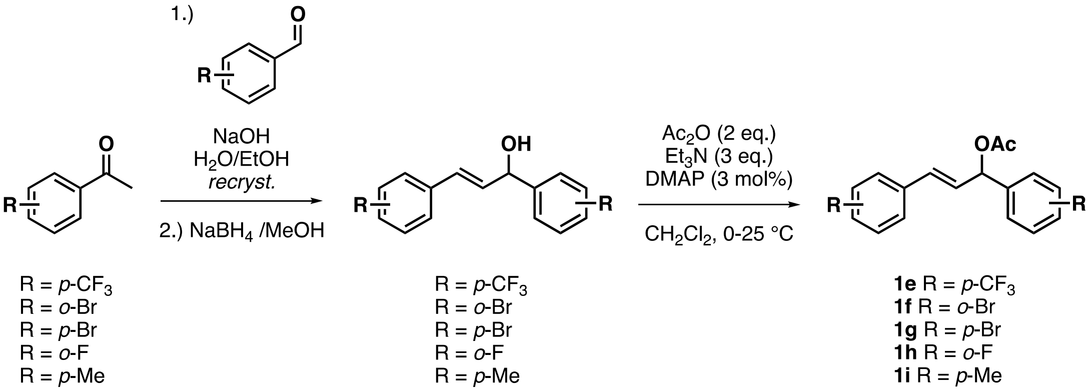

Figure S2. Synthesis of compounds **1e-i**

The corresponding chalcone derivatives[4] and alcohols[5] were prepared according to the literature procedures. Allylic acetates have been prepared according to the modified literature procedure.[2] The corresponding alcohol (1.0 equiv.), Et_3N (3.0 equiv.) and *N,N*-dimethylaminopyridine (0.036 equiv.) were transferred to a one-neck round-bottom flask and they were dissolved in anhydrous CH_2Cl_2 (0.25 M) under argon atmosphere. The mixture was cooled down with an ice bath, and it was stirred for 5 minutes at 0 °C. Then, acetic anhydride (2.0 equiv.) was added dropwise to the mixture with the aid of a syringe pump over 1 hour. After the addition was complete, the mixture was allowed to warm to room temperature and it was stirred overnight at room temperature. The reaction mixture was then quenched with water, and the aqueous phase was extracted with CH_2Cl_2 (2×). The combined organic extracts were washed with saturated $NaHCO_3$ solution (3×) and brine (1×), dried over anhydrous Na_2SO_4 and it was concentrated *in vacuo*. The residue was either used without further purification, purified *via* flash column chromatography, or distilled by *Kugelrohr* distillation.

(*E*)-1,3-Bis[*p*-(trifluoromethyl)phenyl]-2-propenyl acetate (1e)

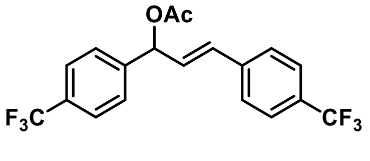

Prepared according to the general procedure from the corresponding alcohol (3.79 g, 11.0 mmol, 1.0 equiv.). Flash column chromatography (petroleum ether/EtOAc

10:1) afforded the product as colorless, viscous oil (3.56 g, 84% yield). **^{1}H NMR** (400 MHz, $CDCl_3$) δ 7.68 – 7.44 (m, 8H, *H*-arom), 6.68 (d, *J* = 15.8 Hz, 1H, Ar-C*H*=CH-CHO-Ar), 6.48 (d, *J* = 6.7 Hz, 1H, Ar-CH=C*H*-CHO-Ar), 6.39 (dd, *J* = 15.8, 6.6 Hz, 1H, Ar-CH=CH-C*HO*-Ar), 2.17 (s, 3H, C(O)C*H*$_3$). **^{13}C NMR** (101 MHz, $CDCl_3$) δ 169.93, 142.84, 139.43, 131.90, 129.45, 127.46, 127.06, 125.95, 125.92, 125.88, 125.84, 125.82, 125.78, 125.74, 75.24, 21.33. **^{19}F NMR** (376 MHz, $CDCl_3$) δ -62.63 – -62.66 (d, *J* = 11.0 Hz). **IR ATR (v_{max}/cm^{-1}):** 1742, 1323, 1229, 1120, 1066, 1016, 535. **HRMS (ESI):** calcd. for $C_{17}H_{15}F_6$ [M - OAc + H]$^+$: 333.1072; found: 333.1101.

(*E*)-1,3-Bis(*o*-bromophenyl)-2-propenyl acetate (1f)

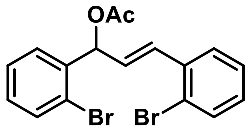

Prepared according to the general procedure from the corresponding alcohol (5.39 g, 14.0 mmol, 1.0 equiv.). Flash column chromatography (petroleum ether/EtOAc 30:1) afforded the product as colorless, viscous oil (4.79 g, 81% yield). **^{1}H NMR** (400 MHz, $CDCl_3$) δ 7.60 – 7.10 (m, 8H, *H*-arom), 7.04 – 6.97 (m, 1H, Ar-C*H*=CH-CHO-Ar), 6.79 (dd, *J* = 6.2, 1.4 Hz, 1H, Ar-CH=C*H*-CHO-Ar), 6.24 (dd, *J* = 15.8, 6.2 Hz, 1H, Ar-CH=CH-C*HO*-Ar), 2.18 (s, 3H, C(O)C*H*$_3$). **^{13}C NMR** (101 MHz, $CDCl_3$) δ 169.68, 138.45, 136.30, 133.23, 133.09, 133.00, 131.72, 130.50, 129.73, 129.43, 128.94, 128.37, 127.94, 127.59, 127.34, 127.09, 124.10, 122.98, 122.23, 74.78, 74.51, 35.54, 32.45, 21.28, 21.26. **IR ATR (v_{max}/cm^{-1}):** 1736, 1563, 1370, 1226, 962, 776, 632, 535. **HRMS (ESI):** calcd. for $C_{15}H_{11}Br_2$ [M - OAc - H]$^-$: 348.9225; found: 348.9221.

(*E*)-1,3-Bis(*p*-bromophenyl)-2-propenyl acetate (1g)

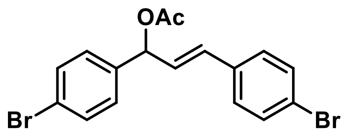

Prepared according to the general procedure from the corresponding alcohol (1.86 g, 5.0 mmol, 1.0 equiv.). Flash column chromatography (petroleum ether/EtOAc 10:1) afforded the product as yellow, viscous oil (1.56 g, 75% yield). **^{1}H NMR** (400 MHz, $CDCl_3$) δ 7.55 – 7.16 (m, 8H, *H*-arom), 6.58 – 6.51 (m, 1H, Ar-C*H*=CH-CHO-Ar), 6.38 – 6.34 (m, 1H, Ar-CH=C*H*-CHO-Ar), 6.28 (dd, *J* = 15.7, 6.6 Hz, 1H, Ar-CH=CH-C*HO*-Ar), 2.13 (s, 3H, C(O)C*H*$_3$). **^{13}C NMR** (101 MHz, $CDCl_3$) δ 169.87, 138.02, 134.91, 131.85, 131.77, 131.76, 131.57, 130.08, 128.78, 128.24, 127.75, 122.31, 122.11, 75.30, 37.47, 31.18, 21.28. **IR ATR (v_{max}/cm^{-1}):** 2786, 1490, 1116, 962, 919, 747, 690, 613, 545, 520. **HRMS (ESI):** calcd. for $C_{15}H_{11}Br_2$ [M - OAc - H]$^-$: 348.9242; found: 348.9249.

(*E*)-1,3-Bis(*o*-fluorophenyl)-2-propenyl acetate (1h)

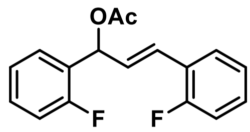

Prepared according to the general procedure from the corresponding alcohol (2.01 g, 8.0 mmol, 1.0 equiv.). Flash column chromatography (petroleum ether/EtOAc 8:1) afforded the product as colorless, viscous oil (1.65 g, 70% yield). **^{1}H NMR** (400 MHz, $CDCl_3$) δ 7.50 – 6.95 (m, 8H, *H*-arom), 6.80 (d, *J* = 16.1 Hz, 1H, Ar-C*H*=CH-CHO-Ar), 6.70 (d, *J* = 6.6 Hz, 1H, Ar-CH=C*H*-CHO-Ar), 6.47 (ddd, *J* = 16.1, 6.7, 0.9 Hz, 1H, Ar-CH=CH-C*HO*-Ar), 2.15 (s, 3H, C(O)CH_3). **^{13}C NMR** (101 MHz, $CDCl_3$) δ 169.85, 161.82, 158.96, 130.09, 130.00, 129.60, 129.51, 128.89, 128.45, 127.96, 125.38, 124.50, 124.23, 116.05, 115.83, 70.78, 21.33. **^{19}F NMR** (376 MHz, $CDCl_3$) δ -117.21, -117.26 (d, *J* = 19.1 Hz). **IR ATR (v_{max}/cm^{-1}):** 1740, 1488, 1456, 1371, 1222, 1019, 965, 753, 536, 521. **HRMS (ESI):** calcd. for $C_{15}H_{11}F_2$ [M - OAc - H]$^-$: 229.0912; found: 229.0909.

(*E*)-1,3-Bis(*p*-tolyl)-2-propenyl acetate (1i)

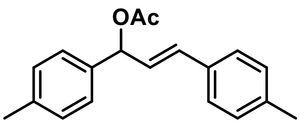

Prepared according to the general procedure from the corresponding alcohol (2.25 g, 22.0 mmol, 1.0 eq.). Product was obtained as orange, viscous oil (2.72 g, 88% yield) and was used without further purification. **^{1}H NMR** (400 MHz, $CDCl_3$) δ 7.35 – 7.06 (m, 8H, *H*-arom), 6.65 – 6.55 (m, 1H, Ar-C*H*=CH-CHO-Ar), 6.41 (d, *J* = 6.9 Hz, 1H, Ar-CH=C*H*-CHO-Ar), 6.30 (dd, *J* = 15.8, 6.8 Hz, 1H, Ar-CH=CH-C*HO*-Ar), 2.34 (d, *J* = 10.4 Hz, 6H, 2 × Ar-CH_3), 2.12 (s, 3H, C(O)CH_3). **^{13}C NMR** (101 MHz, $CDCl_3$) δ 170.23, 138.08, 138.03, 136.56, 133.60, 132.48, 129.42, 129.39, 127.21, 126.73, 126.72, 76.32, 21.54, 21.36, 21.32. **IR ATR (v_{max}/cm^{-1}):** 1736, 1514, 1369, 1229, 1017, 963, 632, 536, 516, 502. **HRMS (ESI):** calcd. for $C_{17}H_{17}$ [M - OAc]$^-$: 222.1403; found: 222.1373.

4. Continuous-flow synthesis of allylic amines (3a-h)

4.1 Synthesis of non-chiral supported ionic liquid phases (SILPs)

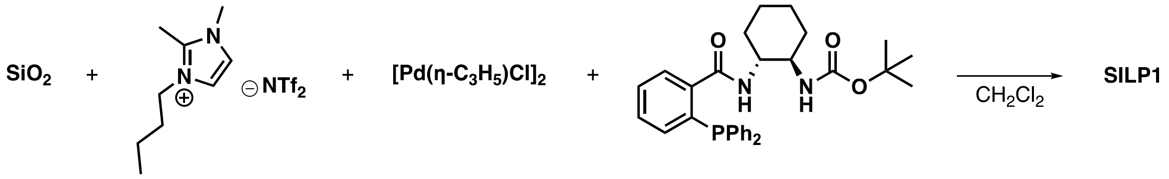

Figure S3. Synthesis of non-chiral SILPs

The catalytically active supported ionic liquid phase (SILP) was prepared as follows: Allylpalladium(II)-chloride dimer (0.055 mmol, 20 mg, 0.5 wt%), 1,3-Bis(diphenylphosphino)propane (0.22 mmol, 2.75 wt%) and 1-butyl-2,3-dimethylimidazolium bis(trifluoro-methylsulfonyl)imide ($[BMIMNTf_2]$, 1.38 mmol, 600 mg, 15 wt%) were dissolved in anhydrous CH_2Cl_2 (1mL) under argon atmosphere in a screw-cap vial (8 mL, VWR). The mixture was stirred for 30 minutes, and it was poured into a silica gel (Merck silica gel 60 Mesh, 3270 mg, 81.75 wt%) containing flask and it was rinsed with CH_2Cl_2. The suspension was shaken for 30 minutes (mechanical shaker, 700 rpm) and stirred for 24 hours at room temperature under inert atmosphere. Then, the solvent was removed *in vacuo,* and the resulting fine powder was dried on high vacuum (0.4 mbar) at room temperature for several hours.

4.2 General procedure for the synthesis of allylamines 3a-h

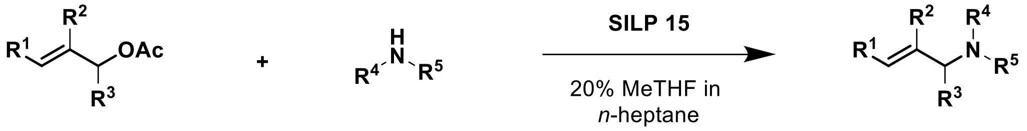

1a R^1 = Ph, R^2 = R^3 = H
1b R^1 = R^3 = H, R^2 = Me
1c R^1 = Me, R^2 = R^3 = H
1d R^1 = R^3 = Ph, R^2 = H

2a R^4 = R^5 = $-(CH_2)_4-$
2b R^4 = R^5 = $-(CH_2)_5-$
2c R^4 = R^5 = $-(CH_2)_2O(CH_2)_2-$
2d R^4 = R^5 = $-(CH_2)_2NMe(CH_2)_2-$
2e R^4 = H, R^5 = $-(CH_2)_7CH_3$

3a R^1 = Ph, R^2 = R^3 = H, R^4 = R^5 = $-(CH_2)_4-$
3b R^1 = R^3 = H, R^2 = Me , R^4 = R^5 = $-(CH_2)_4-$
3c R^1 = Me, R^2 = R^3 = H, R^4 = R^5 = $-(CH_2)_4-$
3d R^1 = R^3 = Ph, R^2 = H R^4 = R^5 = $-(CH_2)_4-$
3e R^1 = Ph, R^2 = R^3 = H, R^4 = R^5 = $-(CH_2)_5-$
3f R^1 = Ph, R^2 = R^3 = H, R^4 = R^5 = $-(CH_2)_2O(CH_2)_2-$
3g R^1 = Ph, R^2 = R^3 = H, R^4 = R^5 =$(CH_2)_2NMe(CH_2)_2-$
3h R^1 = Ph, R^2 = R^3 = H, R^4 = H, R^5 = $-(CH_2)_7CH_3$

Figure S4. Synthesis of compounds **3a-h**

The corresponding allylic acetate (1.0 equiv.) and amine (2.0 equiv.) were dissolved in a mixture of *n*-heptane: 2-methyltetrahydrofuran (4:1, 5 mL) and it was stirred for 15

minutes. A cartridge (100 × 3 mm, glass column) was filled with the solid supported catalyst (450 mg) and it was equilibrated with the same solvent mixture. The reaction mixture was taken up with a syringe (5 mL) and pumped through the cartridge with the aid of a syringe pump. A flow rate which corresponds to 30 min residence time was chosen. The product was collected in a vial. After the mixture was pumped through, the column was washed with the same solvent mixture again. The collected mixture was concentrated *in vacuo* and purified by flash column chromatography (figure S1).

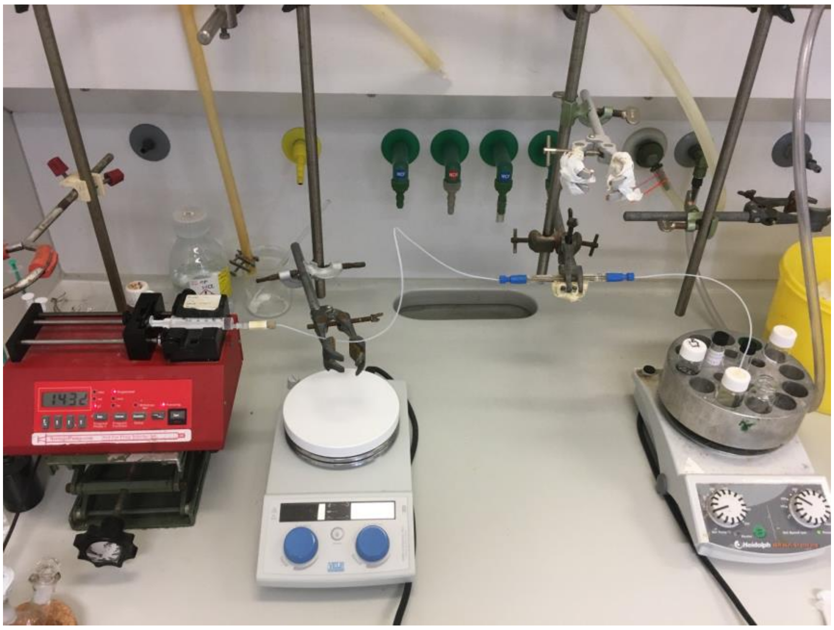

Figure S5. Set-up of the continuous-flow experiments

4.3 Analytical data of allylamines 3a-h

1-[(*E*)-3-Phenyl-2-propenyl]pyrrolidine (3a)[6]

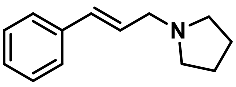

Following the general procedure (0.62 mmolar scale), purification by flash column chromatography (petroleum ether/ EtOAc/Et_3N 10:1:0.4), afforded the product as a yellow liquid (89 mg, 77% yield). **^{1}H NMR** (400 MHz, $CDCl_3$) δ 7.41 – 7.19 (m, 5H, *H*-arom), 6.54 (dt, *J* = 15.7, 1.4 Hz, 1H, ArC*H*=CH), 6.34 (dt, *J* = 15.8, 6.7 Hz, 1H, ArCH=C*H*), 3.27 (dd, *J* = 6.7, 1.4 Hz, 2H, ArCH=CHCH_2), 2.62 – 2.51 (m, 4H, N(CH_2CH$_2$)$_2$), 1.88 – 1.73 (m, 4H, N(CH$_2$CH_2)$_2$). **^{13}C NMR** (101 MHz, $CDCl_3$) δ 137.30, 131.95, 128.66, 127.92, 127.47, 126.43, 58.56, 54.23, 23.62.

1-(2-methylallyl)pyrrolidine (3b)

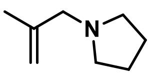

Due to its volatile nature, the product has not been isolated. GC-MS conversion: 67% (no by-product formation was observed).

(*E*)-1-(but-2-en-1-yl)pyrrolidine (3c)

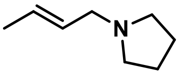

Due to its volatile nature, the product has not been isolated. GC-MS conversion: 51% (no by-product formation was observed).

1-[(*E*)-1,3-Diphenyl-2-propenyl]pyrrolidine (3d)[7]

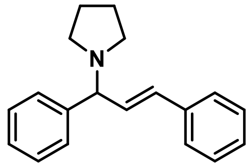

Following the general procedure (0.61 mmolar scale), purification by flash column chromatography (petroleum ether/EtOAc 5:1) afforded the product as a pale yellow solid (108 mg, 67% yield). **^{1}H NMR** (400 MHz, $CDCl_3$) δ 7.48 – 7.15 (m, 10H, *H*-arom), 6.62 – 6.52 (m, 1H, ArC*H*=CHCHNAr), 6.47 – 6.37 (m, 1H, ArCH=C*H*CHNAr), 3.82 – 3.70 (m, 1H ArCH=CHC*H*NAr), 2.65 – 2.35 (m, 4H, $N(CH_2CH_2)_2$), 1.79 (ddt, *J* = 7.1, 5.6, 2.6 Hz, 4H, $N(CH_2CH_2)_2$). **^{13}C NMR** (101 MHz, $CDCl_3$) δ 143.28, 137.22, 133.29, 129.96, 128.66, 128.61, 127.82, 127.49, 127.23, 126.53, 74.52, 53.27, 23.50.

1-[(*E*)-3-Phenyl-2-propenyl]piperidine (3e)[8]

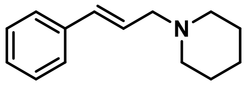

Following the general procedure (0.66 mmolar scale), purification by flash column chromatography (petroleum ether/ EtOAc/Et_3N 10:1:0.4) afforded the product as a yellow liquid (102 mg, 77% yield). **^{1}H NMR** (400 MHz, $CDCl_3$) δ 7.42 – 7.14 (m, 5H, *H*-arom), 6.50 (dt, *J* = 15.8, 1.4 Hz, 1H, ArC*H*=CH), 6.31 (dt, *J* = 15.9, 6.8 Hz, 1H, ArCH=C*H*), 3.12 (dd, *J* = 6.8, 1.3 Hz, 2H, ArCH=CHCH_2), 2.44 (s, 4H, $N(CH_2CH_2)_2CH_2$), 1.61 (p, *J* = 5.6 Hz, 4H, $N(CH_2CH_2)_2CH_2$), 1.45 (h, *J* = 5.4, 4.6 Hz, 2H, $N(CH_2CH_2)_2CH_2$). **^{13}C NMR** (101 MHz, $CDCl_3$) δ 137.23, 132.74, 128.66, 127.48, 127.41, 126.41, 62.04, 54.76, 26.13, 24.48.

4-[(*E*)-3-Phenyl-2-propenyl]morpholine (3f)[9]

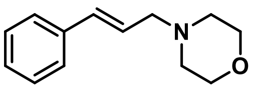

Following the general procedure (0.64 mmolar scale), purification by flash column chromatography (petroleum ether/ EtOAc/Et_3N 8:1:0.4) afforded the product as a yellow liquid (101 mg, 78% yield). **^{1}H NMR** (400 MHz, $CDCl_3$) δ 7.39 – 7.23 (m, 5H, *H*-arom), 6.54 (dt, *J* = 15.8, 1.5 Hz, 1H, ArC*H*=CH), 6.26 (dt, *J* = 15.9, 6.8 Hz, 1H, ArCH=C*H*), 3.75 (t, *J* = 4.7 Hz, 4H, $N(CH_2CH_2)_2O$), 3.17

(dd, J = 6.7, 1.4 Hz, 2H, ArCH=CHCH_2), 2.52 (t, J = 4.8 Hz, 4H, N(CH$_2$CH_2)$_2$O). **^{13}C NMR** (101 MHz, CDCl$_3$) δ 136.93, 133.61, 128.73, 127.74, 126.48, 67.11, 61.62, 53.83.

1-[(*E*)-3-Phenyl-2-propenyl]-4-methylpiperazine (3g)[10]

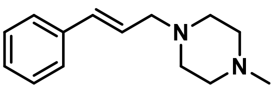

Following the general procedure (0.64 mmolar scale), purification by flash column chromatography (petroleum ether/ EtOAc/Et$_3$N 2.5:1:0.4) afforded the product as a yellow liquid (98 mg, 71% yield). **^{1}H NMR** (400 MHz, CDCl$_3$) δ 7.40 – 7.20 (m, 5H, *H*-arom), 6.53 (dt, J = 15.9, 1.4 Hz, 1H, ArC*H*=CH), 6.27 (dt, J = 15.9, 6.8 Hz, 1H, ArCH=C*H*), 3.17 (dt, J = 6.9, 1.4 Hz, 2H, ArCH=CHCH_2), 2.51 (s, 8H, N(CH_2CH_2)$_2$NCH$_3$), 2.30 (d, J = 2.1 Hz, 3H, N(CH$_2$CH$_2$)$_2$NCH_3). **^{13}C NMR** (101 MHz, CDCl$_3$) δ 137.05, 133.25, 128.69, 127.62, 126.71, 126.45, 61.18, 55.25, 53.31, 46.16.

[(*E*)-3-Phenyl-2-propenyl]octylamine (3h)[11]

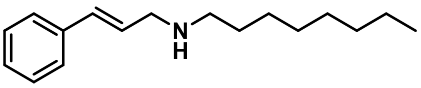

Following the general procedure (0.64 mmolar scale), purification by flash column chromatography (petroleum ether/ EtOAc/Et$_3$N 10:1:0.4) afforded the product as a yellowish oil (86 mg, 55% yield). **^{1}H NMR** (400 MHz, CDCl$_3$) δ 7.40 – 7.20 (m, 5H, *H*-arom), 6.53 (dt, J = 15.7, 1.6 Hz, 1H, ArC*H*=CH), 6.31 (dt, J = 15.9, 6.3 Hz, 1H, ArCH=C*H*), 3.42 (dd, J = 6.3, 1.4 Hz, 2H, ArCH=CHCH_2), 2.65 (t, J = 7.3 Hz, 2H, NCH_2(CH$_2$)$_6$CH$_3$), 1.52 (p, J = 7.2 Hz, 3H, NCH$_2$CH_2(CH$_2$)$_5$CH$_3$, N*H*), 1.35 – 1.23 (m, 10H, NCH$_2$CH$_2$(CH_2)$_5$CH$_3$), 0.94 – 0.83 (m, 3H, N(CH$_2$)$_7$CH_3). **^{13}C NMR** (101 MHz, CDCl$_3$) δ 137.32, 131.34, 128.67, 127.46, 126.40, 52.12, 49.70, 31.98, 30.29, 29.69, 29.42, 27.55, 22.81, 14.25.

5. Procedures for the synthesis of chiral ligands L1-L4

***N*-((1*R*,2*R*)-2-[*o*-(Diphenylphosphino)benzylamino]cyclohexyl)*o*-(diphenyl-phosphino)benzamide (L1)**

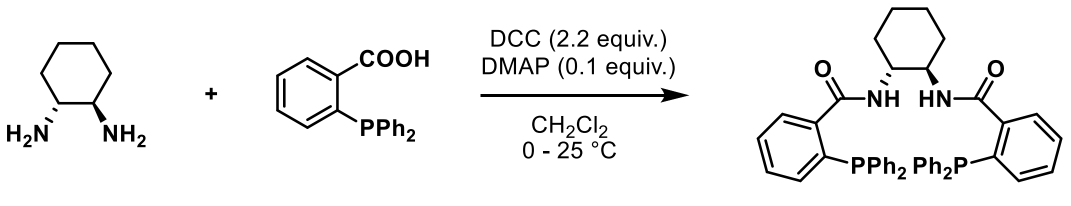

L1

Figure S6. Synthesis of compound **L1**

According to the literature procedure[12], (1*R*,2*R*)-1,2-cyclohexanediamine (0.53 g, 4.68 mmol, 1.0 equiv.), *o*-(diphenylphosphino)-benzoic acid (3.02 g, 9.38 mmol, 2.1 equiv.), DCC (2.13 g, 10.3 mmol, 2.2 equiv.) and DMAP (61 mg, 0.5 mmol, 0.1 equiv.) were dissolved in anhydrous CH_2Cl_2 (0.16 M) under argon atmosphere. The mixture was stirred at room temperature overnight. After completion, the majority of the formed DCU was removed *via* filtration, and the filter cake was washed with CH_2Cl_2. Then, the filtrate was washed with 10% HCl (3×), water (1×), saturated $NaHCO_3$ (3×), water (1×) and brine (1×). The organic phase was dried over Na_2SO_4, concentrated *in vacuo*, and the residue was purified by column chromatography (30% EtOAc in petroleum ether). The product was obtained as slightly yellowish solid (2.12 g, 66% yield). **^{1}H NMR** (400 MHz, $CDCl_3$) δ 7.57 (ddd, *J* = 5.5, 3.8, 2.1 Hz, 2H, *H*-arom), 7.41 – 7.13 (m, 24H, *H*-arom), 6.91 (ddd, *J* = 6.5, 3.9, 2.0 Hz, 2H, *H*-arom), 6.32 (d, *J* = 7.4, 2H, 2 × N*H*), 3.77 (ddt, *J* = 8.6, 5.8, 2.5 Hz, 2H, 2 × C*H*NH), 1.85 (d, *J* = 13.8 Hz, 2H, NHCHC*H*$_2$), 1.68 – 1.60 (m, 2H, NHCHC*H*$_2$), 1.37 – 1.15 (m, 2H, NHCHCH$_2$C*H*$_2$), 1.01 (t, *J* = 11.4 Hz, 2H, NHCHCH$_2$C*H*$_2$). **^{13}C NMR** (101 MHz, $CDCl_3$) δ 169.41, 141.05, 140.81, 137.91, 137.84, 137.80, 137.73, 136.80, 136.59, 134.42, 134.10, 133.90, 130.31, 128.92, 128.75, 128.69, 128.66, 128.62, 128.59, 128.52, 127.70, 127.65, 54.02, 32.12, 24.75. **^{31}P NMR** (162 MHz, $CDCl_3$) δ -9.66. **HRMS (ESI):** calcd. for $C_{44}H_{41}N_2O_2P_2$ $[M + H]^+$: 691.2638; found: 691.2638.

(1*R*,2*R*)-2-[*o*-(Diphenylphosphino)benzylamino]cyclohexylamino-*tert*-butyl-formylate (L3)

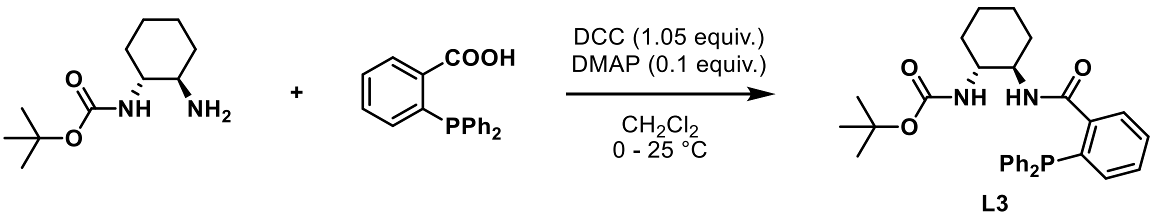

Figure S7. Synthesis of compound **L3**

According to the literature procedure,[13] *o*-(diphenylphosphino)benzoic acid (1.50 g, 4.9 mmol, 1.05 equiv.) was dissolved in 20 mL anhydrous CH_2Cl_2 at 0 °C. Then, *N*,*N*-dicyclohexylcarbodiimide (DCC, 1.01 g, 4.9 mmol, 1.05 equiv.) was added to the mixture and it was stirred for 15 minutes at 0 °C. To this, the solution of (1*R*,2*R*)-2-aminocyclohexylamino-*tert*-butylformylate (1.00 g, 4.7 mmol, 1.0 equiv.) and DMAP (57 mg, 0.47 mmol, 0.1 equiv.) in 25 mL anhydrous CH_2Cl_2 was slowly added. The mixture was allowed to warm up to room temperature and it was stirred overnight. After completion, the majority of the formed dicyclohexyl urea by-product (DCU) was removed *via* filtration, and the filter cake was washed with cold CH_2Cl_2. Then, the filtrate was washed with 0.5 M HCl (3×), saturated $NaHCO_3$ (3×) and water (2×). The organic phase was dried over Na_2SO_4 and concentrated *in vacuo*. Flash column chromatography (petroleum ether/EtOAc 4:1) afforded the product as a white solid (1.72 g, 73% yield). **^{1}H NMR** (400 MHz, $CDCl_3$) δ 7.64 – 7.58 (m, 1H, N*H*C(O)Ar), 7.40 – 7.19 (m, 12H, *H*-arom), 6.95 (dd, *J* = 7.9, 4.6 Hz, 1H, *H*-arom), 6.48 (s, 1H, *H*-arom), 4.81 (d, *J* = 8.6 Hz, 1H, N*H*C(O)O), 3.67 (tdd, *J* = 11.6, 8.1, 4.0 Hz, 1H, C*H*NHC(O)), 3.35 (d, *J* = 8.7 Hz, 1H, C*H*NHC(O)O), 2.05 – 1.96 (m, 1H, CH_{2a}CH), 1.95 – 1.88 (m, 1H, CH_{2b}CH), 1.72 (s, 1H, CH_{2a}CH), 1.64 (d, *J* = 10.6 Hz, 1H, CH_{2b}CH), 1.38 (s, 9H, $C(CH_3)_3$), 1.25 – 1.16 (m, 3H, CH_2CH_{2a}), 1.01 – 0.88 (m, 1H, CH_2CH_{2b}). **^{13}C NMR** (101 MHz, $CDCl_3$) δ 169.08, 156.80, 141.06, 140.83, 137.80, 134.38, 134.15, 134.12, 133.95, 133.92, 130.25, 128.74, 128.68, 128.61, 128.58, 128.54, 127.49, 127.45, 79.61, 65.99, 55.00, 54.20, 32.92, 32.28, 28.53, 25.09, 24.65, 15.41. **^{31}P NMR** (162 MHz, $CDCl_3$) δ -9.01. **HRMS (ESI):** calcd. for $C_{30}H_{36}N_2O_3P$ [M + H]$^+$: 503.2458; found: 503.2464.

(*S*)-2-([(*S*)-4-Isopropyl-4,5-dihydro-1,3-oxazol-2-yl]methyl)-4-isopropyl-4,5-dihydro-1,3-oxazole (L4)

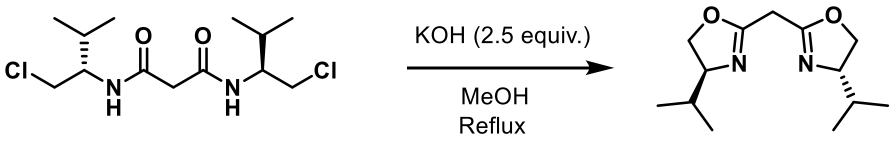

L4

Figure S8. Synthesis of compound **L4**

According to the literature procedure,[14] *N*,*N*-di[(*S*)-1-(chloromethyl)-2-methylpropyl]-malonamide (3.66 g, 12.0 mmol, 1.0 equiv.) and potassium hydroxide (1.62 g, 3.0 mmol, 2.5 equiv.) were dissolved in anhydrous methanol (0.12 M). The mixture was refluxed for 3 hours, then it was cooled down and poured onto water. It was extracted with CH_2Cl_2, washed with brine, dried over anhydrous Na_2SO_4 and concentrated *in vacuo*. Flash column chromatography (CH_2Cl_2/MeOH 20:1) afforded the product as a yellow gel (1.46 g, 52% yield). **^{1}H NMR** (400 MHz, $CDCl_3$) δ 4.31 – 4.23 (m, 2H, 2 × NC*H*), 4.06 – 3.89 (m, 4H, 2 × OCH_2C), 3.34 (s, *J* = 1.1 Hz, 2H, CCH_2C), 1.84 – 1.66 (m, 2H, 2 × C*H*(CH_3)$_2$), 1.02 – 0.84 (m, 12H, 2 × CH(CH_3)$_2$). **^{13}C NMR** (101 MHz, $CDCl_3$) δ 161.67, 72.34, 70.69, 32.19, 28.53, 18.80, 18.15.

6. General procedure for the continuous-flow asymmetric allylation – synthesis of allylic amines 3d, 3i-m

6.1 General procedure for the synthesis of racemic diphenyl-propenyl pyrrolidine derivatives

1,3-Bis(diphenylphophino)propane (0.02 equiv.) and allylpalladium(II) chloride dimer (0.01 equiv.) were dissolved in dichloromethane and the mixture was stirred for 15 minutes. Then, the corresponding diphenyl-propenyl acetate derivative (1.0 equiv.) dissolved in CH_2Cl_2 (0.5 M) was added to the mixture and stirred for further 15 minutes. Finally, pyrrolidine (2.0 equiv.) was added to the mixture and it was stirred at room temperature overnight. The mixture was concentrated *in vacuo* and purified by flash column chromatography, and they were used as racemic control for determining the enantioselectivity.

6.2 Synthesis of chiral supported ionic liquid phases (chiral SILPs)

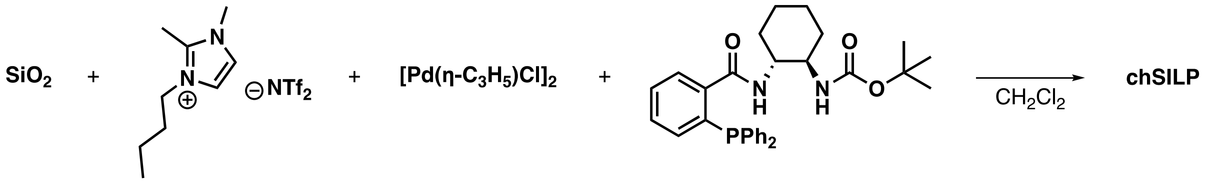

Figure S9. Synthesis of chiral SILPs (**chSILP**)

The catalytically active supported ionic liquid phase (SILP) was prepared as follows: Allylpalladium(II)-chloride dimer (0.055 mmol, 20 mg, 0.5 wt%), the corresponding chiral ligand (0.22 mmol, 2.75 wt%) and 1-butyl-2,3-dimethylimidazolium bis(trifluoromethylsulfonyl)imide ([BMIMNTf$_2$], 1.38 mmol, 600 mg, 15 wt%) were dissolved in anhydrous CH_2Cl_2 (1mL) in a screw-cap vial (8 mL, VWR). The mixture was stirred for 30 minutes, and it was poured into a silica gel (Merck silica gel 60 Mesh, 3270 mg, 81.75 wt%) containing flask, and it was rinsed with CH_2Cl_2. The suspension was shaken for 30 minutes (mechanical shaker, 700 rpm) and stirred for 24 hours at room temperature under argon atmosphere. Then, the solvent was removed *in vacuo,* and the resulting fine powder was dried on high vacuum (0.4 mbar) at room temperature for several hours.

6.3 General procedure for the synthesis for allylamines 3d, 3i-m

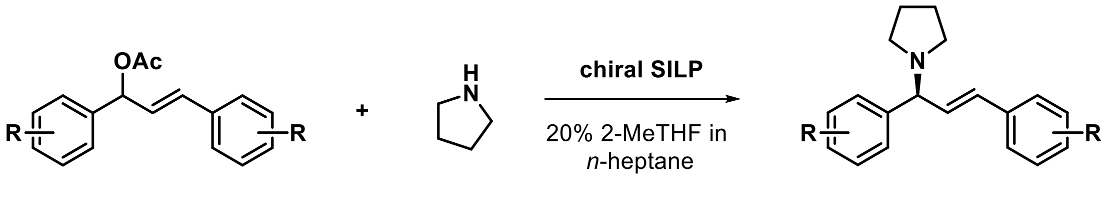

Figure S10. Synthesis of compounds **3d, 3i-m**

The corresponding diphenyl-propenyl acetate derivative (1.0 equiv.) and pyrrolidine (2.0 equiv.) were dissolved in a mixture of *n*-heptane: 2-methyltetrahydrofuran (4:1) and stirred for 15 minutes. A cartridge was filled with the solid supported catalyst (chiral SILP 15) and was equilibrated with the same solvent mixture. The reaction mixture was taken up with a syringe and pumped through the cartridge with the aid of a syringe pump. A flow rate which corresponds to 30 min residence time was chosen. The product was collected in a vial. The flow reaction setup was the same than on Figure S1 (page S10). After the mixture was pumped through, the column was washed with the same solvent mixture again. The collected mixture was concentrated *in vacuo* and purified by flash column chromatography.

6.4 Analytical data of allylamines 3d, 3i-m

1-[(*E*)-1,3-Diphenyl-2-propenyl]pyrrolidine (3d)

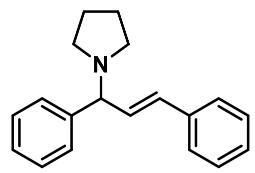

Following the general procedure (0.52 mmolar scale), purification by flash column chromatography (petroleum ether/EtOAc 5:1) afforded the product as a pale yellow solid (111 mg, 81% yield, 74% ee). $[\alpha]_{20}^{D}$: -3.57 (c = 1.0, CH_2Cl_2); **^{1}H NMR** (400 MHz, $CDCl_3$) δ 7.48 – 7.15 (m, 10H, *H*-arom), 6.62 – 6.52 (m, 1H, ArC*H*=CHCHNAr), 6.47 – 6.37 (m, 1H, ArCH=C*H*CHNAr), 3.82 – 3.70 (m, 1H ArCH=CHC*H*NAr), 2.65 – 2.35 (m, 4H, N(C*H*$_2$CH$_2$)$_2$), 1.79 (ddt, *J* = 7.1, 5.6, 2.6 Hz, 4H, N(CH$_2$C*H*$_2$)$_2$). **^{13}C NMR** (101 MHz, $CDCl_3$) δ 143.28, 137.22, 133.29, 129.96, 128.66, 128.61, 127.82, 127.49, 127.23, 126.53, 74.52, 53.27, 23.50. **IR ATR (v_{max}/cm^{-1}):** 2957, 2786, 1490, 1451, 1275, 1139,

1027, 986, 962, 896, 747, 690,613, 545. **HRMS (ESI):** calcd. for $C_{19}H_{22}N$ [M + H]$^+$: 264.1747; found: 264.1752. **Chiral HPLC:** Chiralpak® IA-3 column, *n*-heptane: ethanol 95:5, 0.35 mL/min, 25 °C, UV 220 nm, t_R (minor) = 12.8 min, t_R (major) = 13.2 min.

1-((*E*)-1,3-Bis[*p*-(trifluoromethyl)phenyl]-2-propenyl)pyrrolidine (3i)

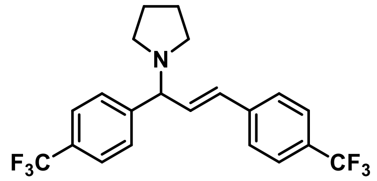

Following the general procedure (0.66 mmolar scale), purification by flash column chromatography (petroleum ether/EtOAc 7:1) afforded the product as an orange viscous liquid (177 mg, 67% yield, 58% ee). $[\alpha]_{20}^{D}$: +1.4 (c = 1.0, CH_2Cl_2); **^{1}H NMR** (400 MHz, $CDCl_3$) δ 7.84 – 7.14 (m, 8H, *H*-arom), 6.62 (d, *J* = 15.8 Hz, 1H, ArC*H*=CHCHNAr), 6.45 (dd, *J* = 15.6, 8.2 Hz, 1H, ArCH=C*H*CHNAr), 3.87 (d, *J* = 8.5 Hz, 1H, ArCH=CHC*H*NAr), 2.50 (d, *J* = 35.2 Hz, 4H, N(C*H*$_2$CH$_2$)$_2$), 1.81 (d, *J* = 6.1 Hz, 4H, N(CH$_2$C*H*$_2$)$_2$). **^{13}C NMR** (101 MHz, $CDCl_3$) δ 140.27, 131.79, 128.12, 126.74, 125.83, 125.79, 125.71, 125.68, 125.64, 125.60, 122.92, 73.86, 53.19, 23.51. **^{19}F NMR** (376 MHz, $CDCl_3$) δ -62.44, -62.55. **IR ATR (v_{max}/cm^{-1}):** 2964, 2795, 1616, 1417, 1322, 1163, 1121, 1066, 1017, 969, 835, 645, 632, 607, 551, 544, 535, 515. **HRMS (ESI):** calcd. for $C_{21}H_{20}F_6N$ [M + H]$^+$: 400.1557; found: 400.1558. **Chiral HPLC:** Chiralpak® IA-3 column, *n*-heptane: ethanol 99.5:0.5, 0.35 mL/min, 25 °C, UV 220 nm, t_R (minor) = 19.1 min, t_R (major) = 22.6 min.

1-[(*E*)-1,3-Bis(*m*-bromophenyl)-2-propenyl]pyrrolidine (3j)

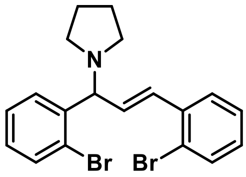

Following the general procedure (0.65 mmolar scale), purification by flash column chromatography (petroleum ether/EtOAc 5:1) afforded the product as a colorless viscous liquid (191 mg, 70% yield, 48% ee). $[\alpha]_{20}^{D}$: -6.2 (c = 1.0, CH_2Cl_2); **^{1}H NMR** (400 MHz, $CDCl_3$) δ 7.78 – 7.02 (m, 9H, *H*-arom, ArC*H*=CHCHNAr), 6.12 (dd, *J* = 15.7, 8.8 Hz, 1H, ArCH=C*H*CHNAr), 4.43 (d, *J* = 8.8 Hz, 1H, ArCH=CHC*H*NAr), 2.73 – 2.42 (m, 4H, N(C*H*$_2$CH$_2$)$_2$), 1.81 (d, *J* = 6.3 Hz, 4H, N(CH$_2$C*H*$_2$)$_2$). **^{13}C NMR** (101 MHz, $CDCl_3$) δ 137.08, 134.55, 133.08, 132.99, 129.76, 128.87, 128.47, 127.95, 127.50, 127.27, 123.83, 123.79, 71.48, 53.09, 23.57. **IR ATR (v_{max}/cm^{-1}):** 2964, 2789, 1464, 1437,1138, 1022, 965, 900, 748, 632, 540, 528, 521, 503. **HRMS (ESI):** calcd. for $C_{19}H_{20}Br_2N$ [M + H]$^+$: 419.9963; found: 419.9957. **Chiral HPLC:** Chiralcel® OD column, *n*-hexane: isopropanol: Et_2NH 99.8:0.2:0.2, 0.5 mL/min, 25 °C, UV 220 nm, t_R (major) = 13.3 min, t_R (minor) = 16.3 min.

1-[(*E*)-1,3-Bis(*p*-bromophenyl)-2-propenyl]pyrrolidine (3k)

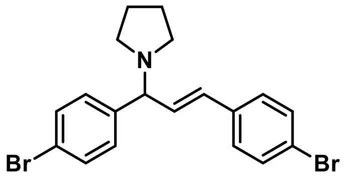

Following the general procedure (0.65 mmolar scale), purification by flash column chromatography (petroleum ether/EtOAc 7:1) afforded the product as a yellow solid (208 mg, 76% yield, 64% ee). **M.p.** 108-113 °C. $[\alpha]_{20}^{D}$**:** -17.2 (c=1.0, CH_2Cl_2); **^{1}H NMR** (400 MHz, $CDCl_3$) δ 7.56 – 7.14 (m, 8H, *H*-arom), 6.49 (d, *J* = 15.8 Hz, 1H, ArC*H*=CHCHNAr), 6.33 (dd, *J* = 15.8, 8.5 Hz, 1H, ArCH=C*H*CHNAr), 3.73 (d, *J* = 8.5 Hz, 1H, ArCH=CHC*H*NAr), 2.47 (d, *J* = 34.4 Hz, 4H, N(CH_2CH$_2$)$_2$), 1.79 (d, *J* = 6.3 Hz, 4H, N(CH$_2$CH_2)$_2$). **^{13}C NMR** (101 MHz, $CDCl_3$) δ 131.80, 131.64, 129.38, 127.98, 73.54, 53.04, 31.60, 23.36, 22.67, 14.14. **IR ATR (v_{max}/cm^{-1}):** 2961, 2877, 2792, 1485, 1070, 1007, 982, 814, 565. **HRMS (ESI):** calcd. for $C_{19}H_{20}Br_2N$ [M + H]$^+$: 419.9848; found: 419.9854. **Chiral HPLC:** Chiralpak® IA-3 column, *n*-heptane: ethanol 95:5, 0.35 mL/min, 25 °C, UV 220 nm, t_R (minor) = 14.3 min, t_R (major) = 15.3 min.

1-[(*E*)-1,3-Bis(*o*-fluorophenyl)-2-propenyl]pyrrolidine (3l)

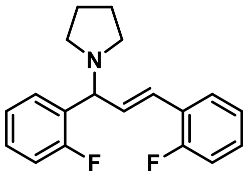

Following the general procedure (0.64 mmolar scale), purification by flash column chromatography (petroleum ether/EtOAc 7:1) afforded the product as yellow viscous liquid (144 mg, 75% yield, 70% ee). $[\alpha]_{20}^{D}$**:** +1.5 (c = 1.0, CH_2Cl_2); **^{1}H NMR** (400 MHz, $CDCl_3$) δ 7.68 – 6.93 (m, 8H, *H*-arom), 6.80 (d, *J* = 15.9 Hz, 1H, ArC*H*=CHCHNAr), 6.48 (dd, *J* = 15.9, 8.7 Hz, 1H, ArCH=C*H*CHNAr), 4.24 (d, *J* = 8.7 Hz, 1H, ArCH=CHC*H*NAr), 2.81 – 2.32 (m, 4H, N(CH_2CH$_2$)$_2$), 1.90 – 1.69 (m, 4H, N(CH$_2$CH_2)$_2$). **^{13}C NMR** (101 MHz, $CDCl_3$) δ 161.61, 159.17, 159.13, 129.33, 129.29, 128.91, 128.83, 128.55, 128.47, 127.53, 127.49, 124.49, 124.46, 124.16, 124.12, 123.05, 115.91, 115.77, 115.69, 115.55, 66.06, 66.05, 53.14, 23.49. **^{19}F NMR** (376 MHz, $CDCl_3$) δ -118.06, -118.66. **IR ATR (v_{max}/cm^{-1}):** 1966, 2791, 1486, 1456, 1268, 1229, 968, 753, 632, 535, 514, 503. **HRMS (ESI):** calcd. for $C_{19}H_{20}F_2N$ [M + H]$^+$: 300.1558; found: 300.1566. **Chiral HPLC:** Chiralpak® IA-3 column, *n*-heptane: ethanol 99.5:0.5, 0.35 mL/min, 25 °C, UV 220 nm, t_R (minor) = 14.3 min, t_R (major) = 15.5 min.

1-[(*E*)-1,3-Bis(*p*-tolyl)-2-propenyl]pyrrolidine (3m)

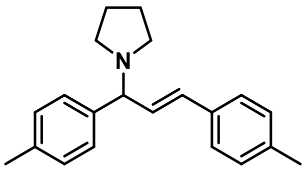

Following the general procedure (0.66 mmolar scale), purification by flash column chromatography (petroleum ether/EtOAc 5:1) afforded the product as a pale yellow solid (193 mg, 76% yield, 74% ee). **M.p.** 87-91 °C. $[\alpha]_{20}^{D}$**:** -8.8 (c = 1.0, CH_2Cl_2); **^{1}H NMR** (400 MHz, $CDCl_3$) δ 7.40 – 7.00 (m, 8H, *H*-arom), 6.52 (d, *J* = 15.8 Hz, 1H, ArC*H*=CHCHNAr), 6.36 (dd, *J* = 15.7, 8.6 Hz, 1H, ArCH=C*H*CHNAr), 3.71 (d, *J* = 8.5 Hz, 1H, ArCH=CHC*H*NAr), 2.49 (dtd, *J* = 43.9, 7.8, 6.9, 3.2 Hz, 4H, $N(CH_2CH_2)_2$), 2.32 (d, *J* = 8.4 Hz, 6H, 2 × Ar-CH_3), 1.91 – 1.73 (m, 4H, $N(CH_2CH_2)_2$). **^{13}C NMR** (101 MHz, $CDCl_3$) δ 140.42, 137.18, 136.74, 134.49, 132.40, 129.67, 129.32, 129.26, 127.70, 126.43, 74.24, 53.26, 23.48, 21.29, 21.23. **IR ATR (v_{max}/cm^{-1}):** 2973, 2874, 2780, 1509, 1122, 987, 906, 901, 589, 520. $C_{19}H_{20}F_2N$ **HRMS (ESI):** calcd. for $C_{21}H_{26}N$ $[M + H]^+$: 292.2267; found: 292.2285. **Chiral HPLC:** Chiralpak® IA-3 column, *n*-hexane: ethanol 99.6:0.4, 0.35 mL/min, 25 °C, UV 220 nm, t_R (minor) = 20.9 min, t_R (major) = 24.0 min.

7. TGA measurements of the SILP catalysts

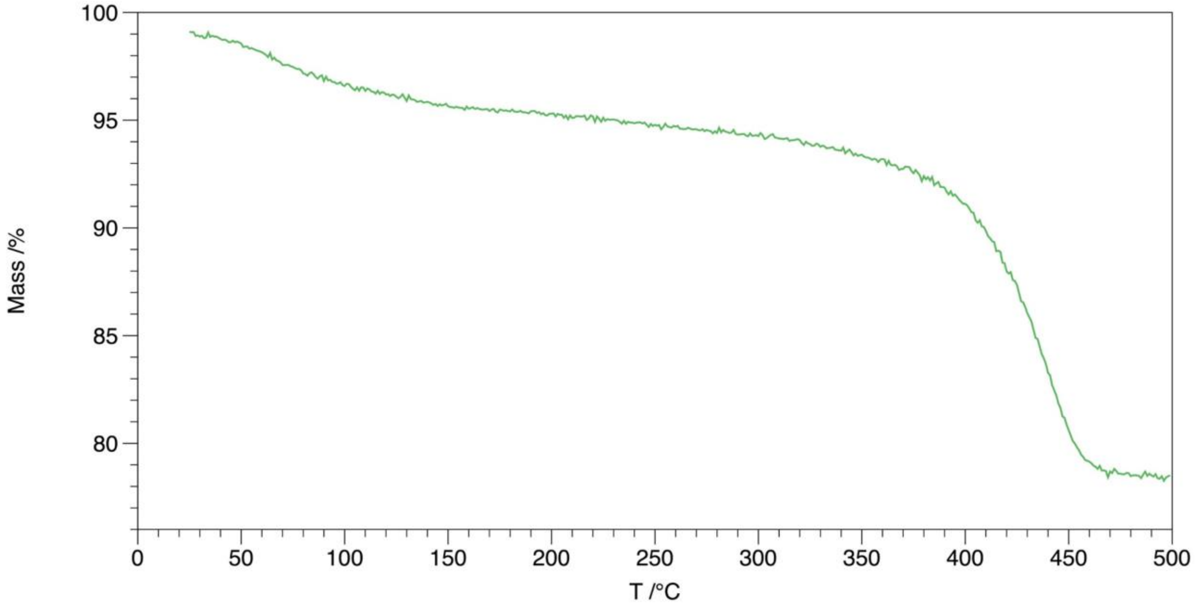

Figure S11. Thermogravimetric analysis of **SILP1**

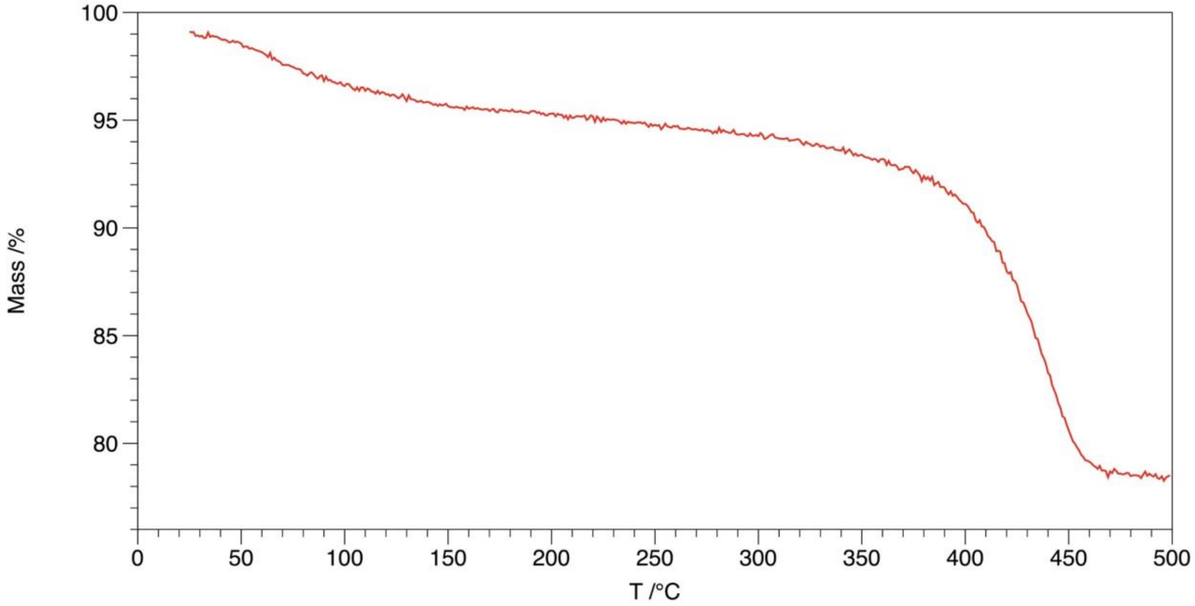

Figure S12. Thermogravimetric analysis of **chSILP** with chiral ligand **L1**

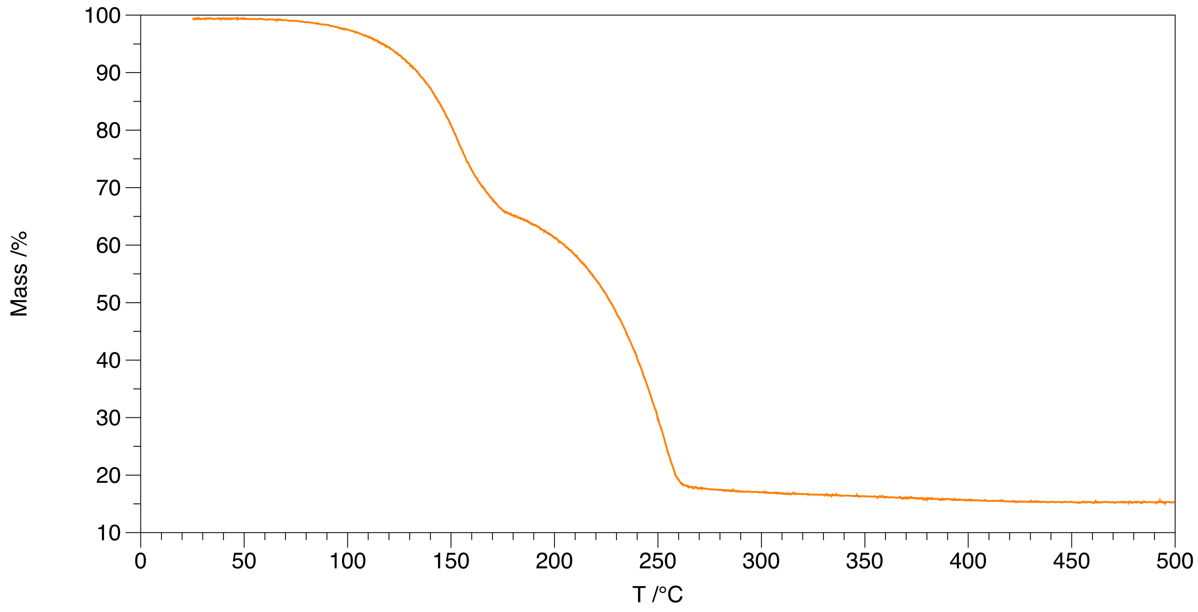

Figure S13. Thermogravimetric analysis of **3a**

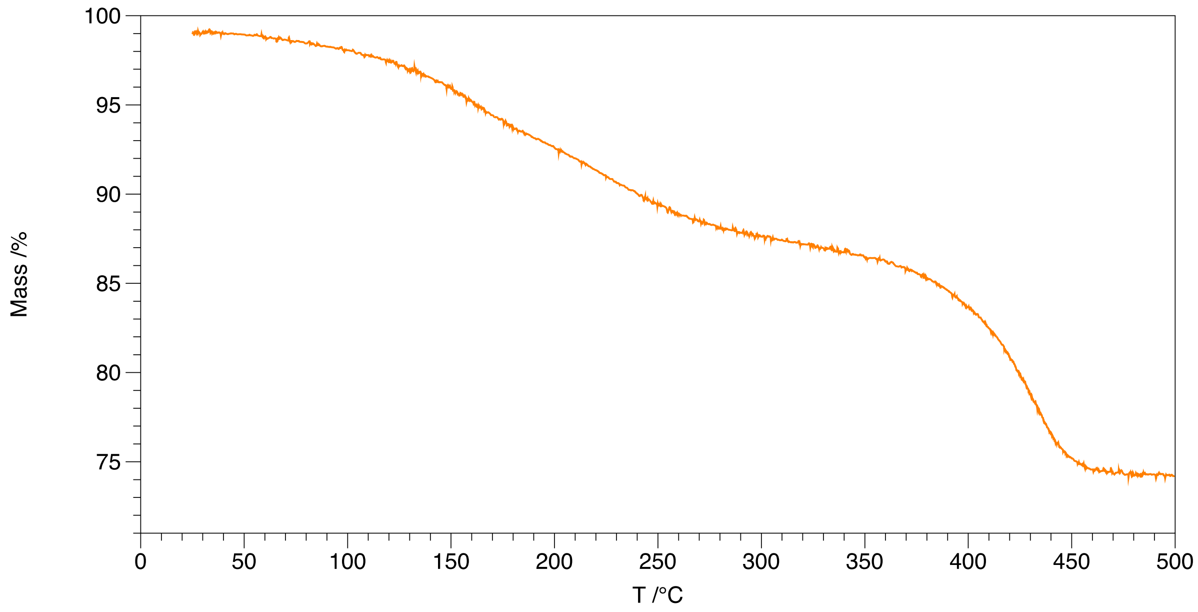

Figure S14. Thermogravimetric analysis of **SILP1** after the long-term experiment

8. SEM images of the chiral SILP catalyst

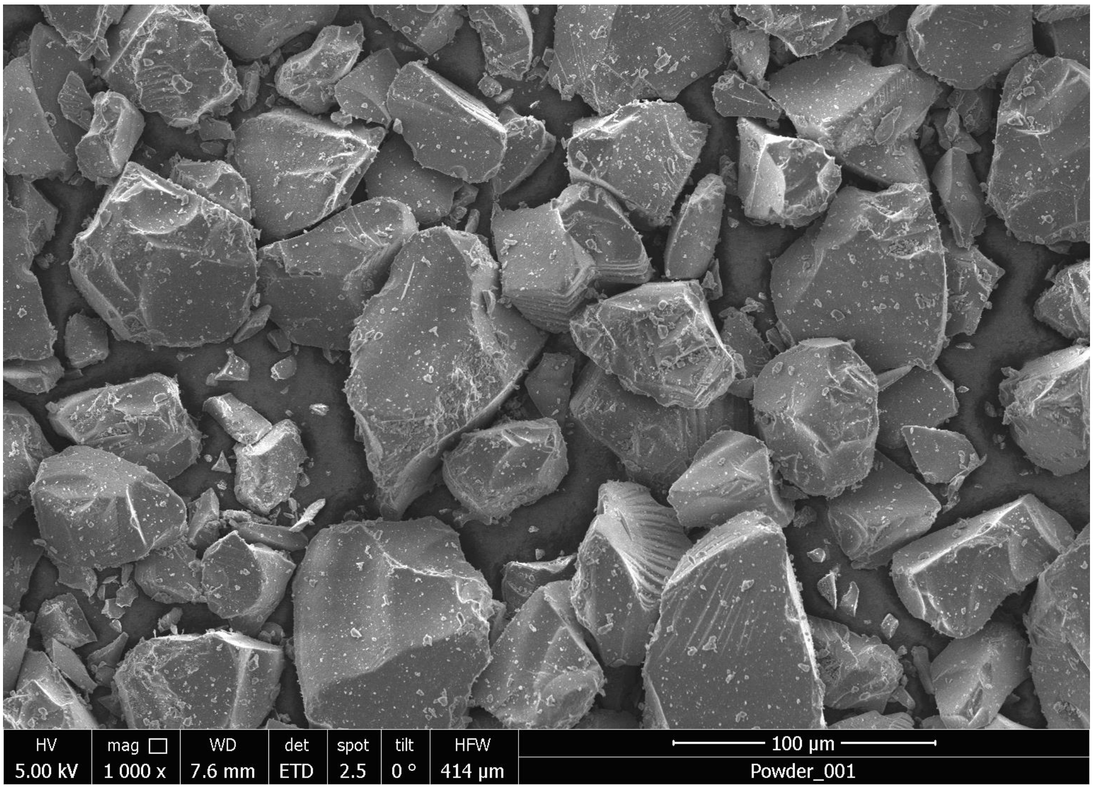

Figure S15. Scanning electron microscope image of **chSILP** with chiral ligand **L1**

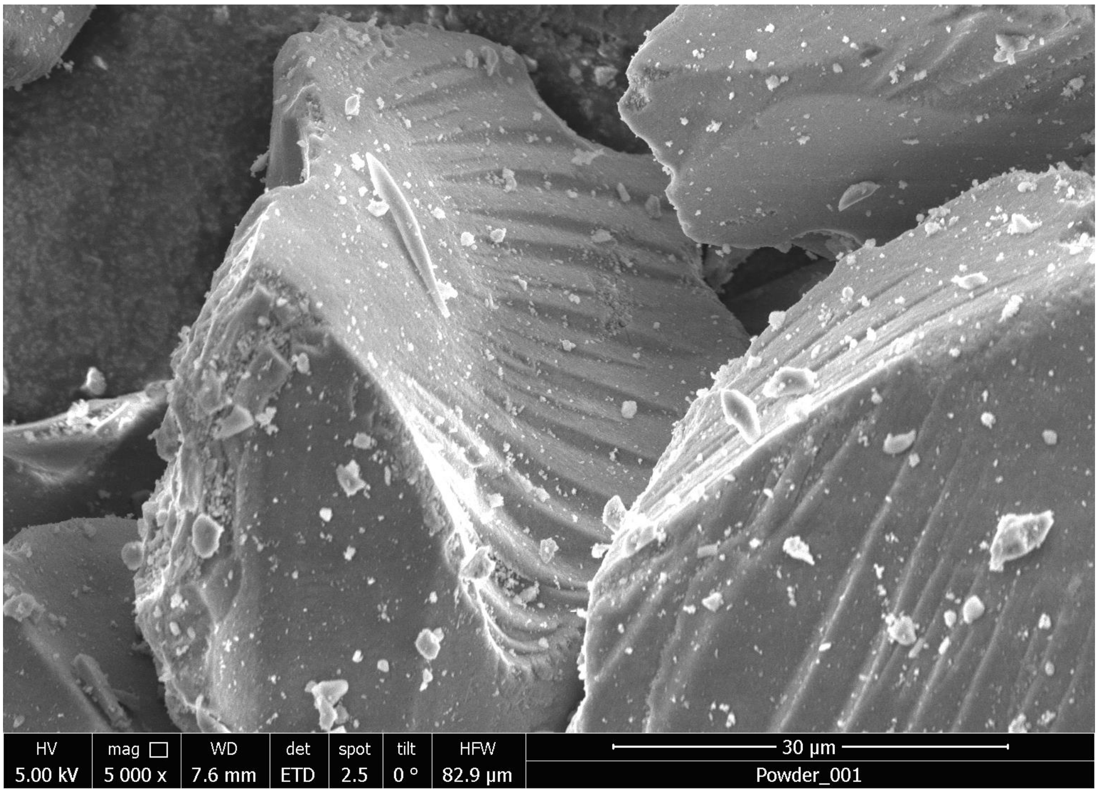

Figure S16. Scanning electron microscope image of **chSILP** with chiral ligand **L1**

9. NMR spectra of allyl acetate derivatives

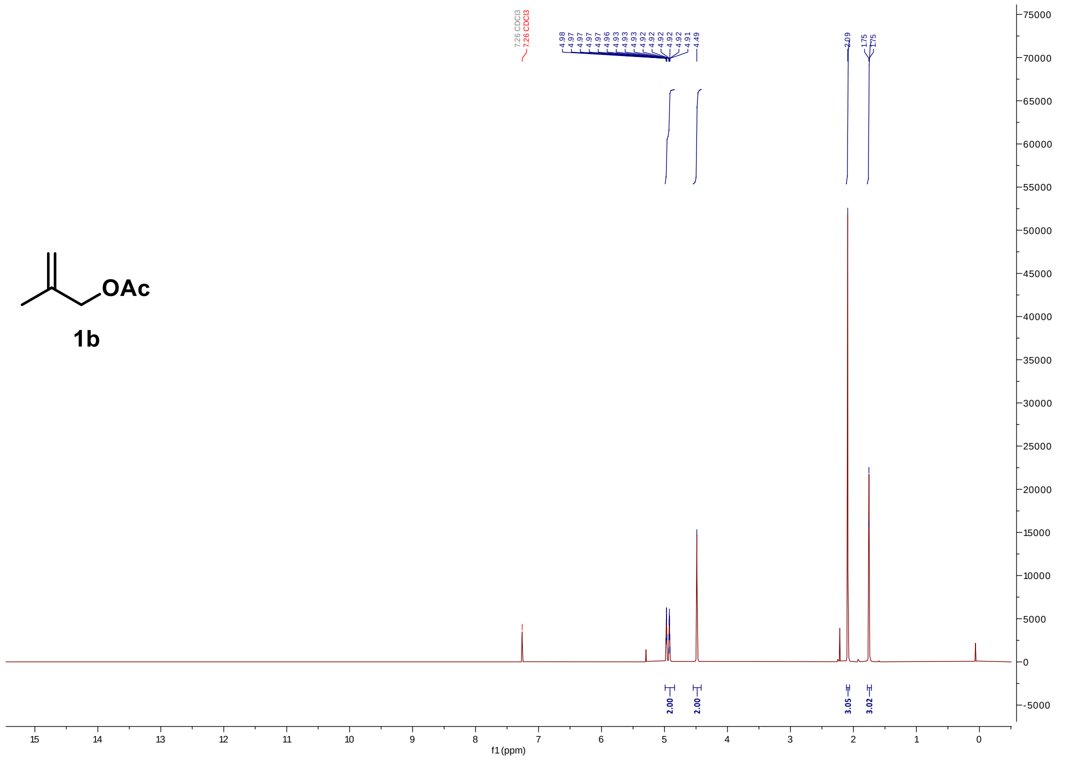

Figure S17. ^{1}H-NMR spectrum of **1b**

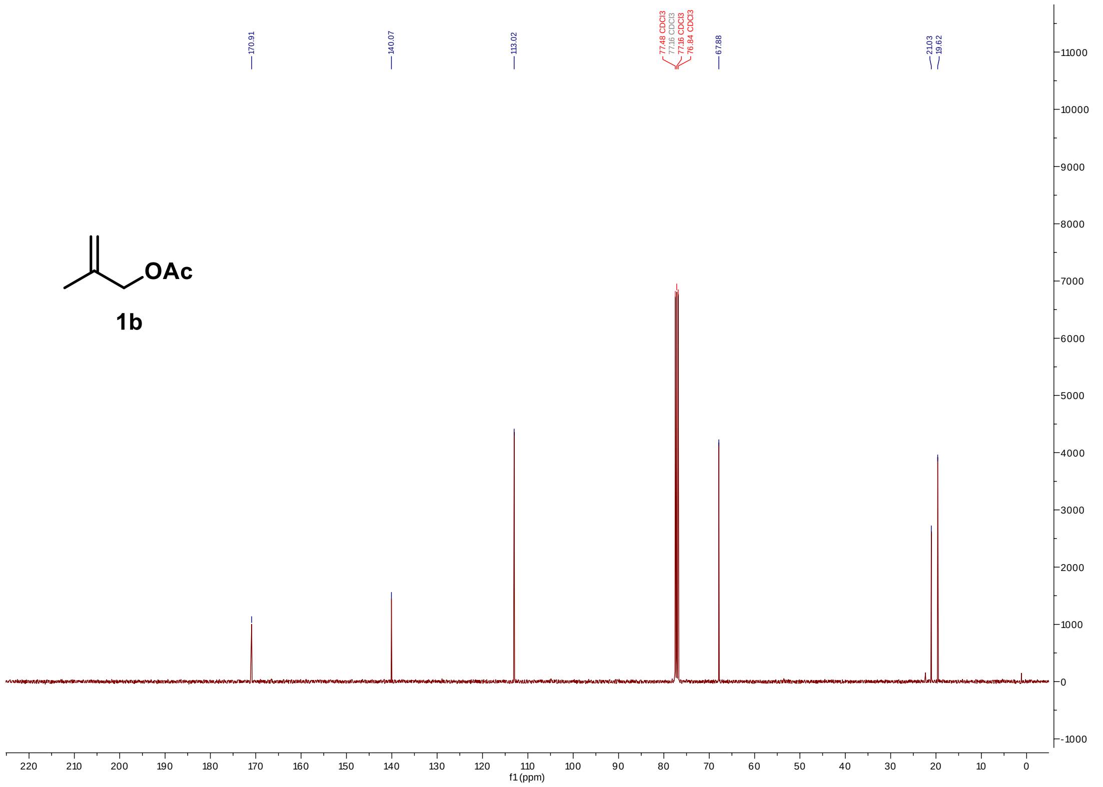

Figure S18.^{13}C-NMR spectrum of **1b**

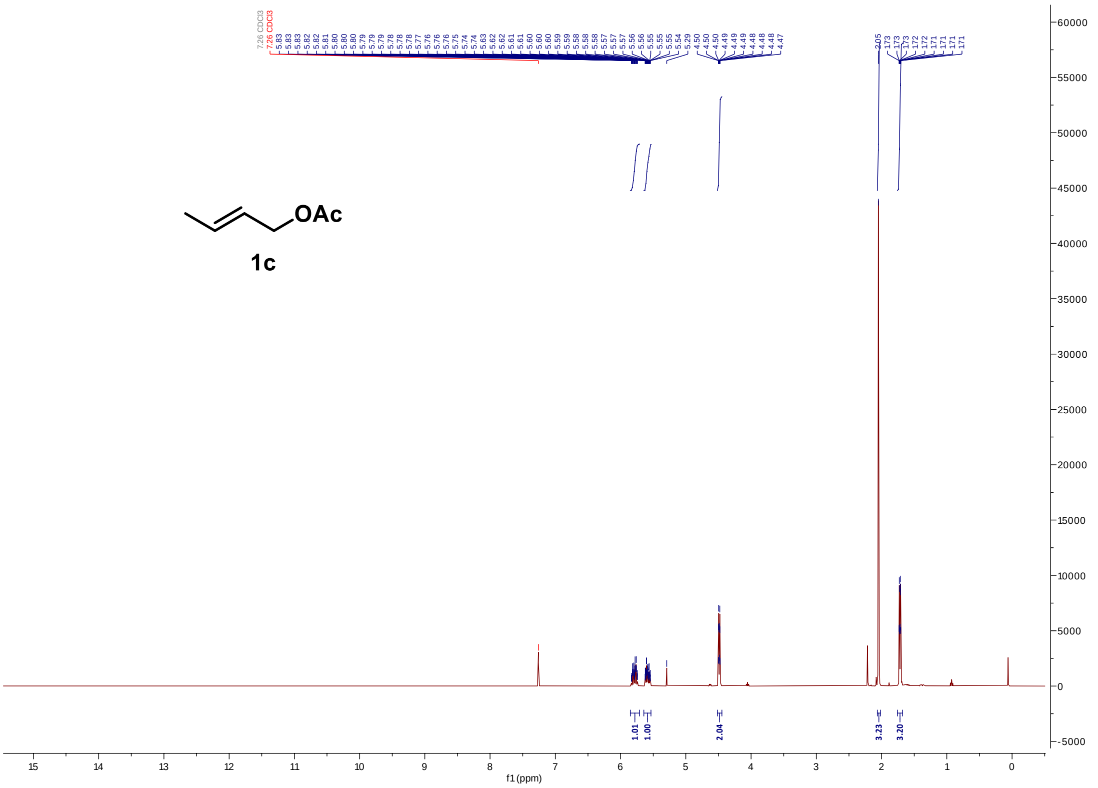

Figure S19. ^{1}H-NMR spectrum of **1c**

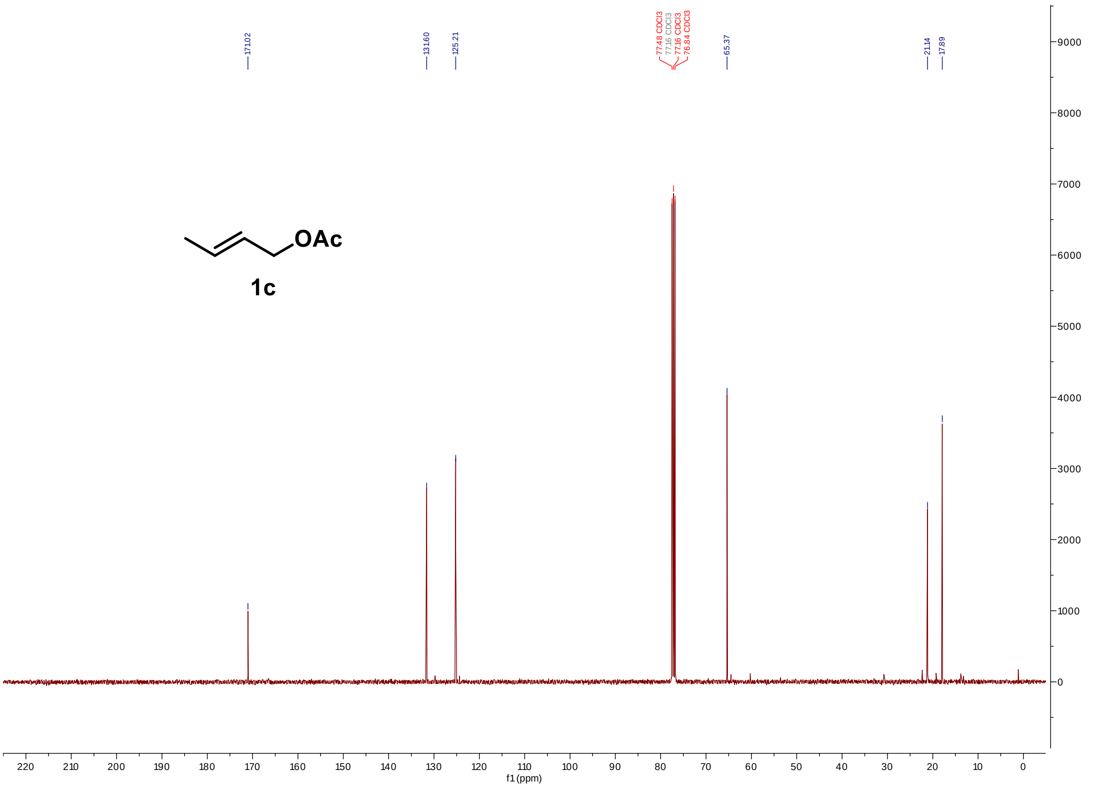

Figure S20.^{13}C-NMR spectrum of **1c**

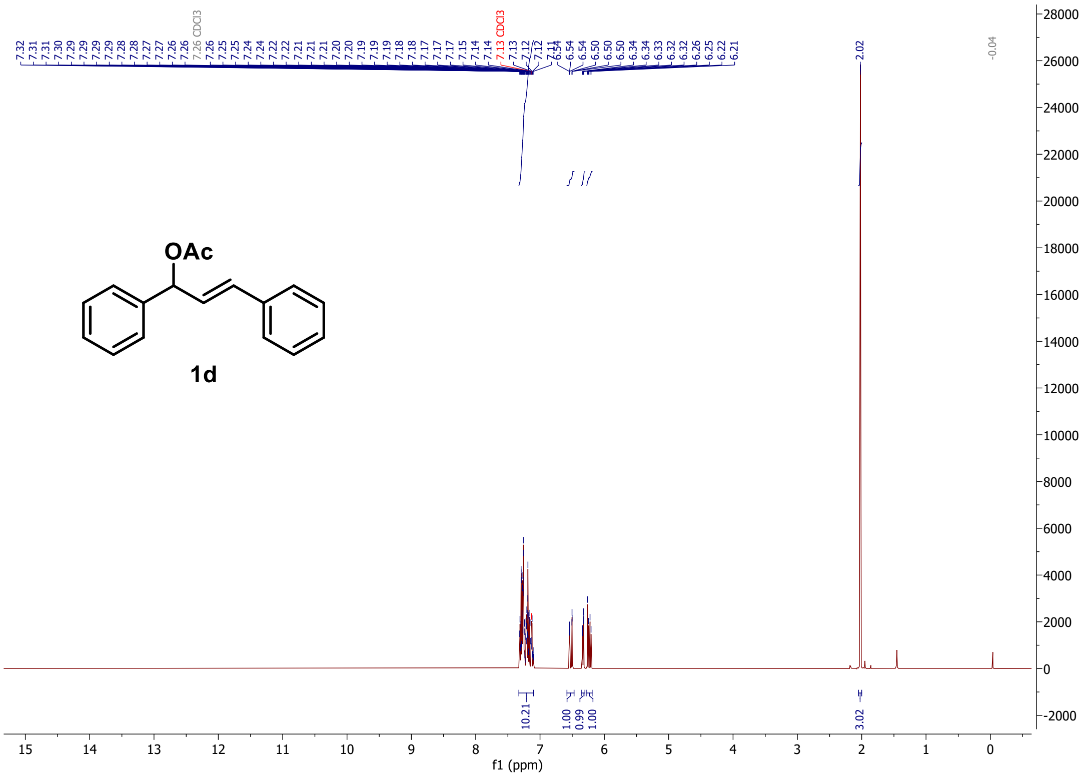

Figure S21. ^{1}H-NMR spectrum of **1d**

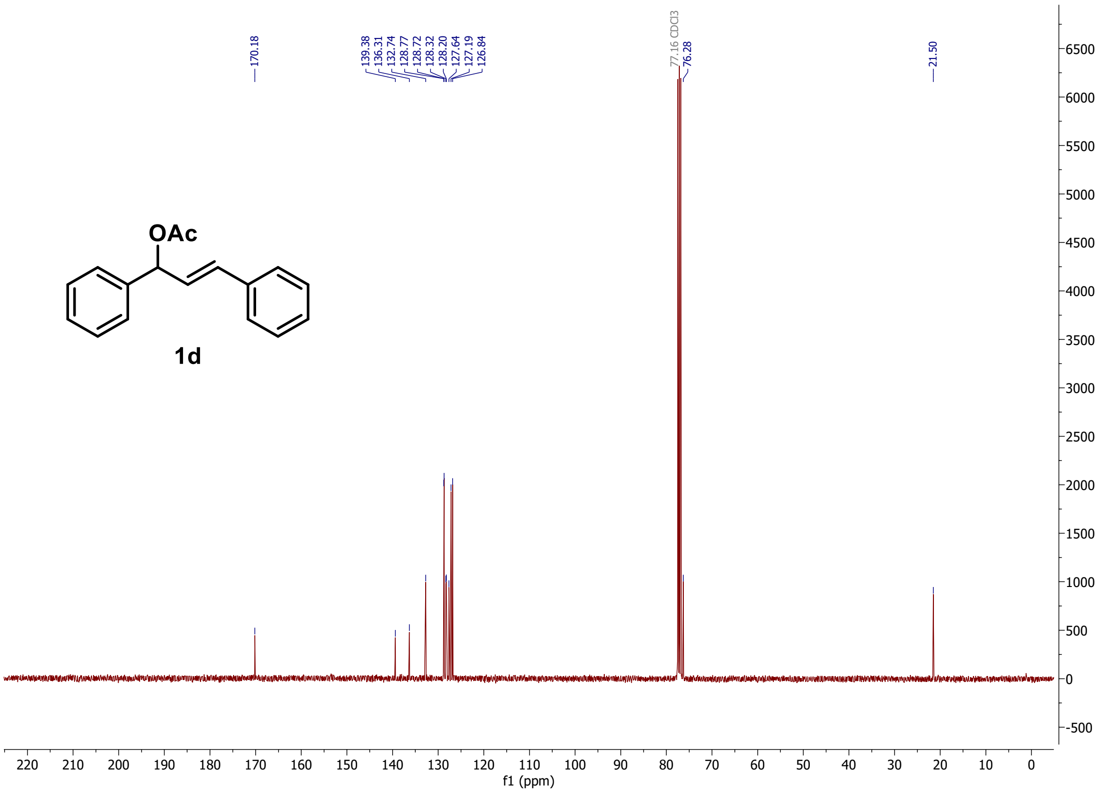

Figure S22. ^{13}C-NMR spectrum of **1d**

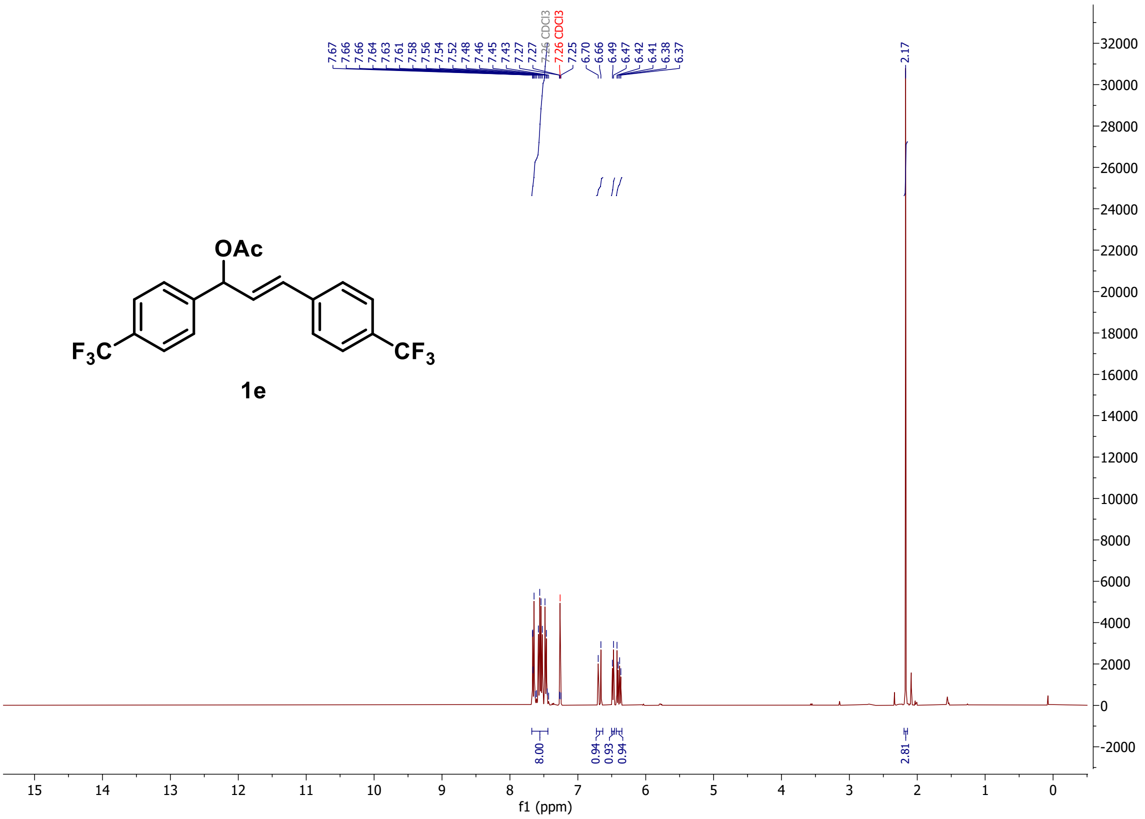

Figure S23. ^{1}H-NMR spectrum of **1e**

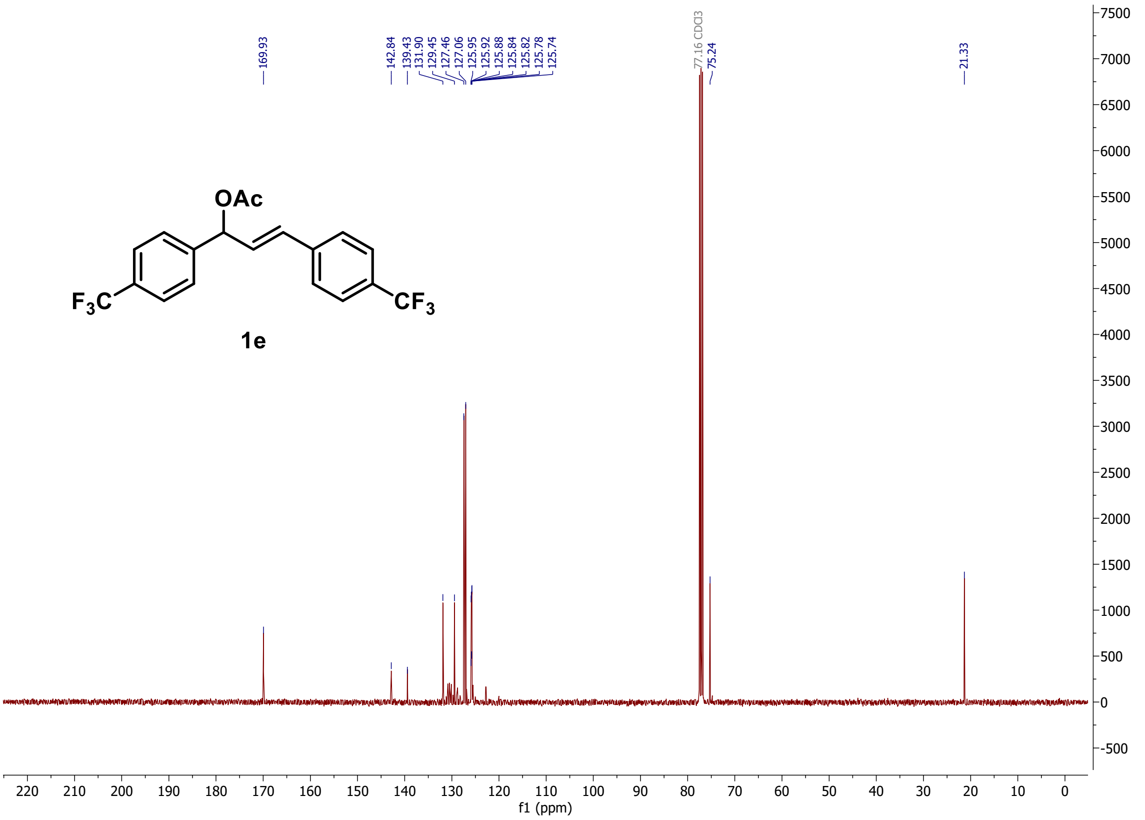

Figure S24. ^{13}C-NMR spectrum of **1e**

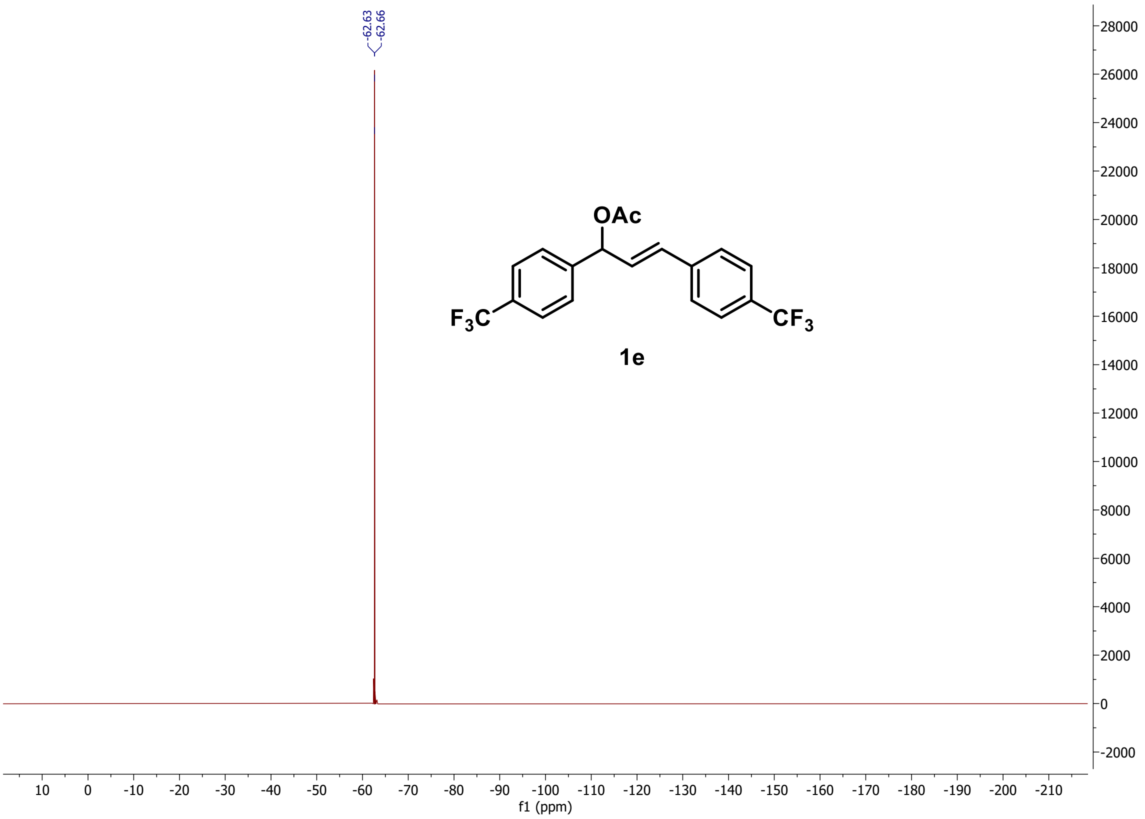

Figure S25. ^{19}F-NMR spectrum of **1e**

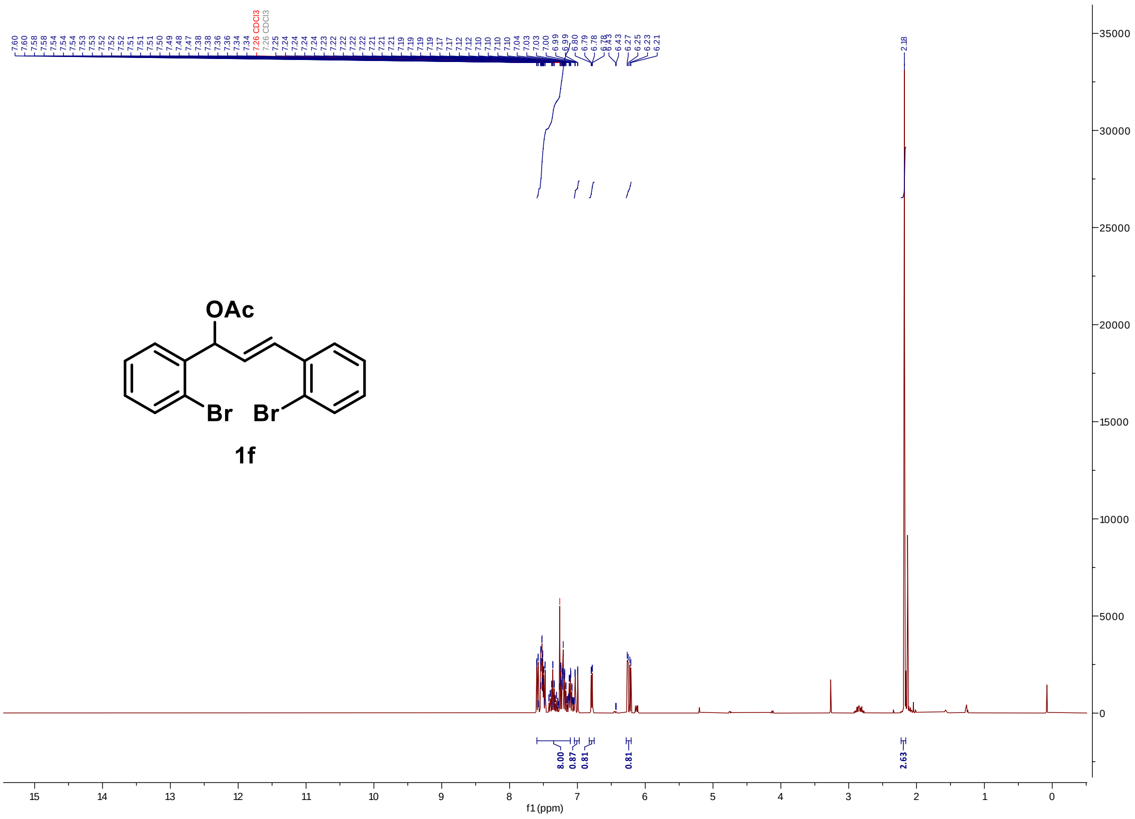

Figure S26. ^{1}H-NMR spectrum of **1f**

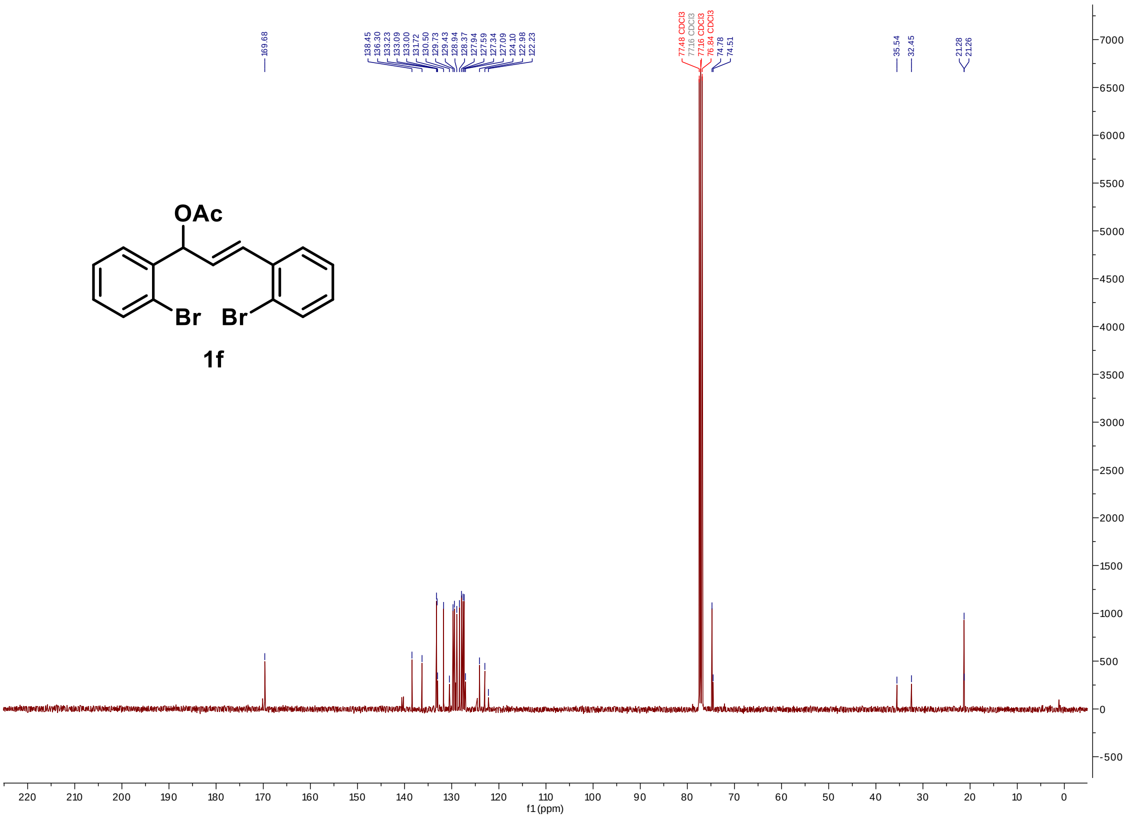

Figure S27. ^{13}C-NMR spectrum of **1f**

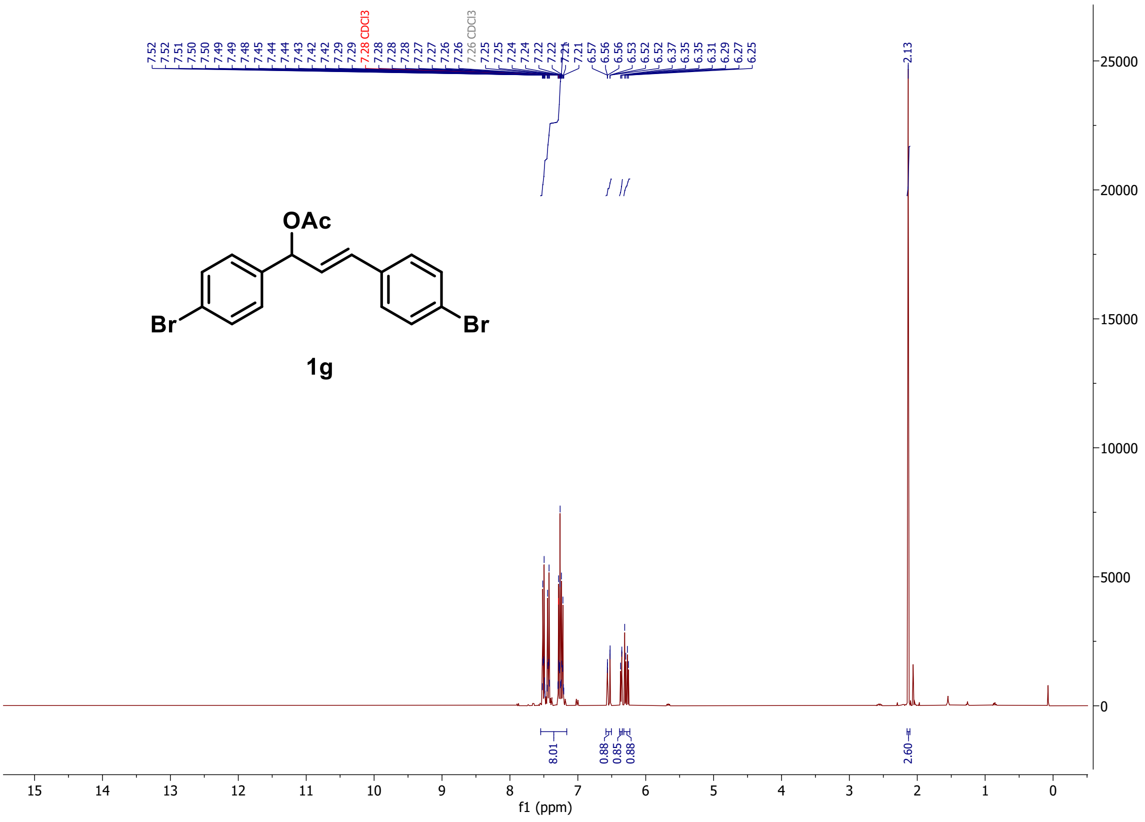

Figure S28. ^{1}H-NMR spectrum of **1g**

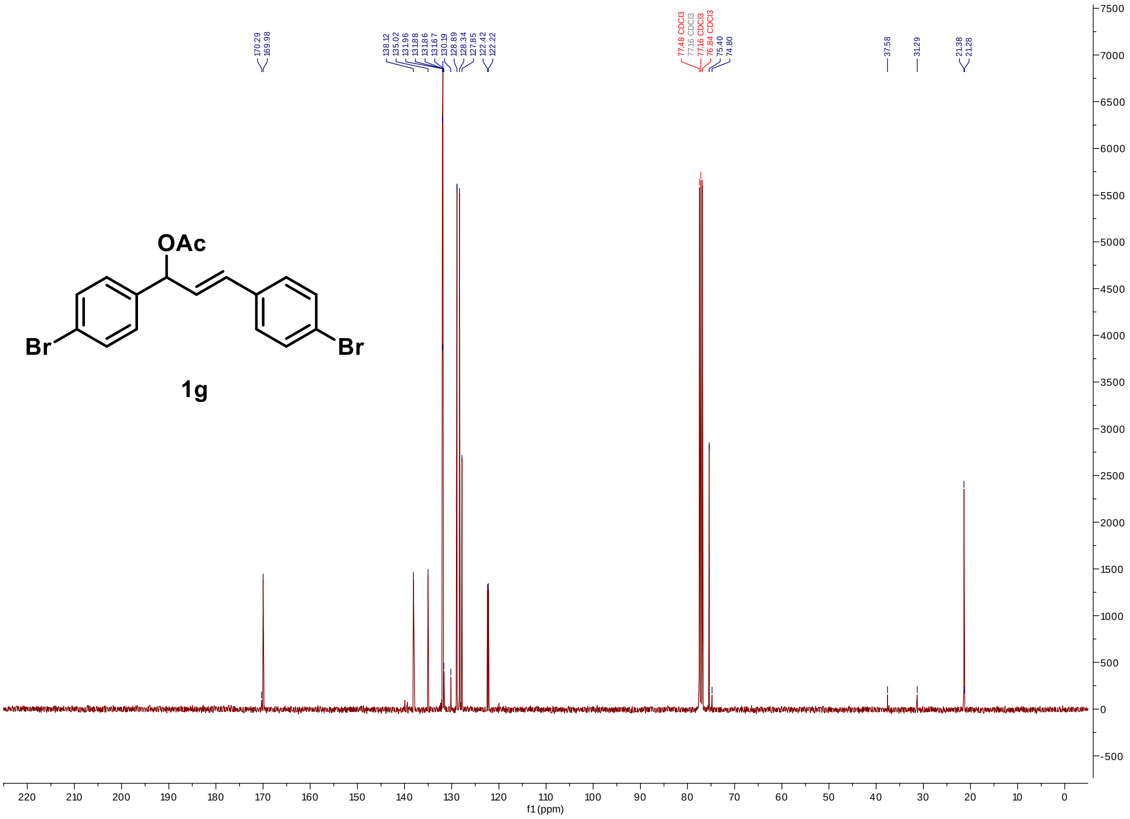

Figure S29. ^{13}C-NMR spectrum of **1g**

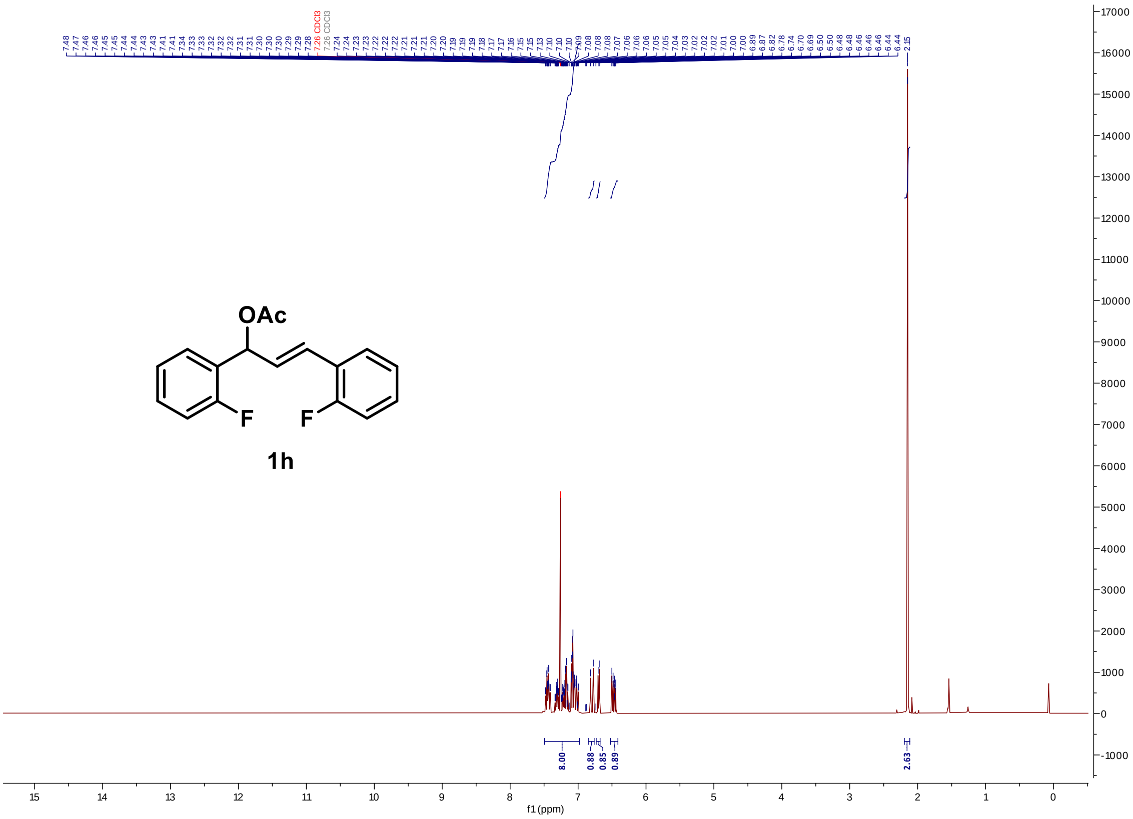

Figure S30. ^{1}H-NMR spectrum of **1h**

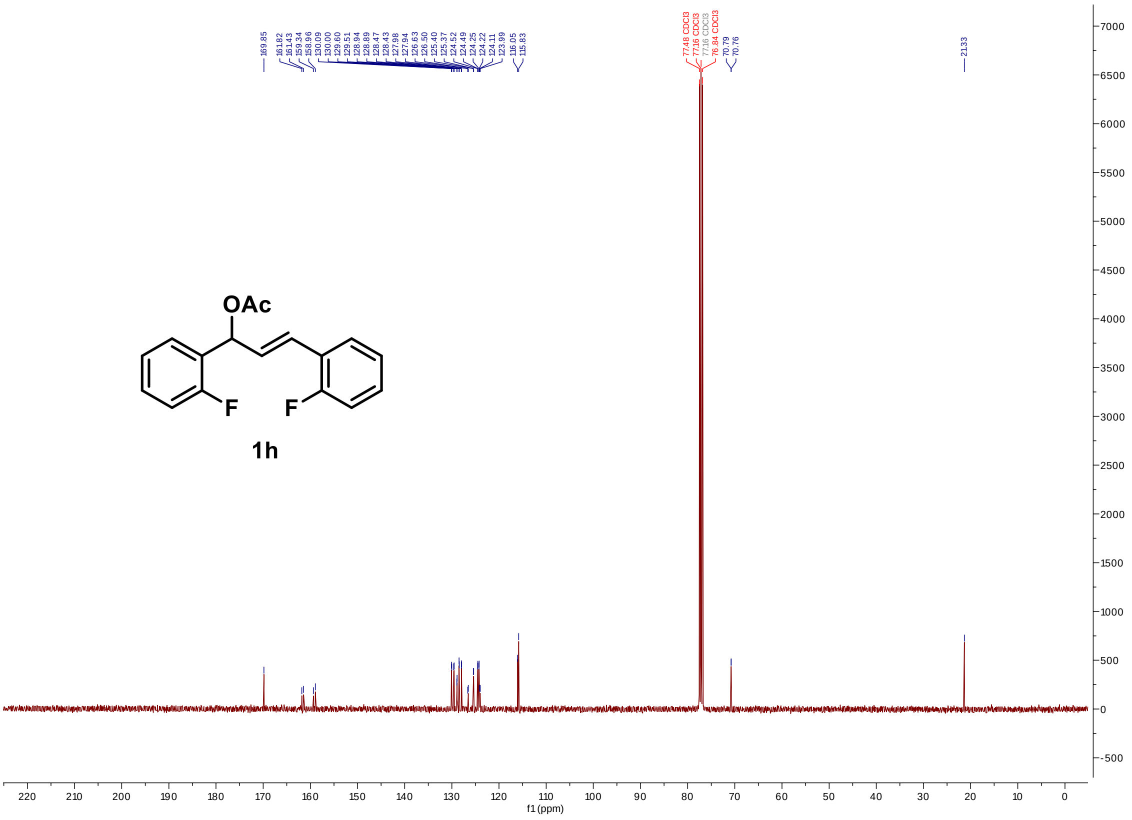

Figure S31. ^{13}C-NMR spectrum of **1h**

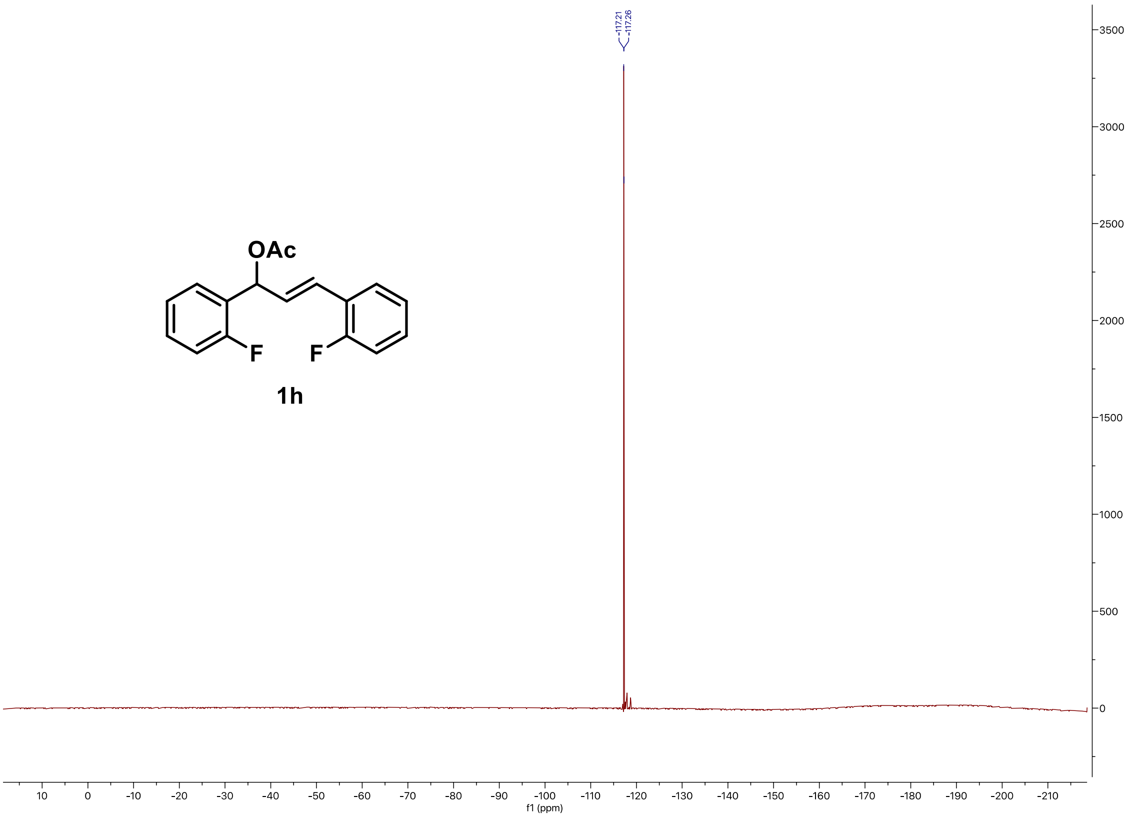

Figure S32. ^{19}F-NMR spectrum of **1h**

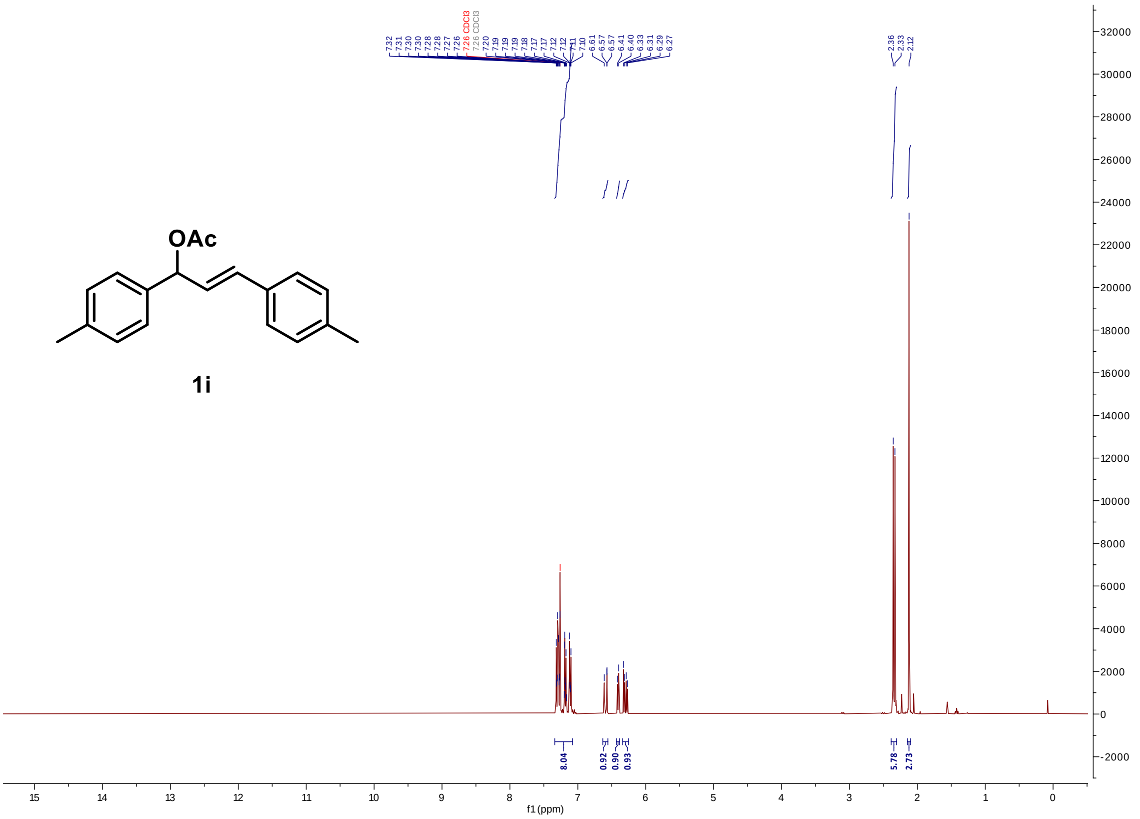

Figure S33. ^{1}H-NMR spectrum of **1i**

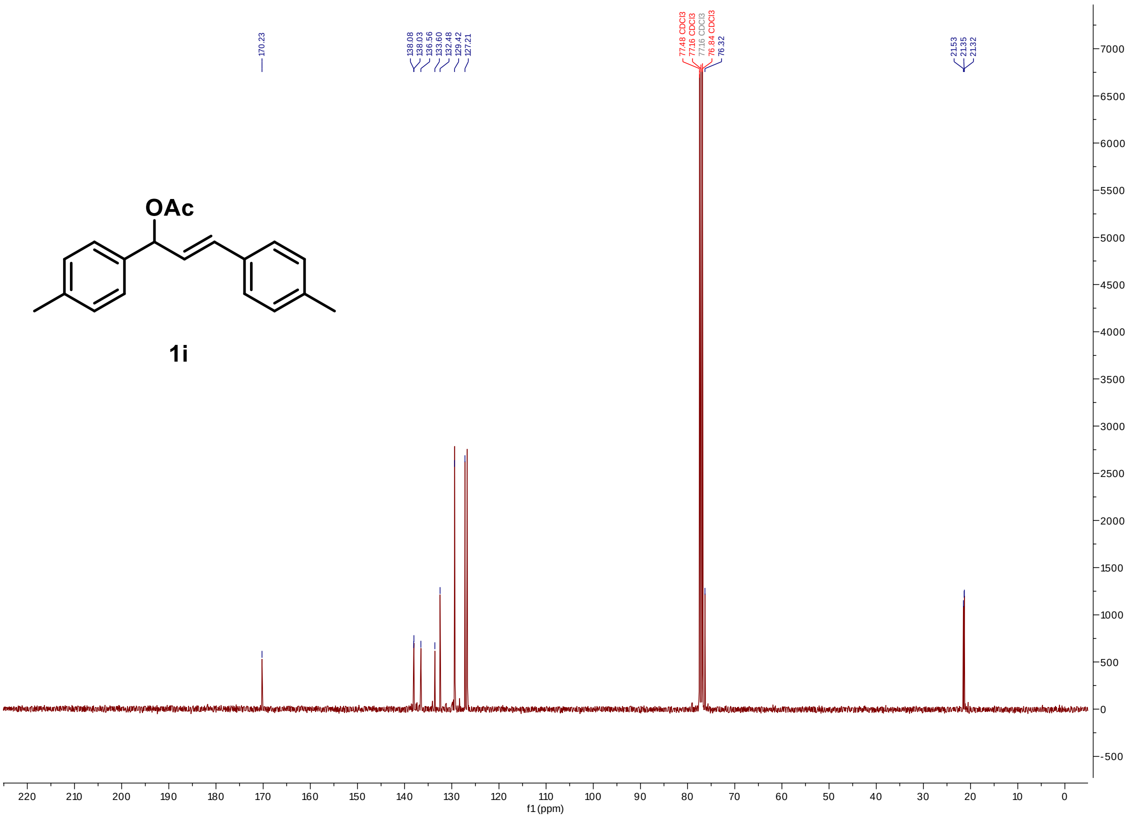

Figure S34. ^{13}C-NMR spectrum of **1i**

10. NMR spectra of amines 3a-h

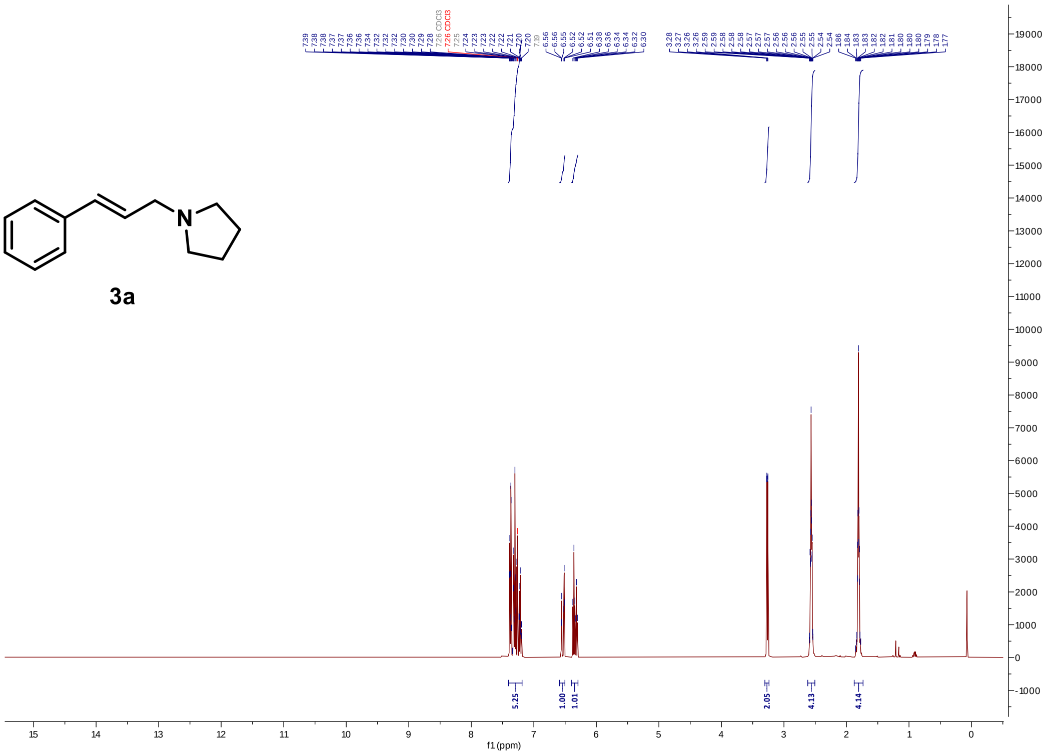

Figure S35. ^{1}H-NMR spectrum of **3a**

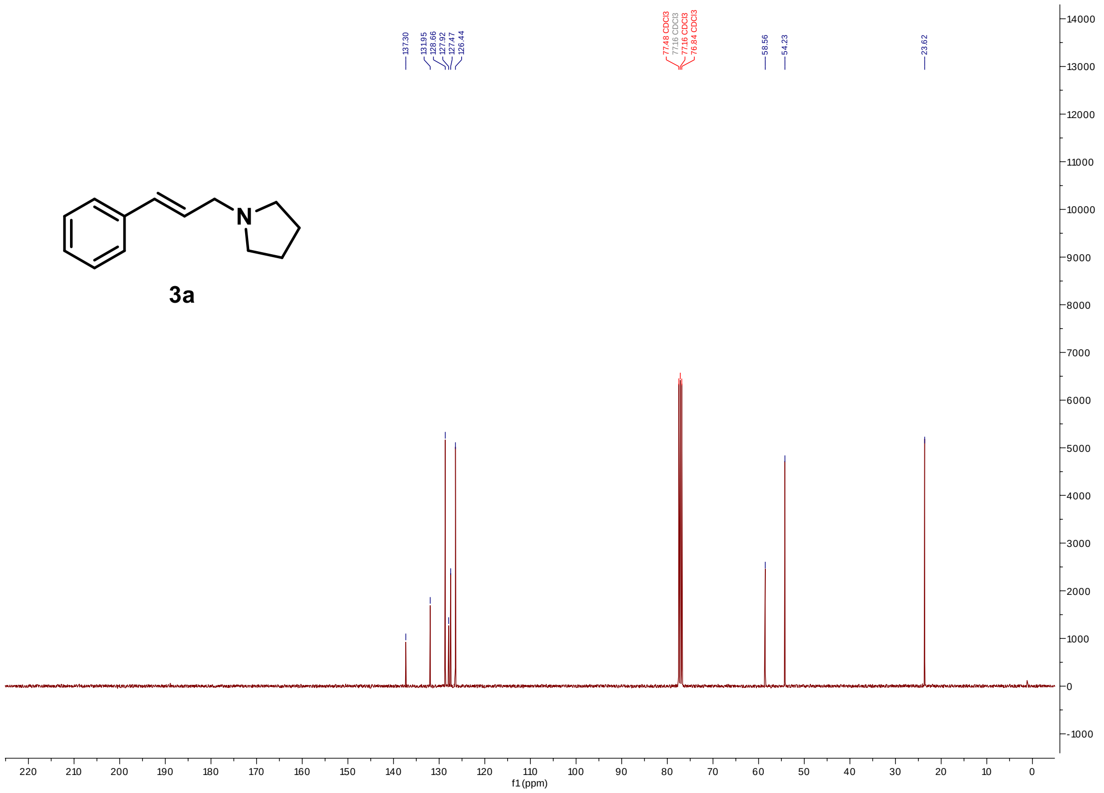

Figure S36. ^{13}C-NMR spectrum of **3a**

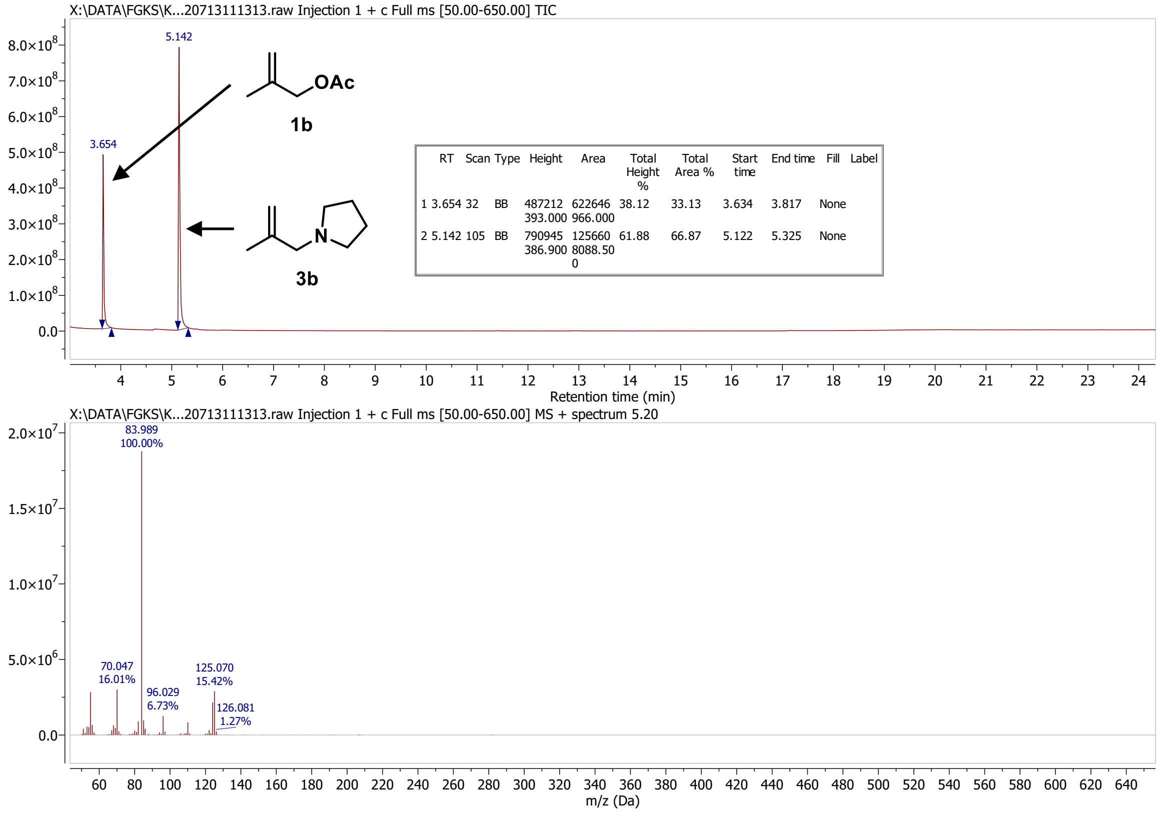

	RT	Scan	Type	Height	Area	Total Height %	Total Area %	Start time	End time	Fill	Label
1	3.654	32	BB	487212393.000	622646966.000	38.12	33.13	3.634	3.817	None	
2	5.142	105	BB	790945386.900	1256608088.500	61.88	66.87	5.122	5.325	None	

Figure S37. GC-MS chromatogram of **3b**

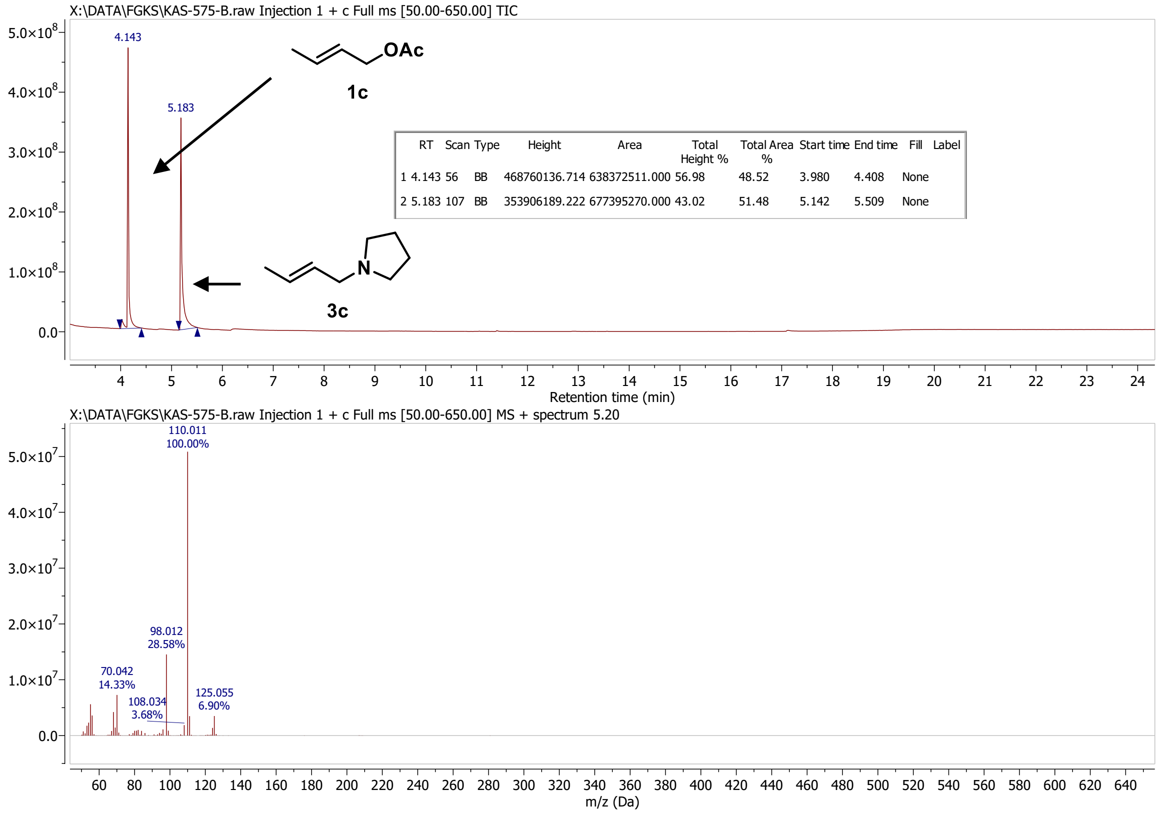

	RT	Scan	Type	Height	Area	Total Height %	Total Area %	Start time	End time	Fill	Label
1	4.143	56	BB	468760136.714	638372511.000	56.98	48.52	3.980	4.408	None	
2	5.183	107	BB	353906189.222	677395270.000	43.02	51.48	5.142	5.509	None	

Figure S38. GC-MS chromatogram of **3c**

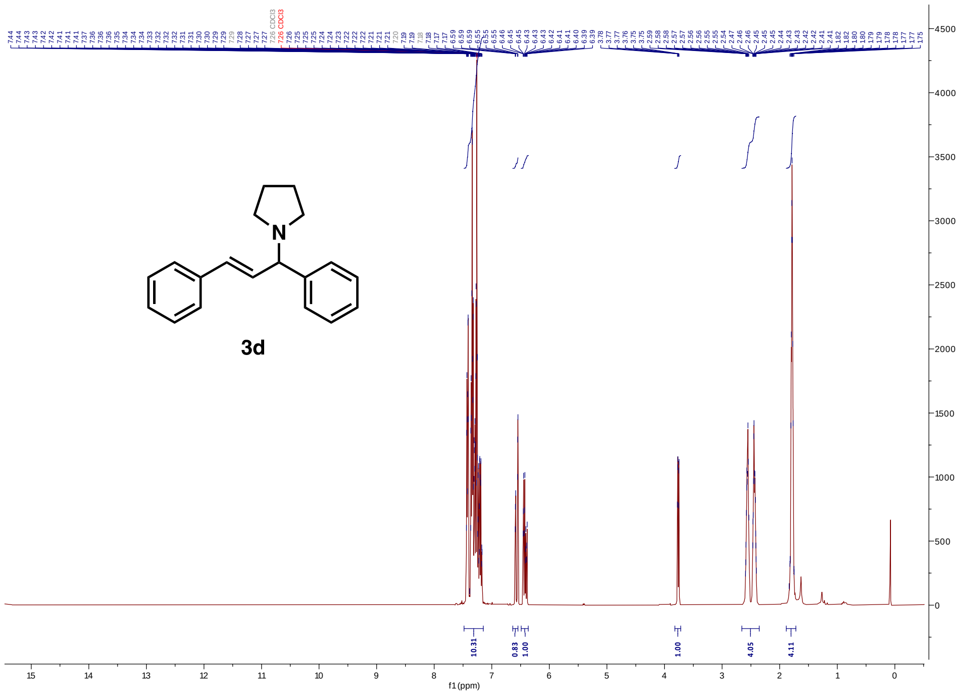

Figure S39. ^{1}H-NMR spectrum of **3d**

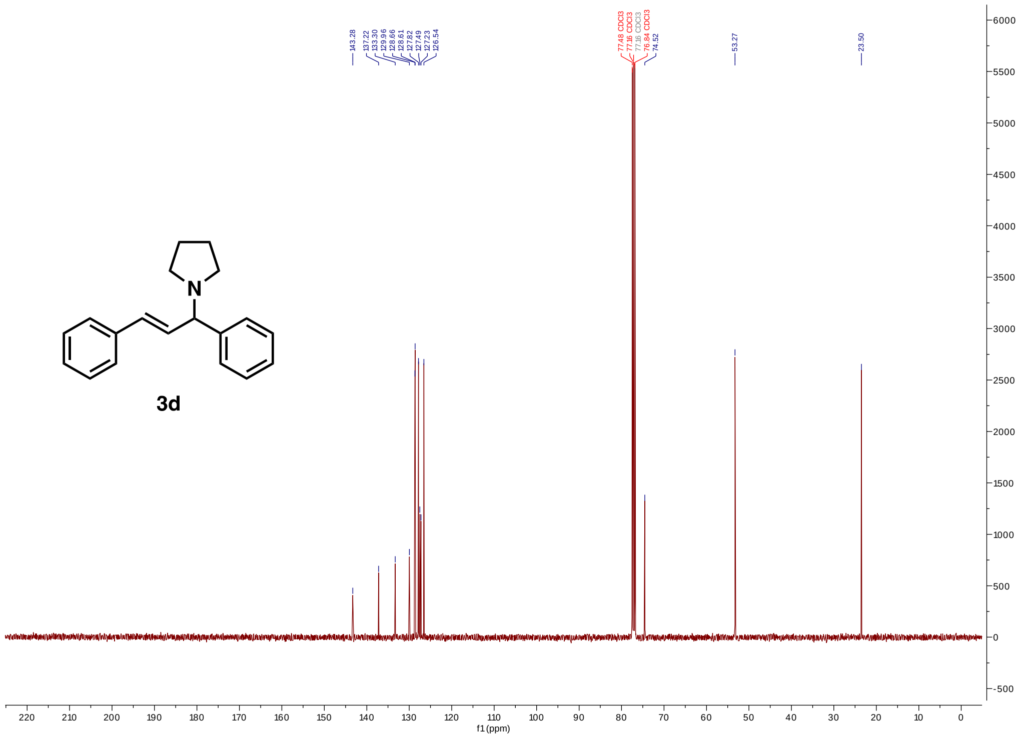

Figure S40. ^{13}C-NMR spectrum of **3d**

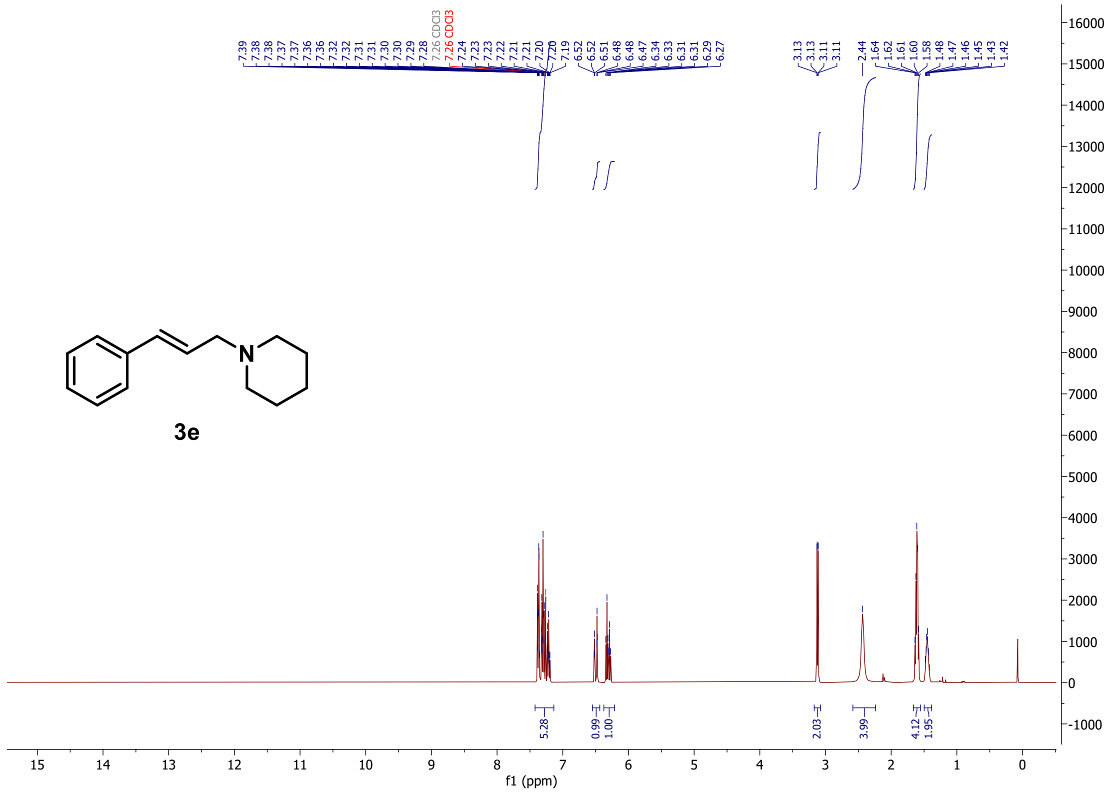

Figure S41. ^{1}H-NMR spectrum of **3e**

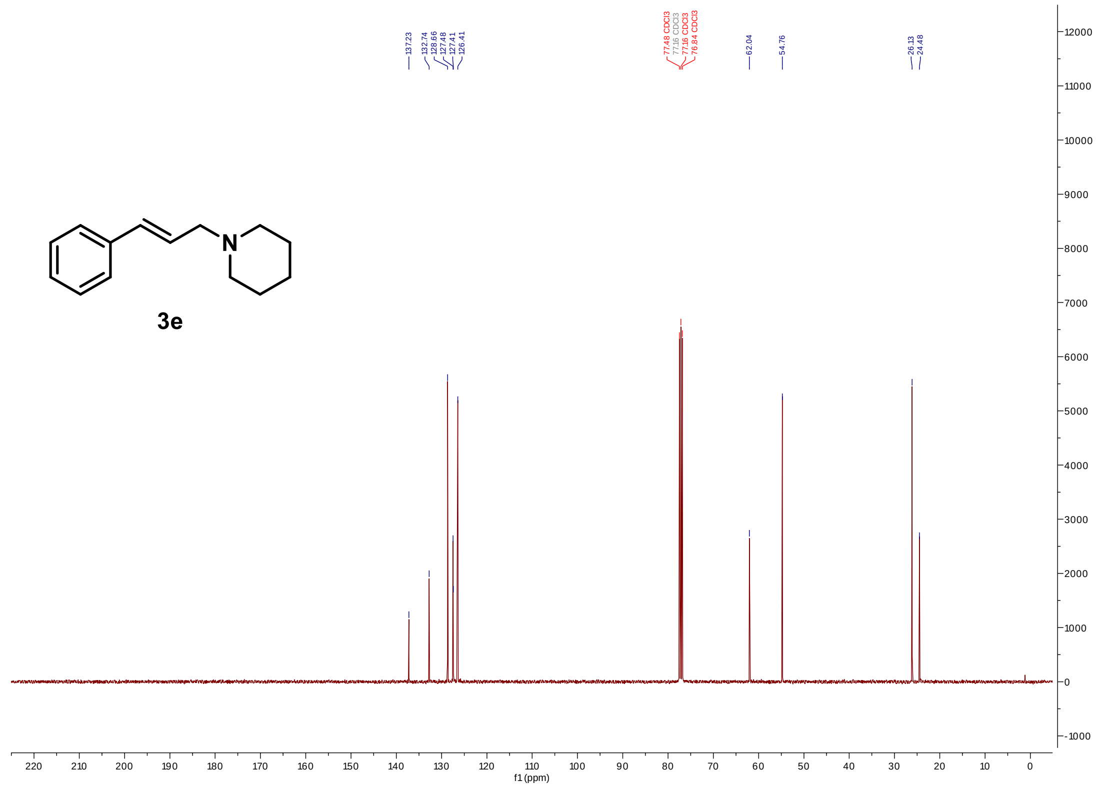

Figure S42. ^{13}C-NMR spectrum of **3e**

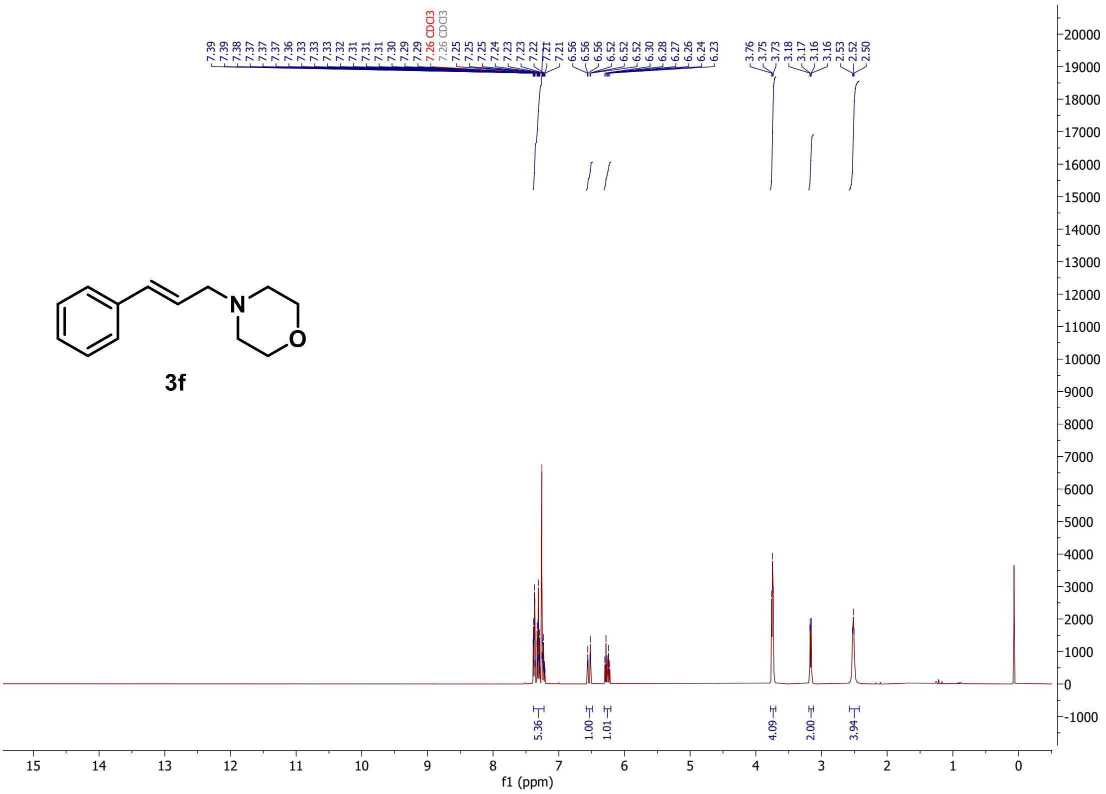

Figure S43. ^{1}H-NMR spectrum of **3f**

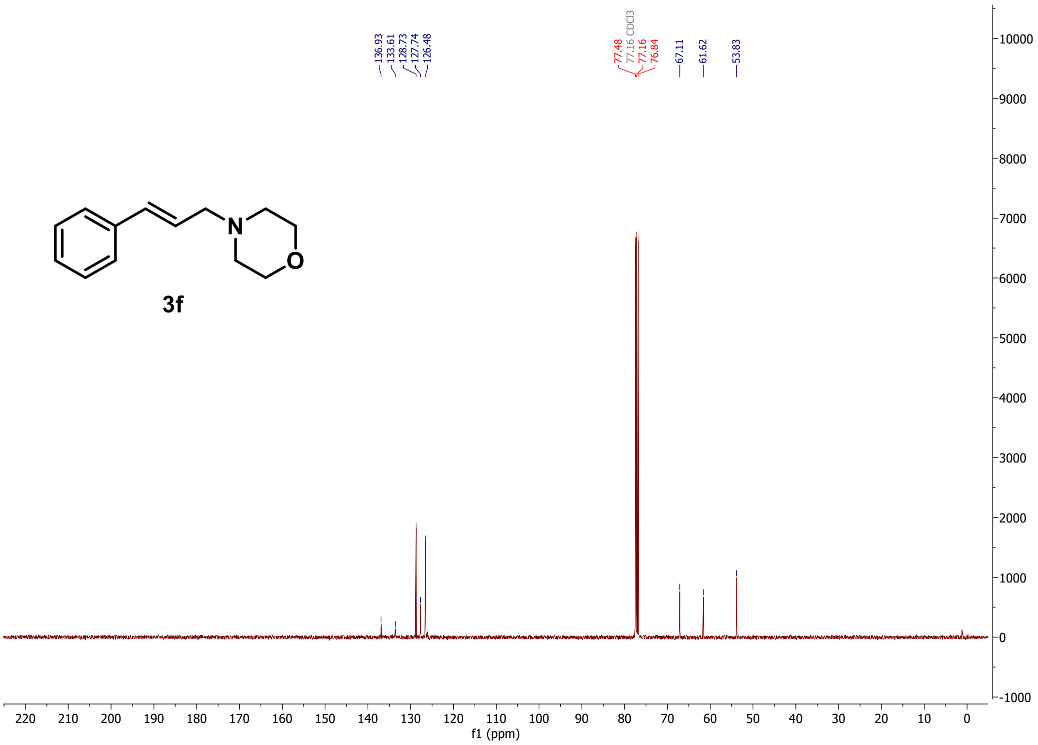

Figure S44. ^{13}C-NMR spectrum of **3f**

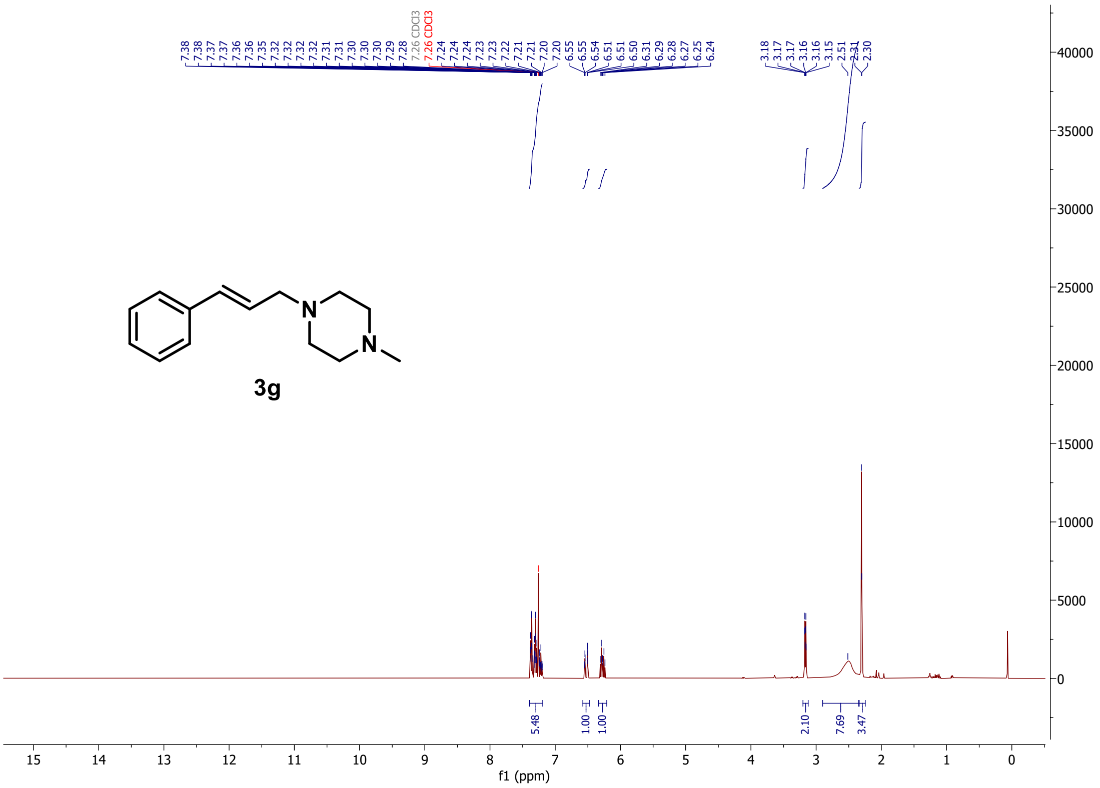

Figure S45. ^{1}H-NMR spectrum of **3g**

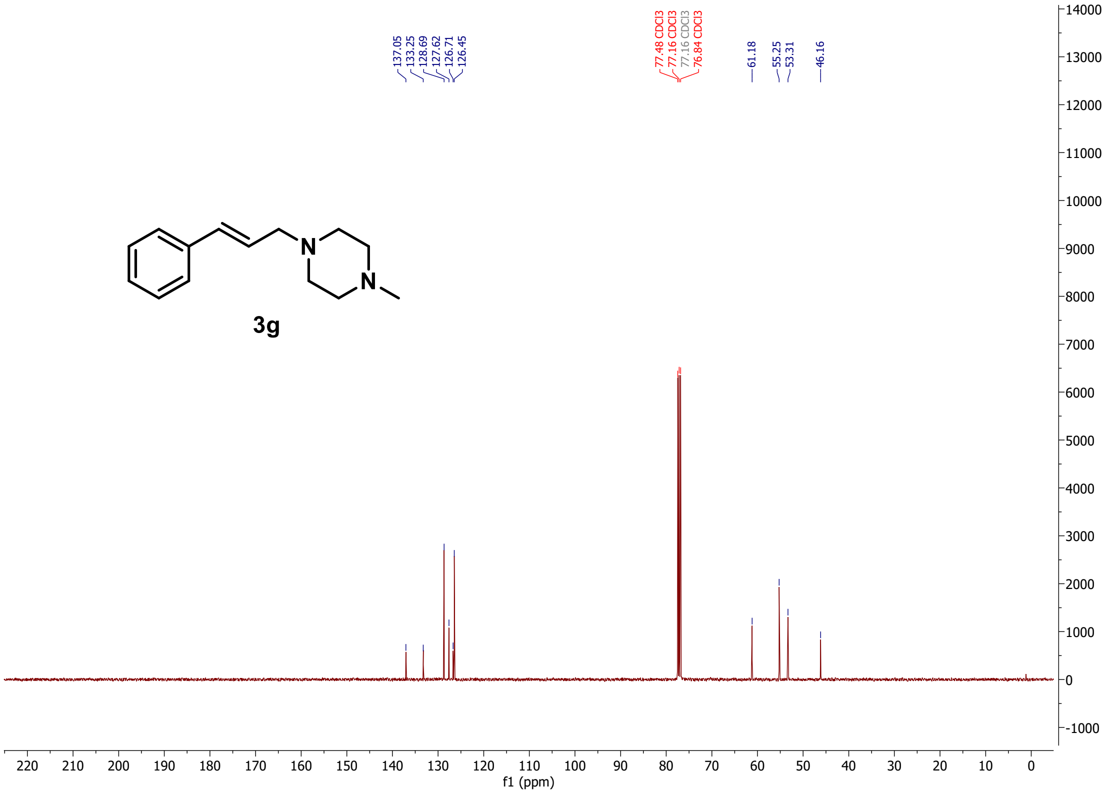

Figure S46. ^{13}C-NMR spectrum of **3g**

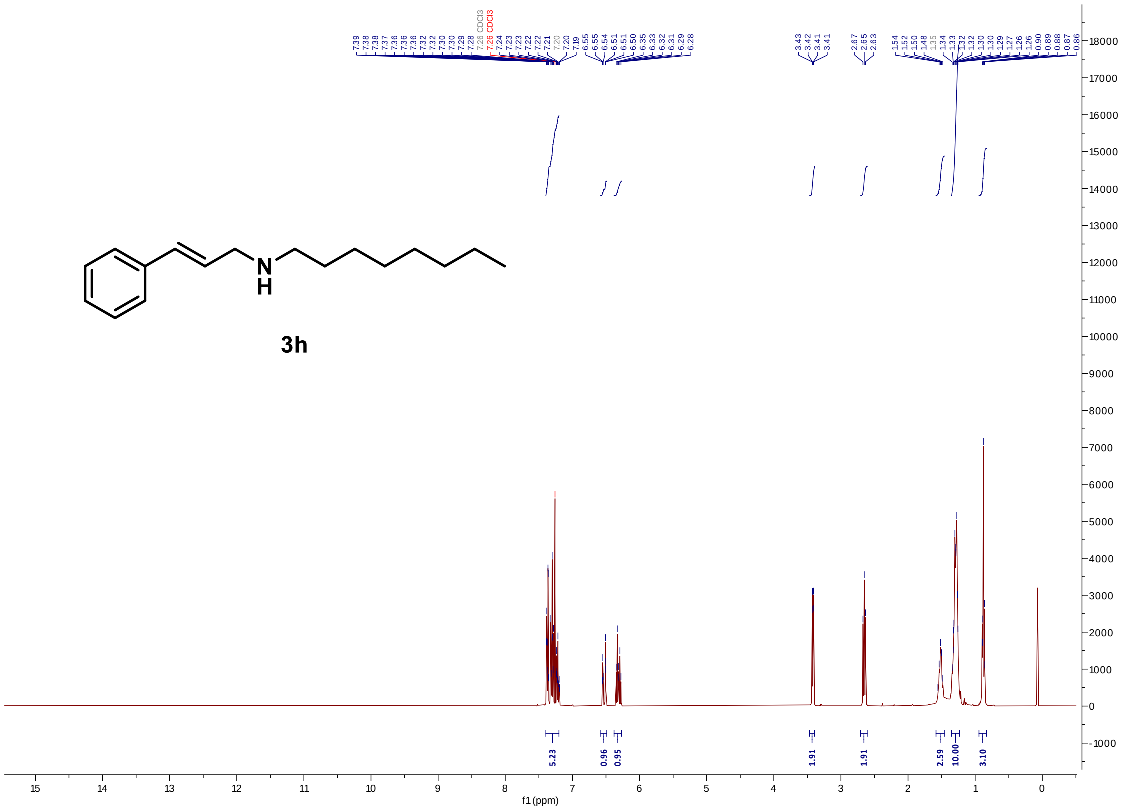

Figure S47. ^{1}H-NMR spectrum of **3h**

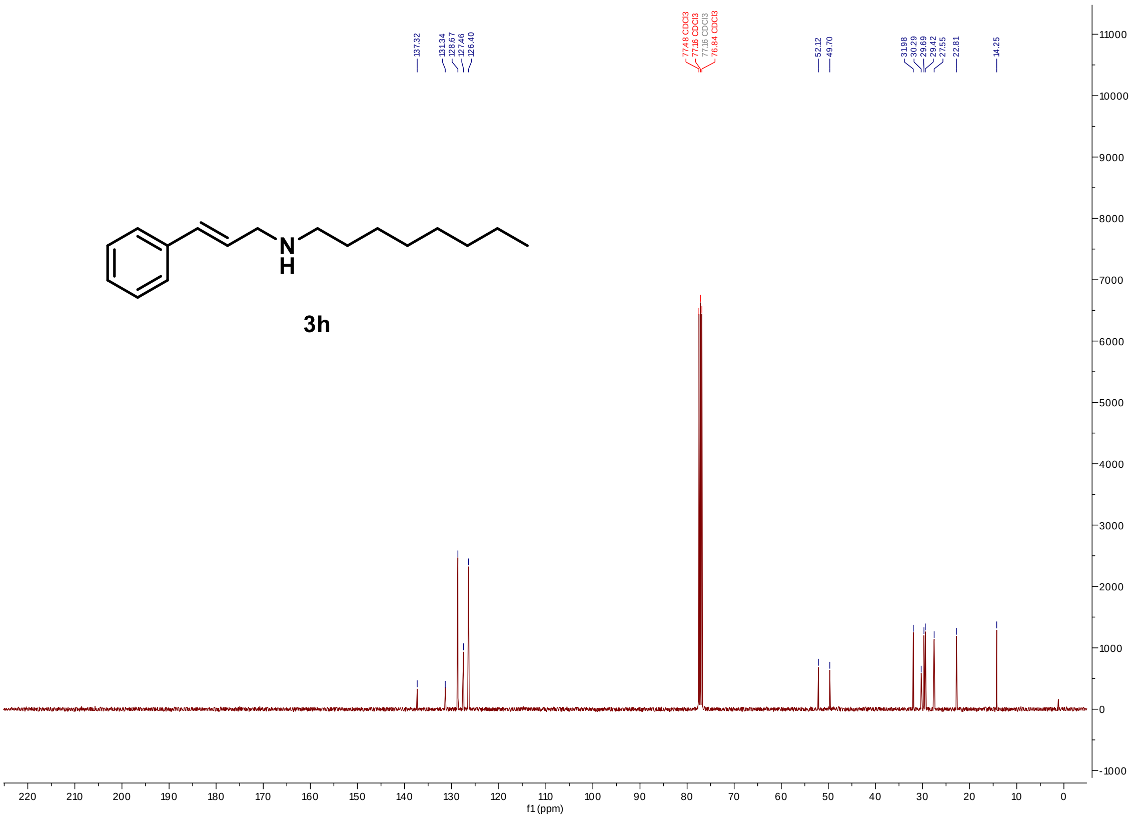

Figure S48. ^{13}C-NMR spectrum of **3h**

11. NMR spectra of chiral ligands

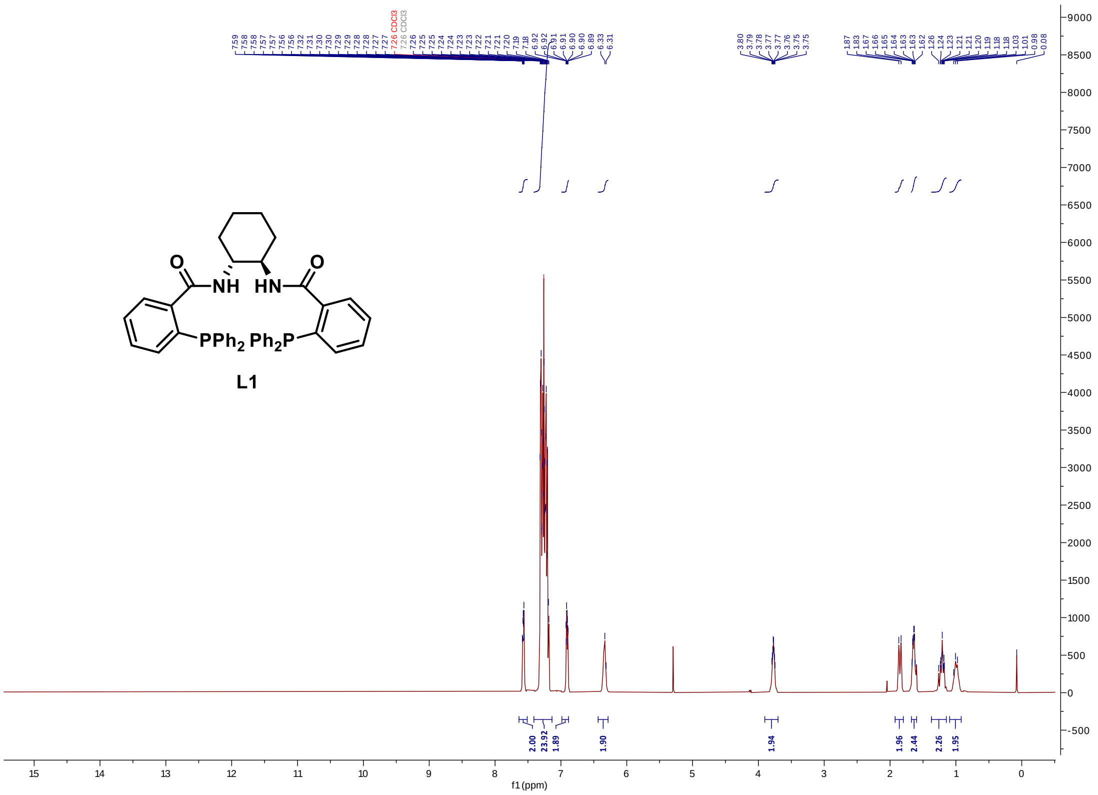

Figure S49. ^{1}H-NMR spectrum of **L1**

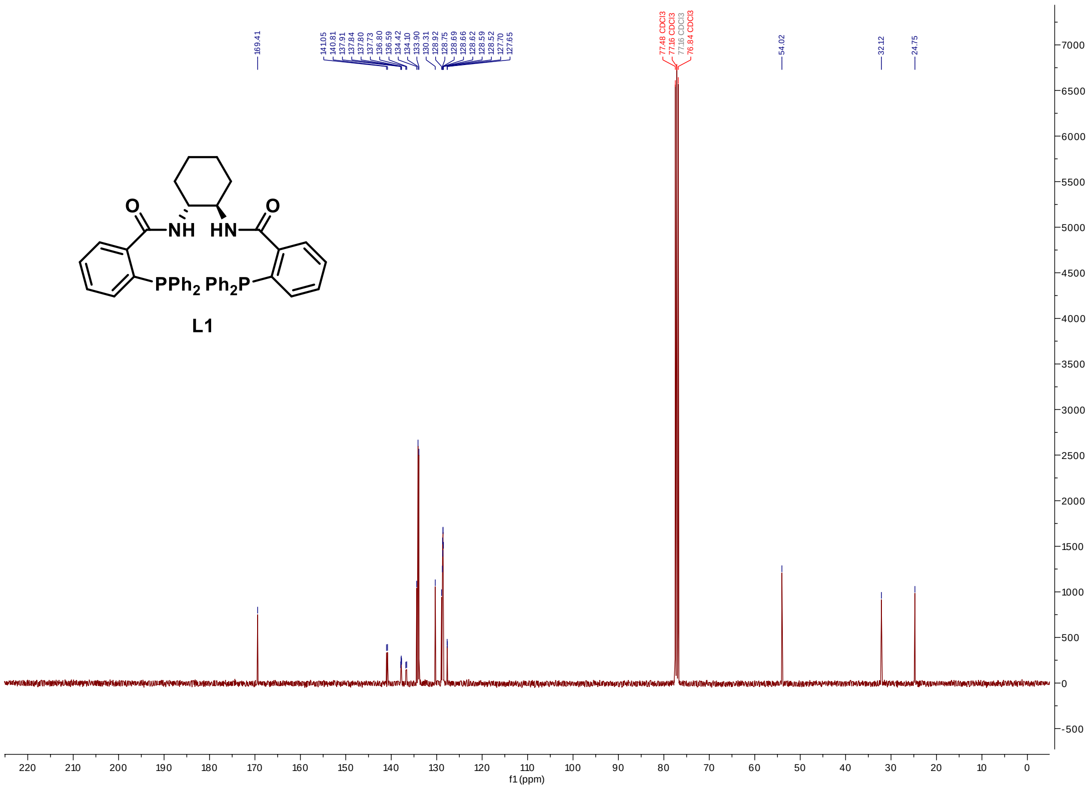

Figure S50. ^{13}C-NMR spectrum of **L1**

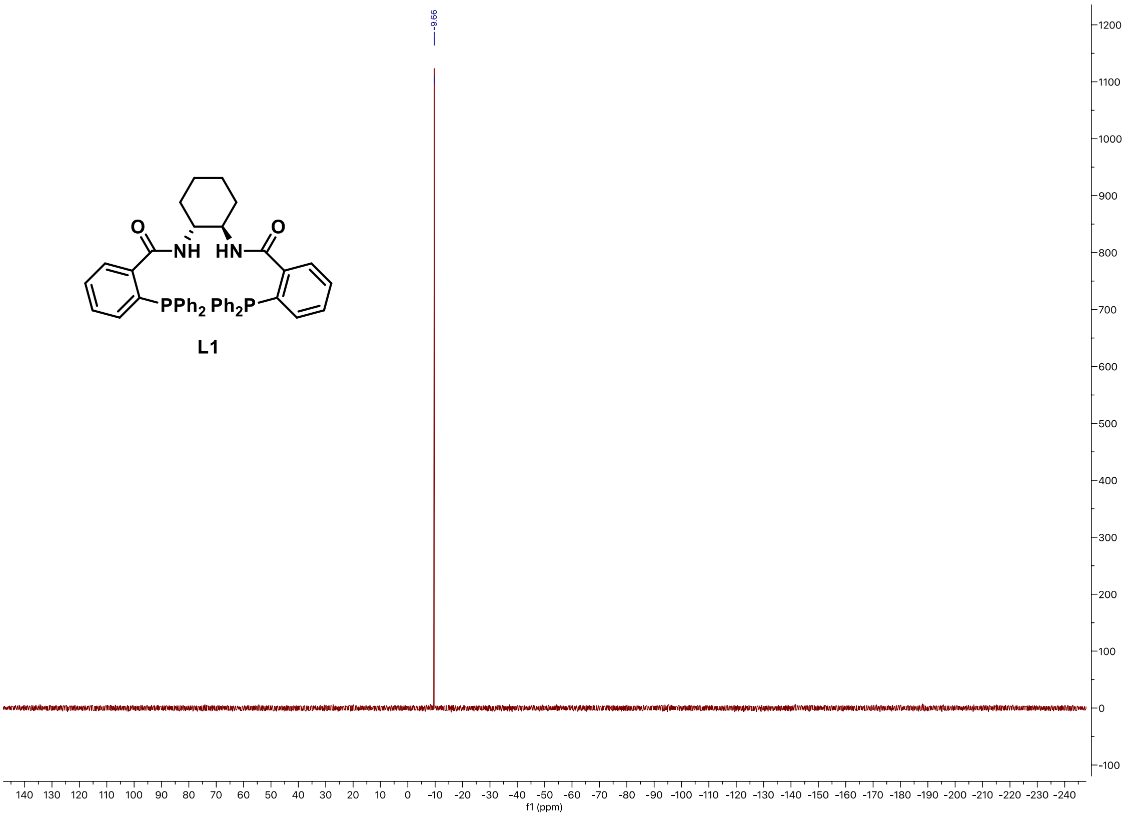

Figure S51. ^{31}P NMR of **L1**

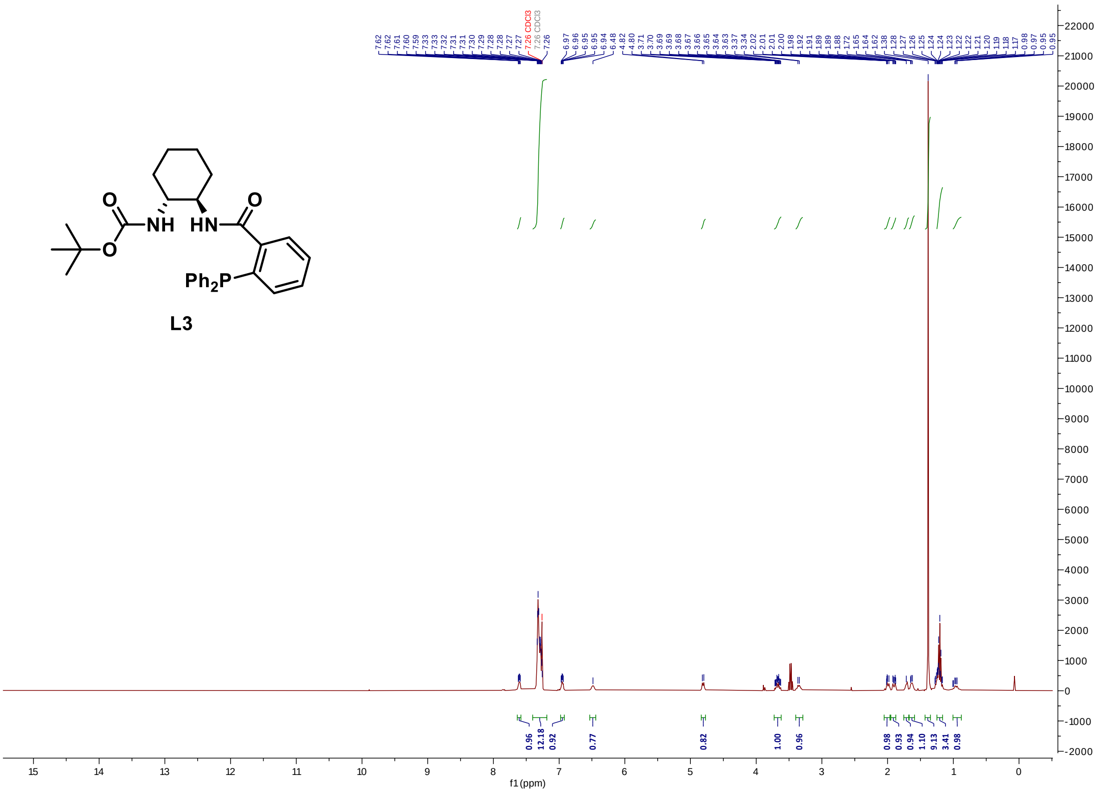

Figure S52. ^{1}H-NMR spectrum of **L3**

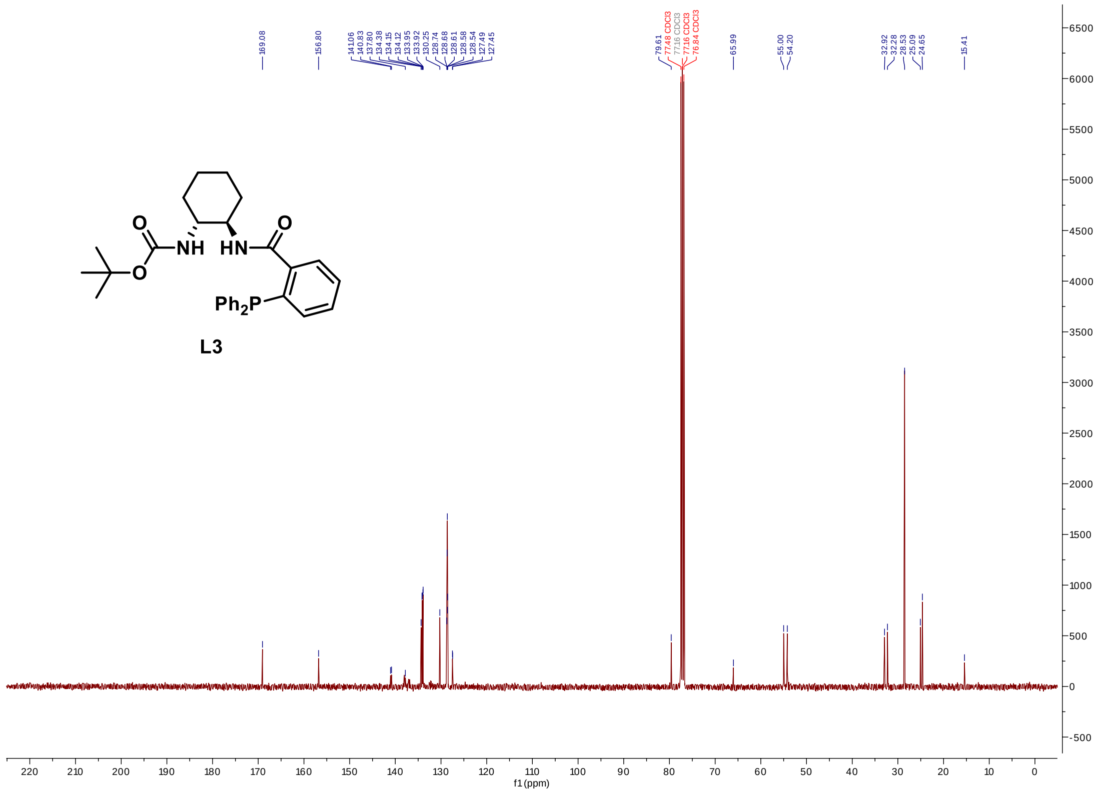

Figure S53. ^{13}C-NMR spectrum of **L3**

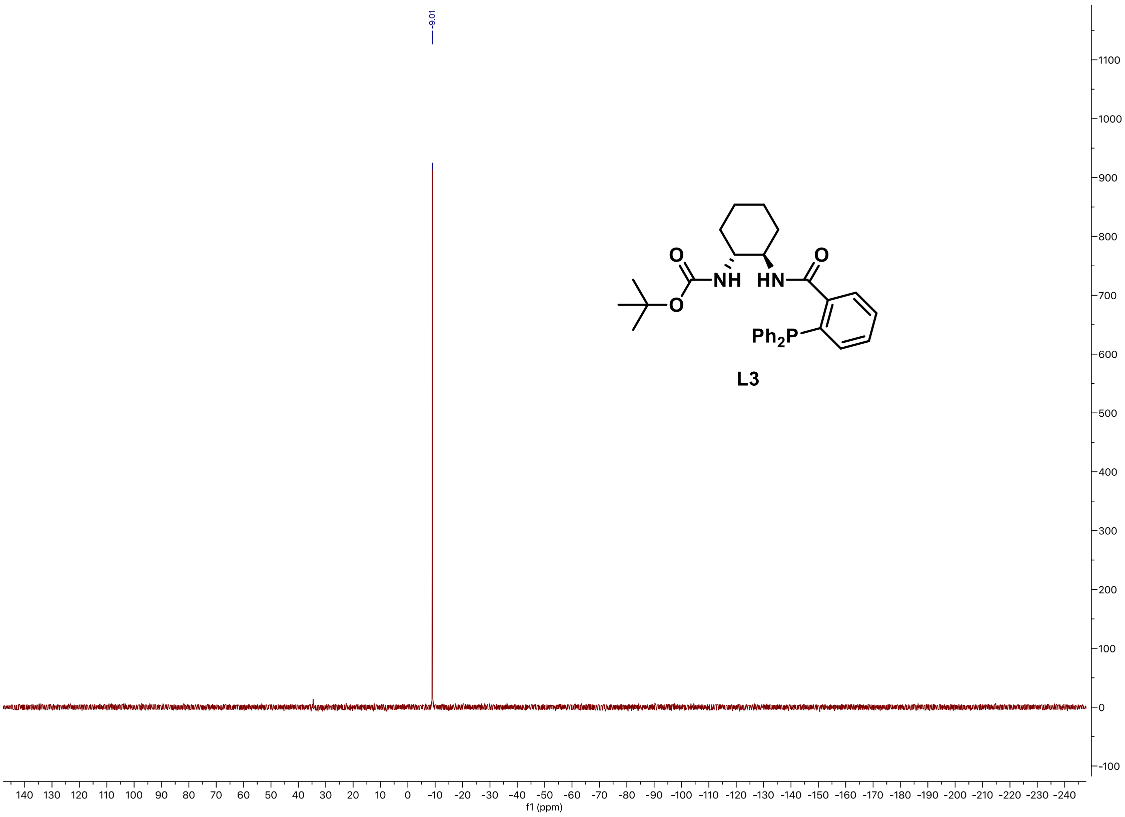

Figure S54. ^{31}P NMR of **L3**

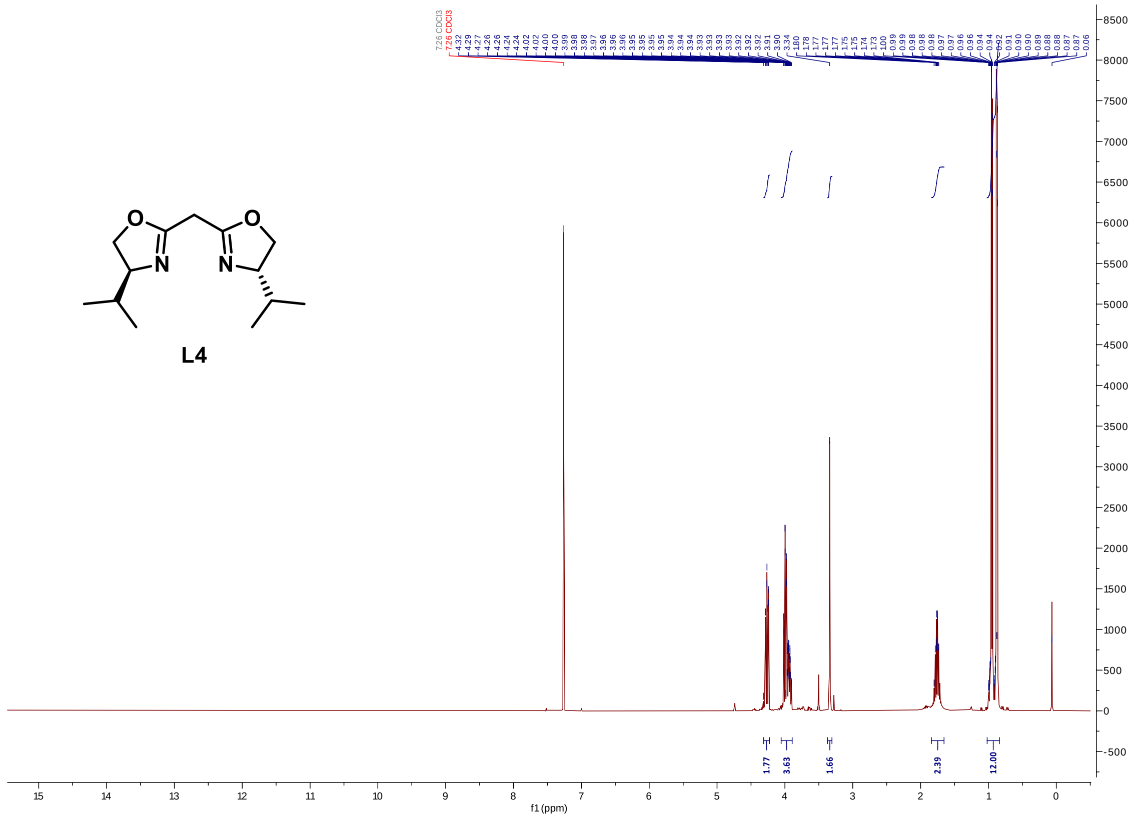

Figure S55. ^{1}H-NMR spectrum of **L4**

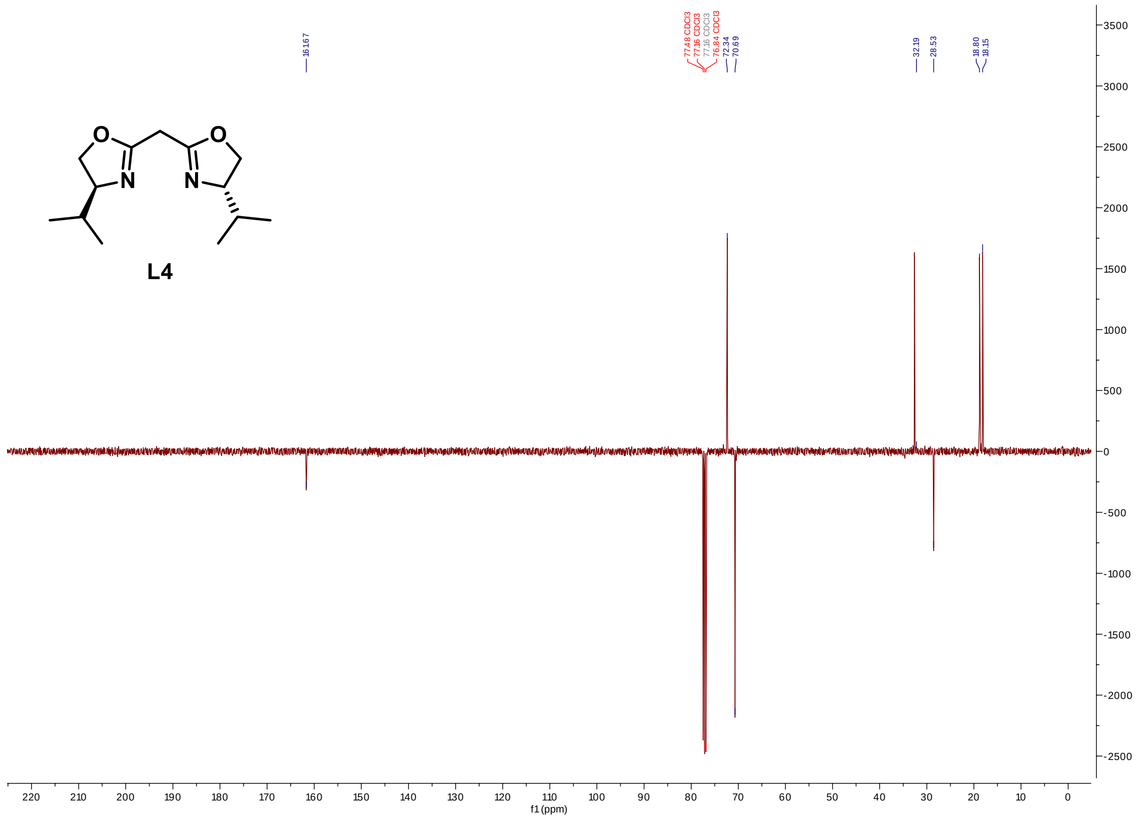

Figure S56. ^{13}C NMR of **L4** (APT)

12. NMR spectra of amines 3d, 3i-m

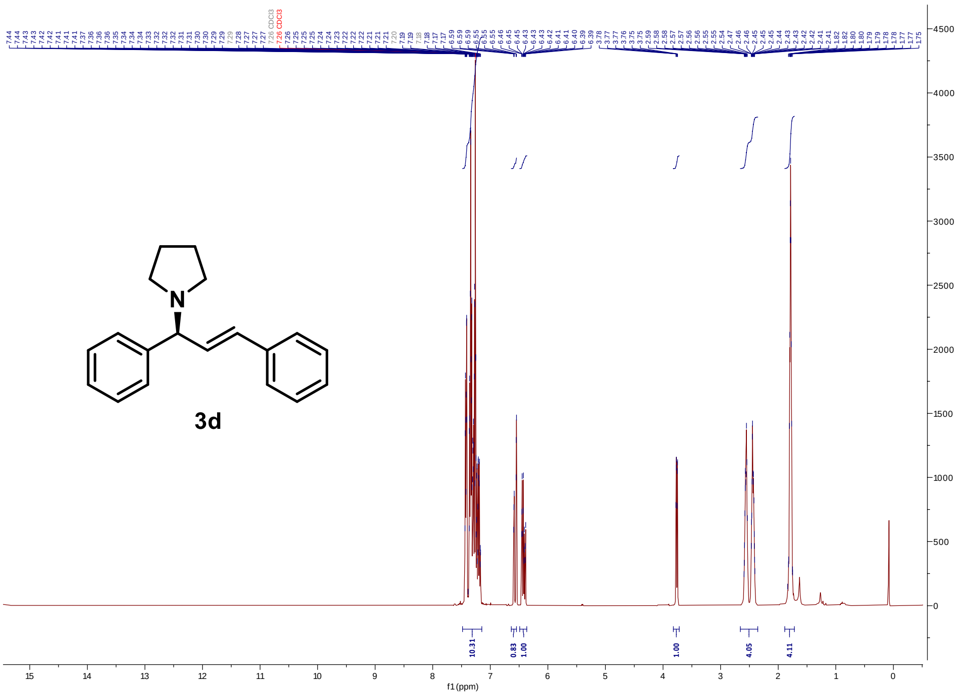

Figure S57. ^{1}H-NMR spectrum of **3d**

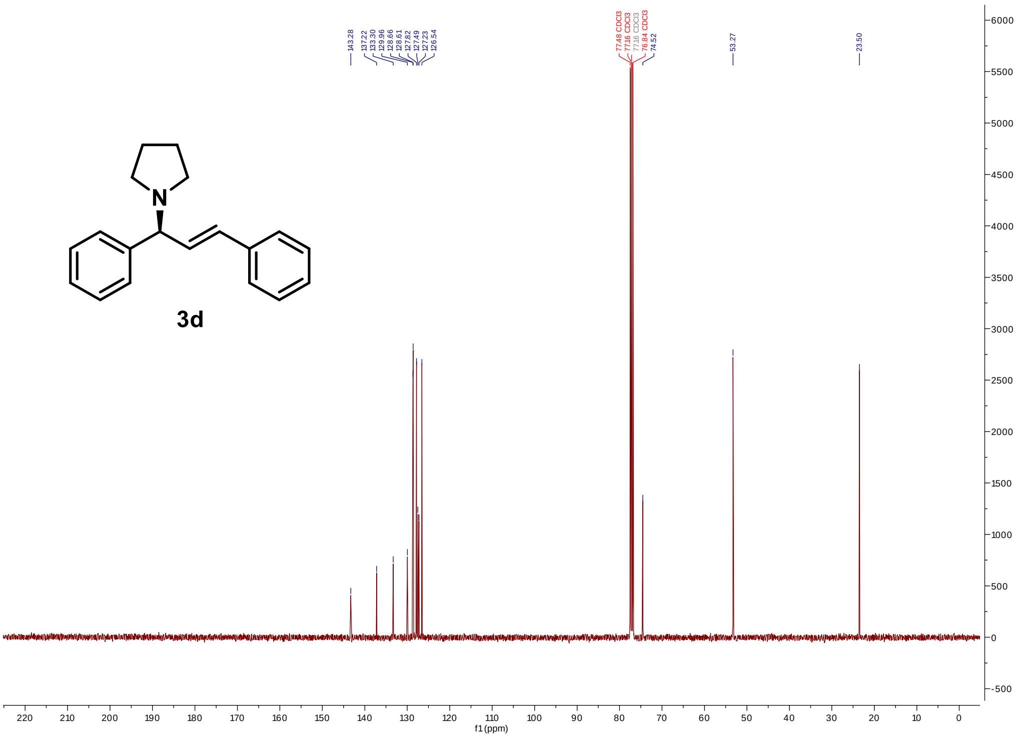

Figure S58. ^{13}C-NMR spectrum of **3d**

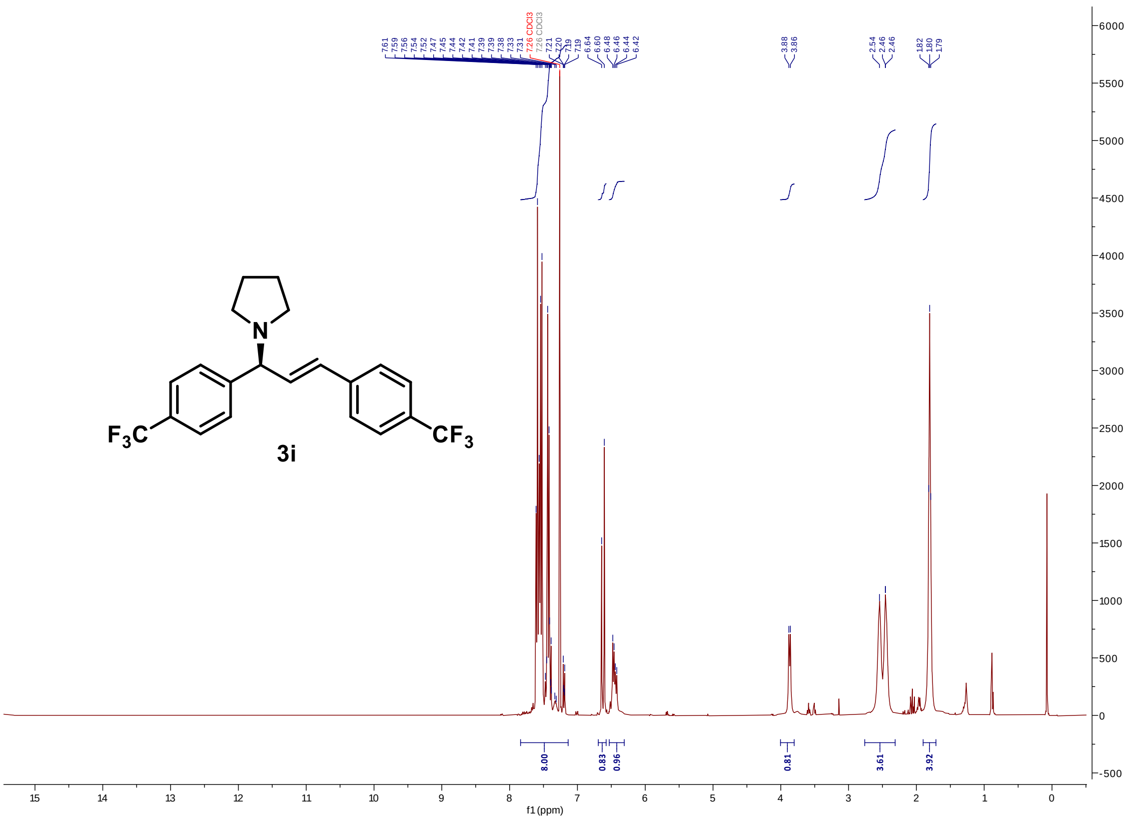

Figure S59. ^{1}H-NMR spectrum of **3i**

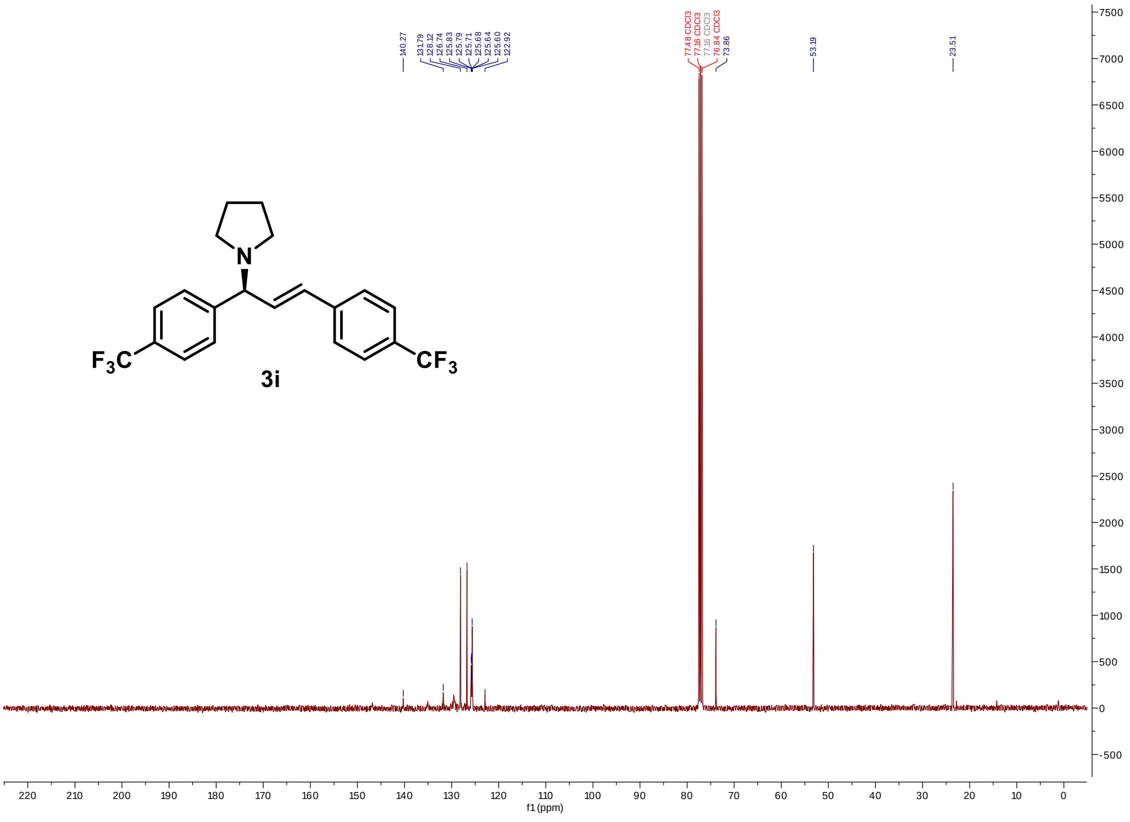

Figure S60. ^{13}C-NMR spectrum of **3i**

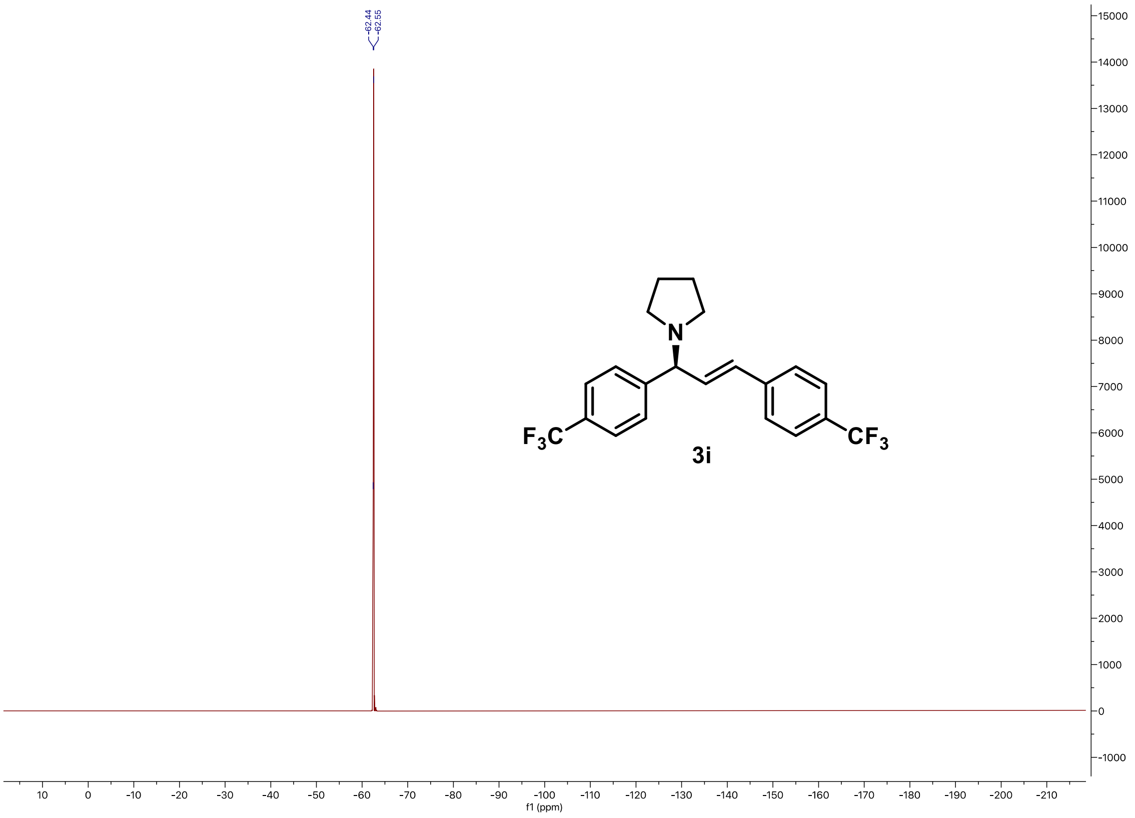

Figure S61. ^{19}F-NMR spectrum of **3i**

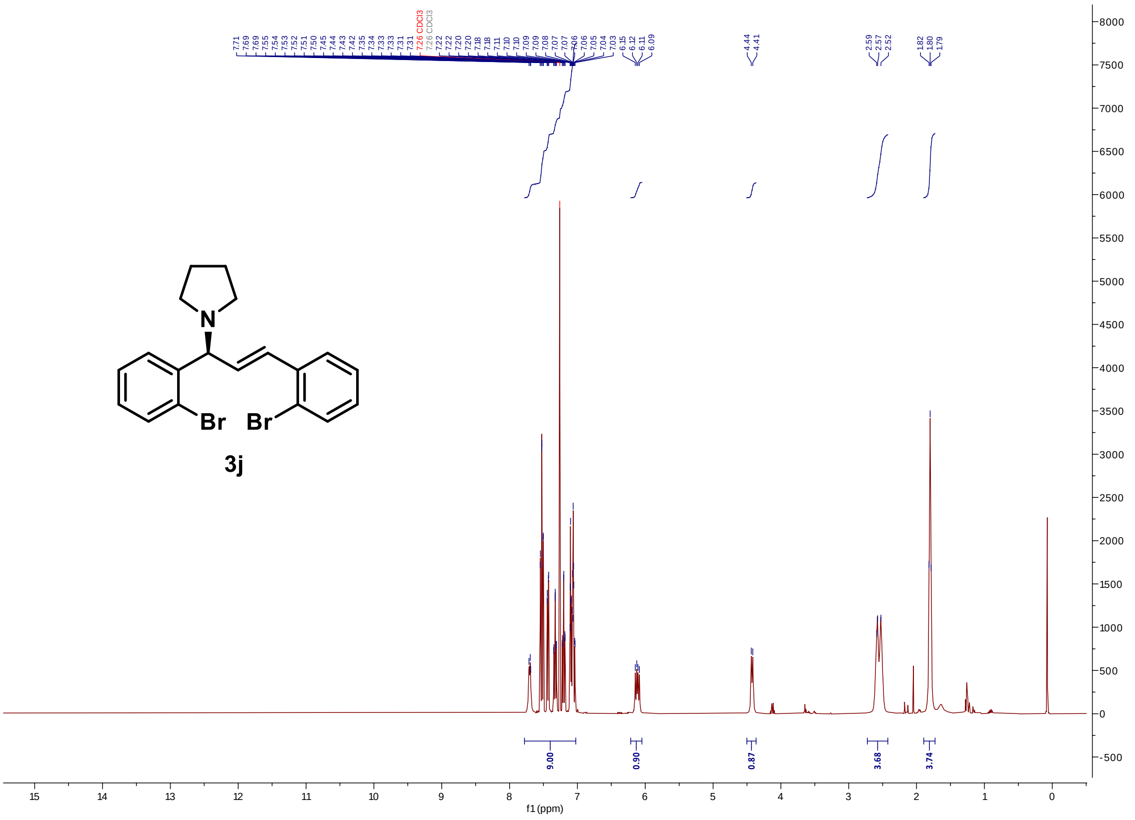

Figure S62. ^{1}H-NMR spectrum of **3j**

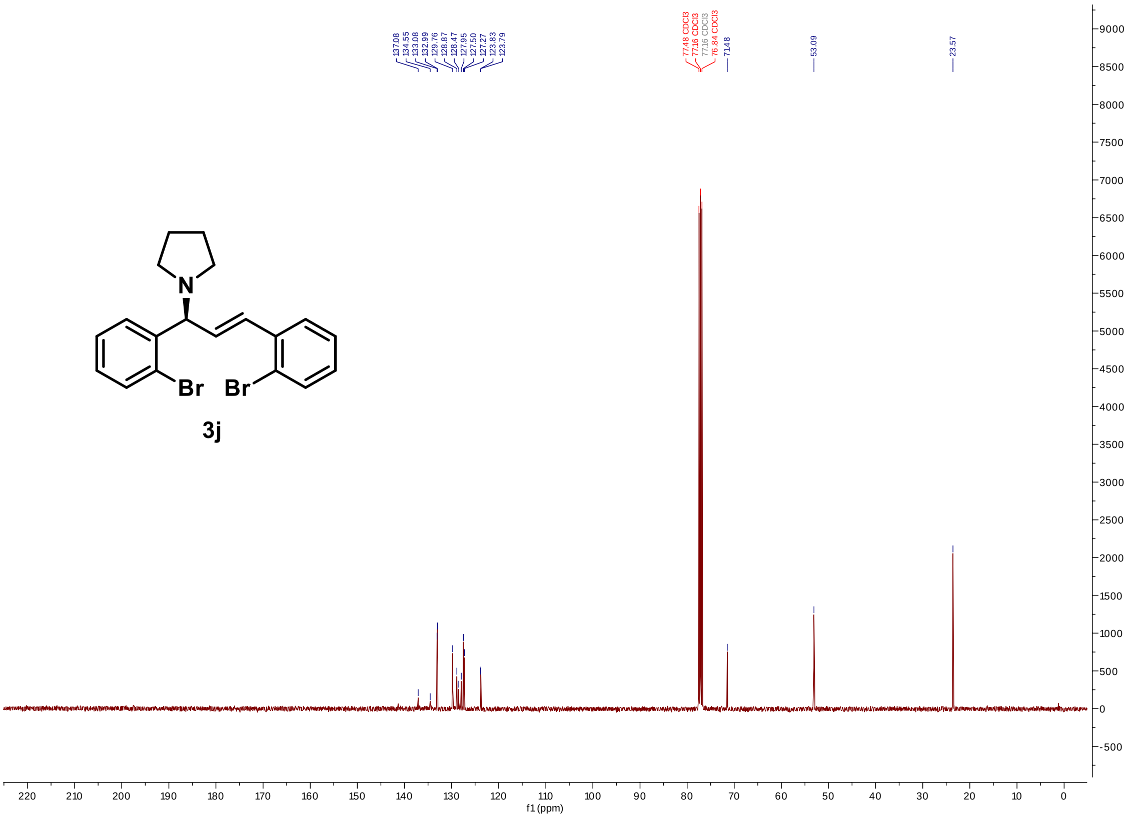

Figure S63. ^{13}C-NMR spectrum of **3j**

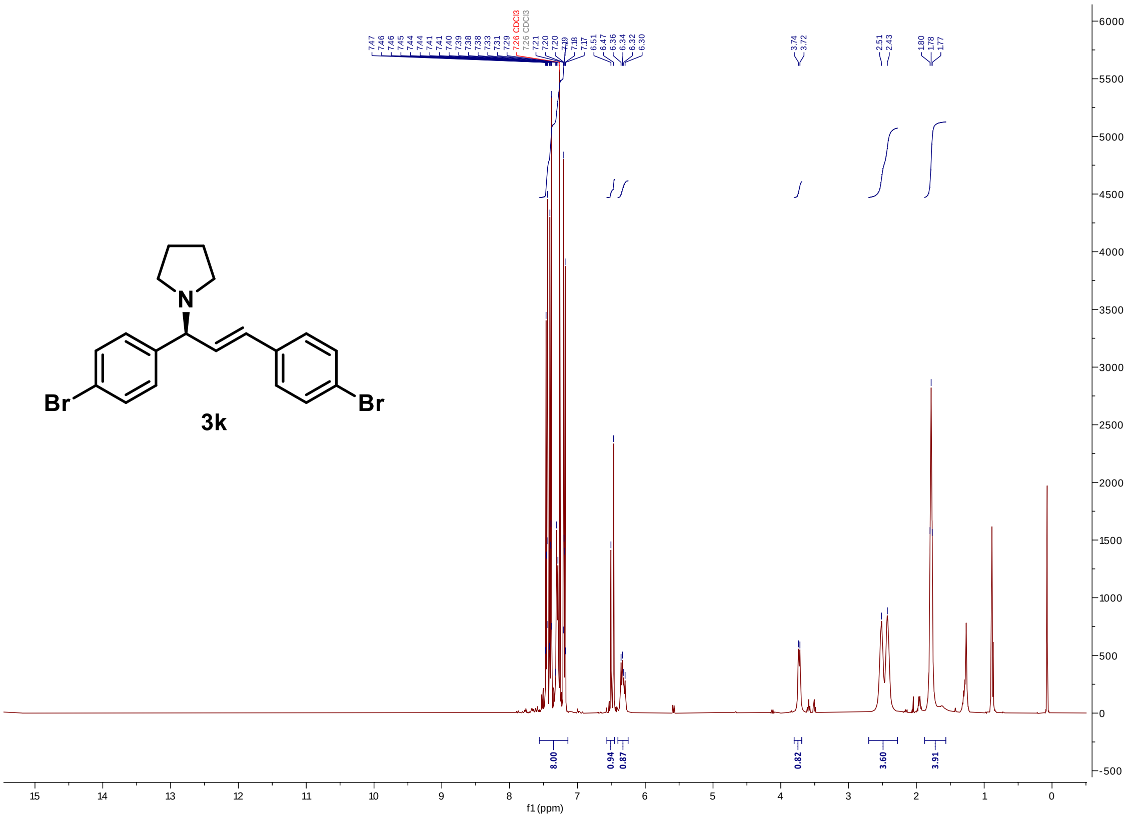

Figure S64. ^{1}H-NMR spectrum of **3k**

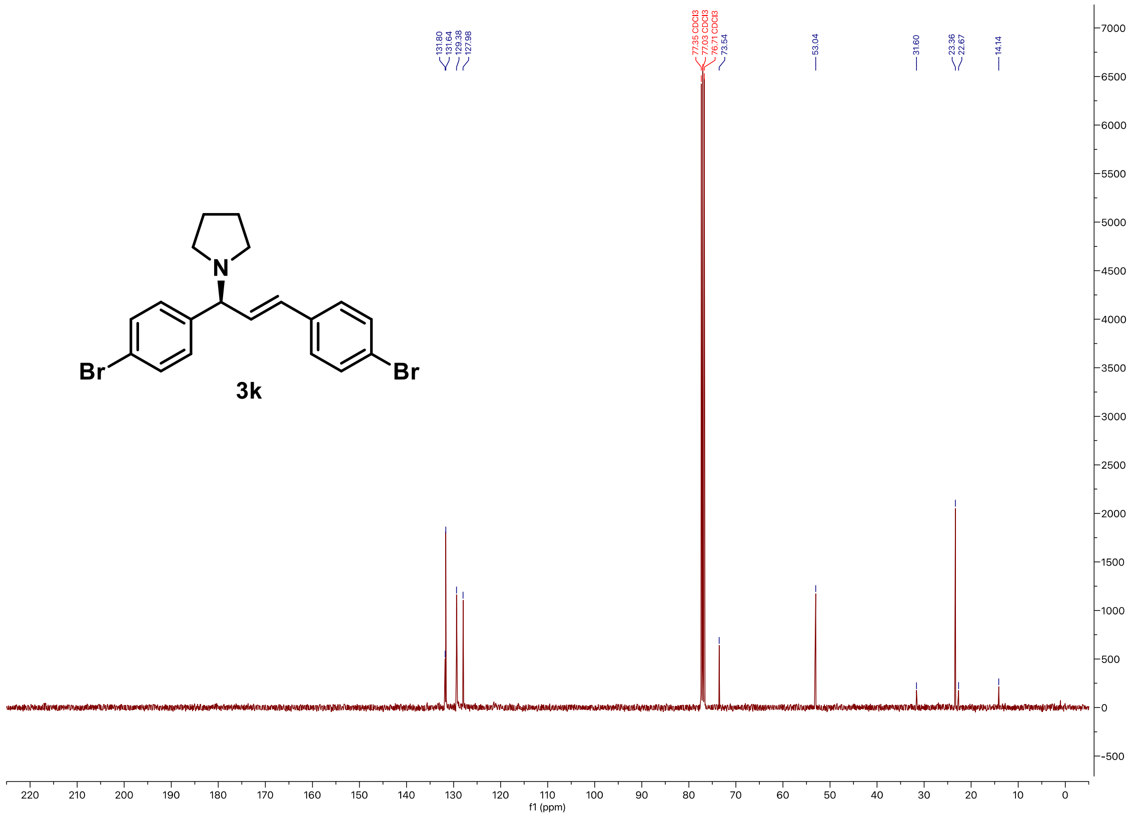

Figure S65. ^{13}C-NMR spectrum of **3k**

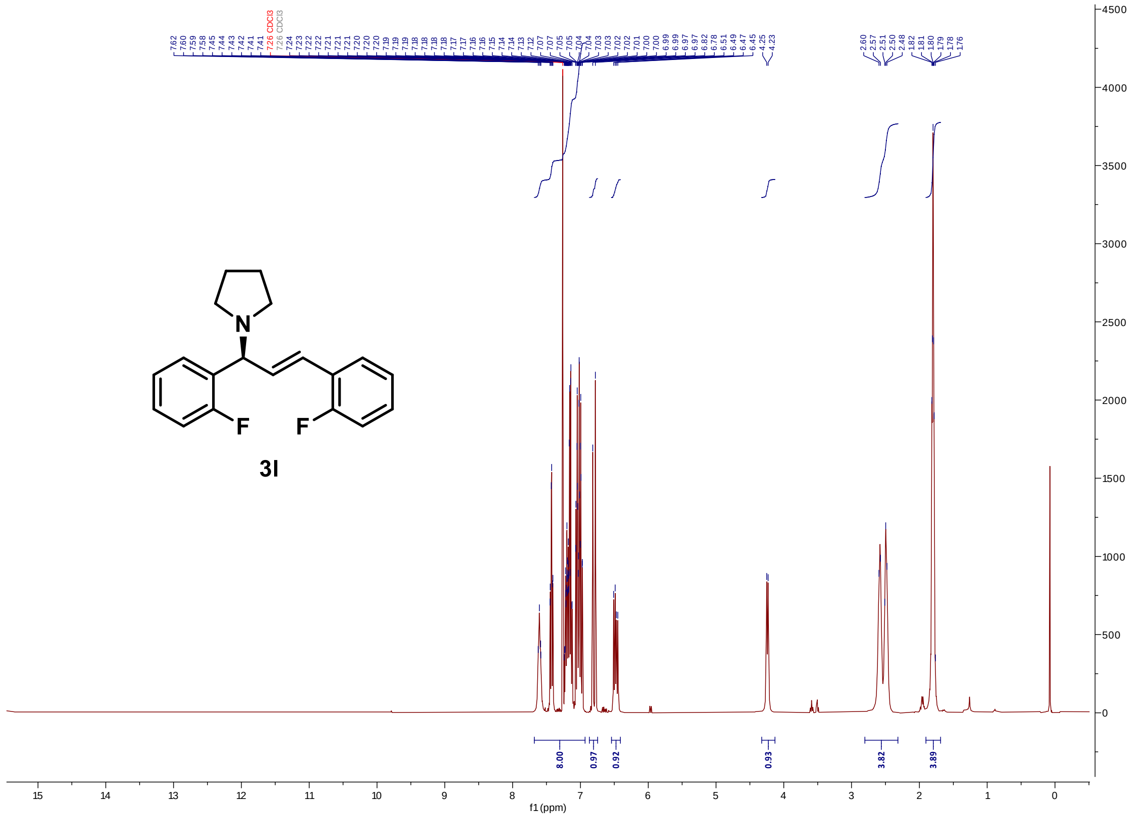

Figure S66. ^{1}H-NMR spectrum of **3l**

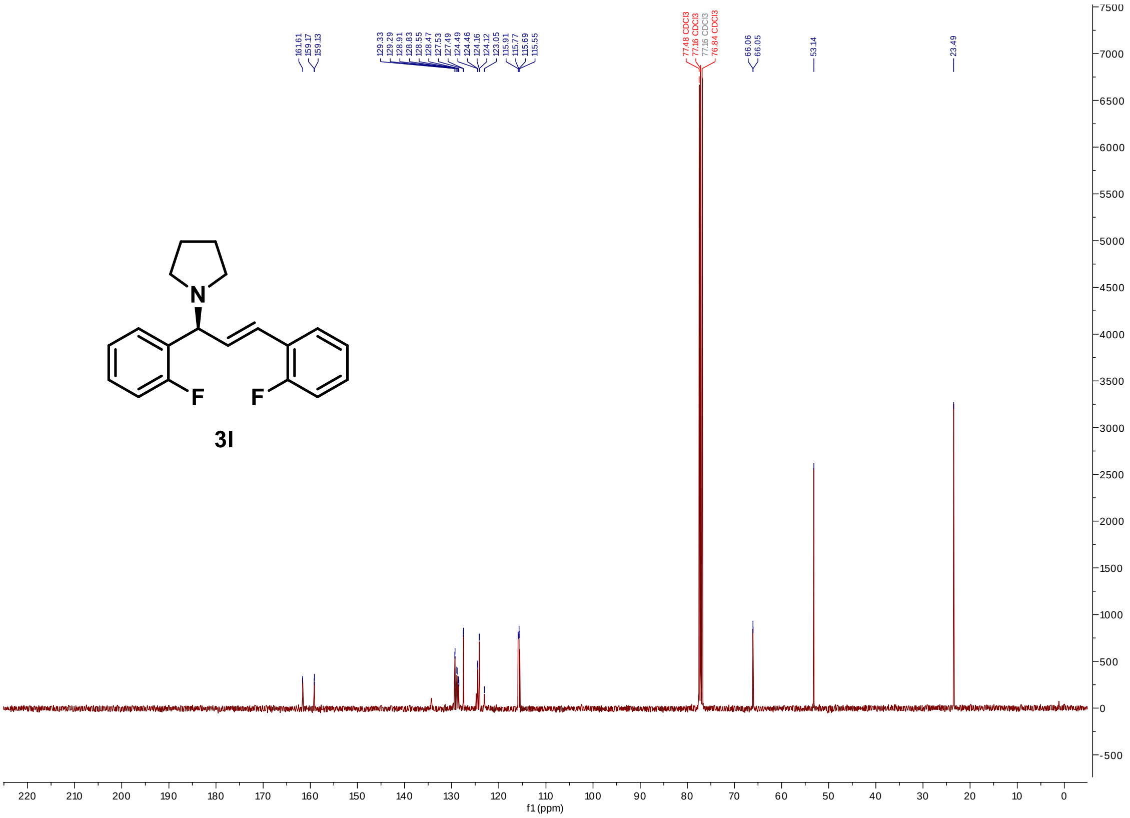

Figure S67. ^{13}C-NMR spectrum of **3l**

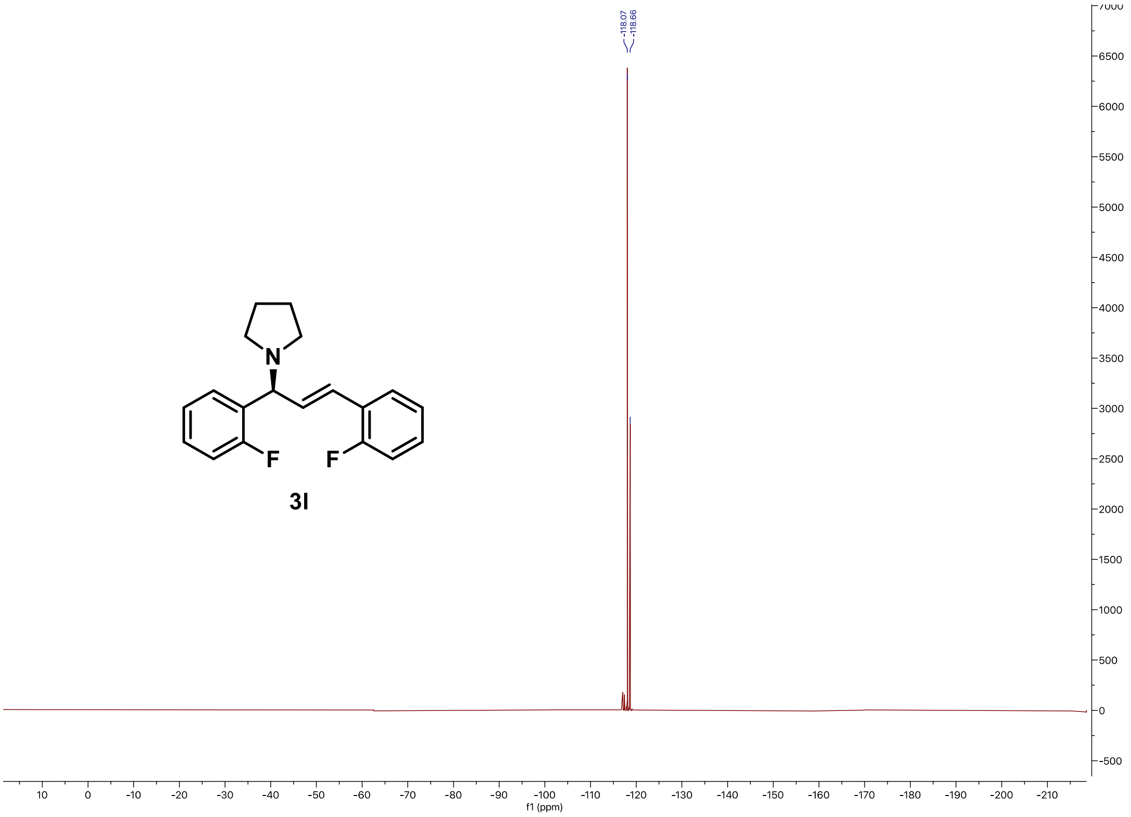

Figure S68. ^{19}F-NMR spectrum of **3l**

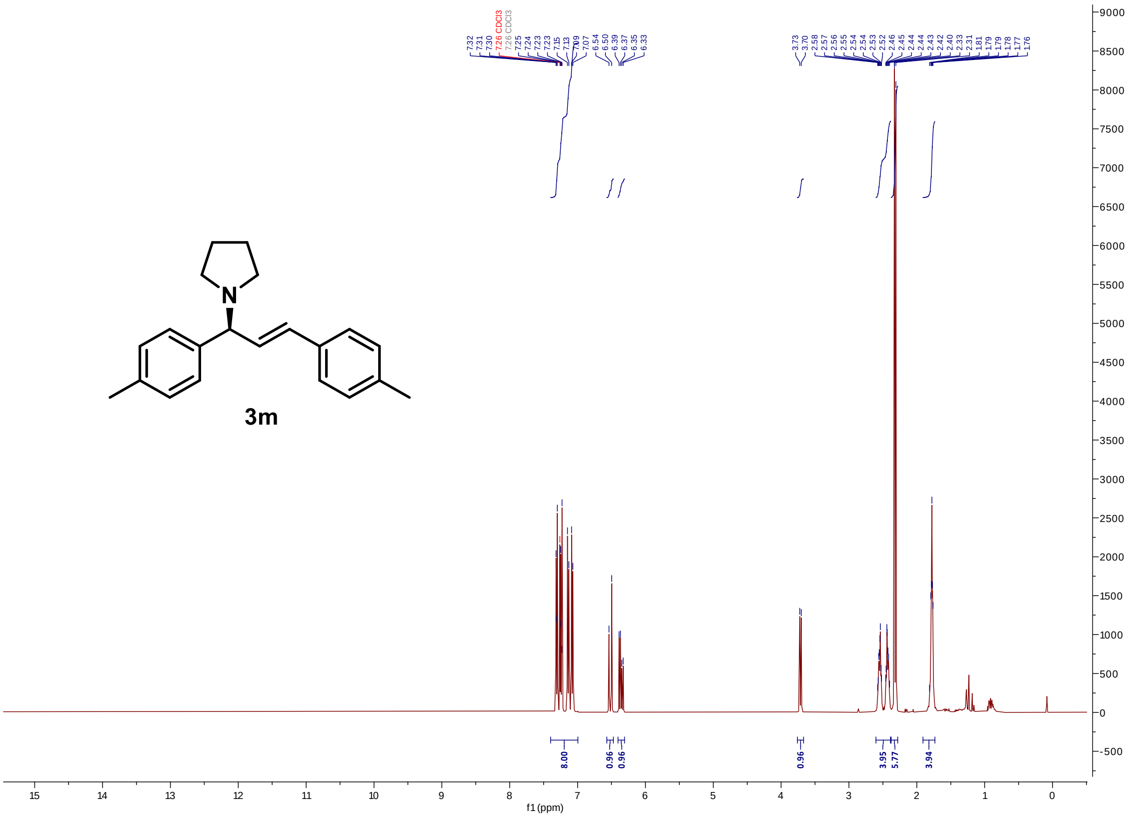

Figure S69. ^{1}H-NMR spectrum of **3m**

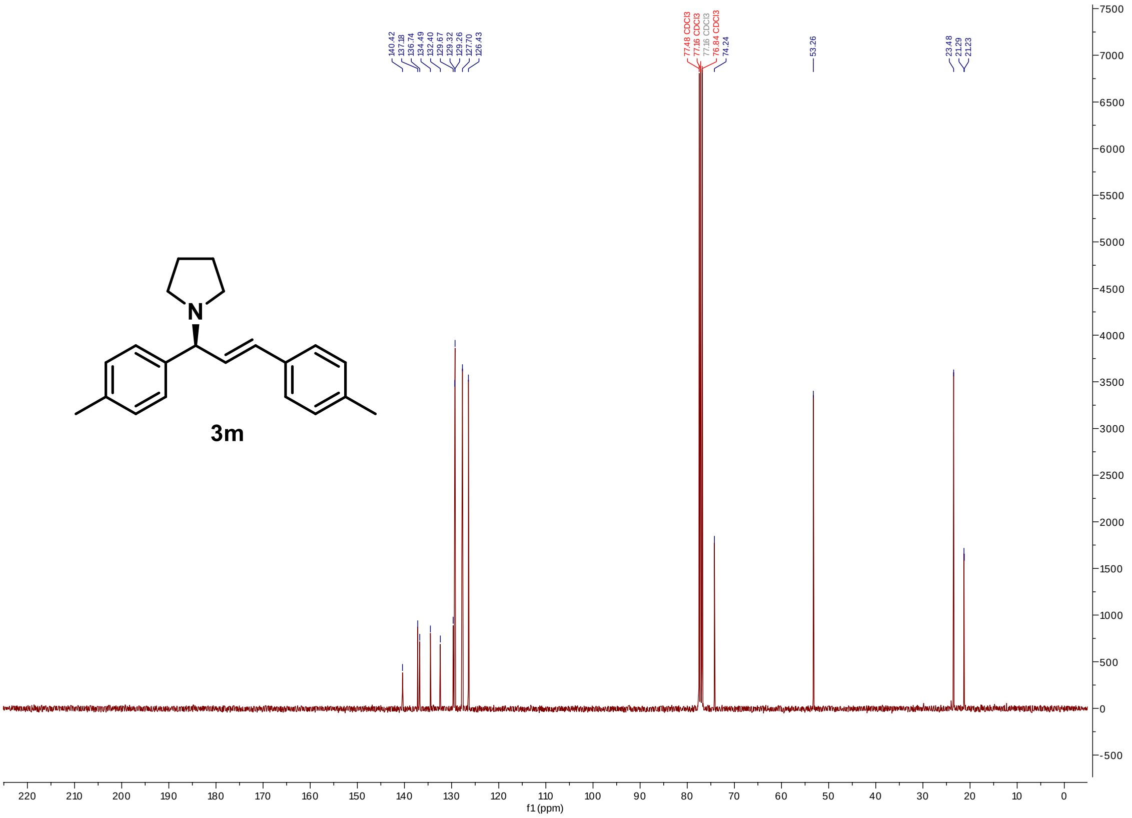

Figure S70. ^{13}C-NMR spectrum of **3m**

13. Chiral HPLC traces

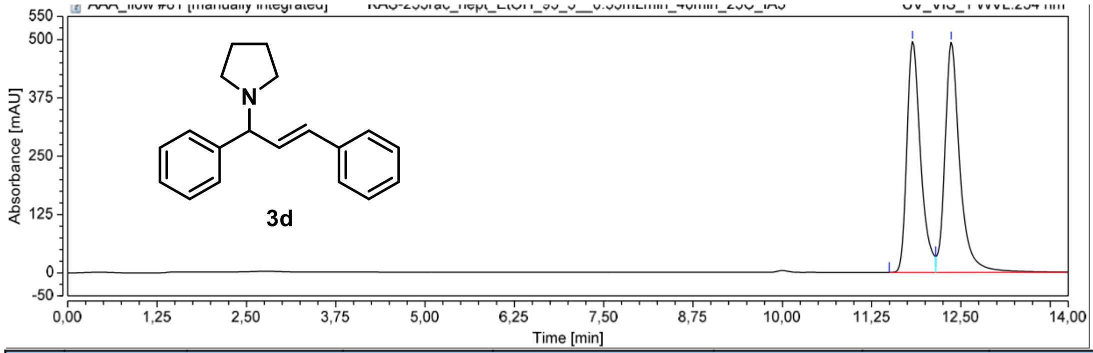

Integration Results							
No.	Peak Name	Retention Time<br>min	Area<br>mAU*min	Height<br>mAU	Relative Area<br>%	Relative Height<br>%	Amount<br>n.a.
1		11,823	109,371	495,556	47,63	50,09	n.a.
2		12,363	120,237	493,708	52,37	49,91	n.a.
**Total:**			**229,608**	**989,264**	**100,00**	**100,00**	

Figure S71. Chiral HPLC chromatogram of **3d** (racemic)

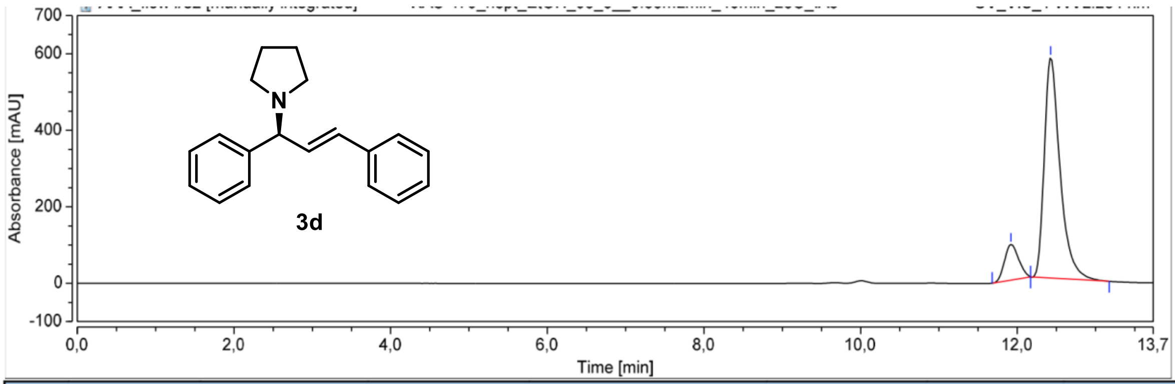

Integration Results							
No.	Peak Name	Retention Time<br>min	Area<br>mAU*min	Height<br>mAU	Relative Area<br>%	Relative Height<br>%	Amount<br>n.a.
1		11,920	19,557	93,447	12,68	13,94	n.a.
2		12,427	134,709	576,750	87,32	86,06	n.a.
**Total:**			**154,267**	**670,197**	**100,00**	**100,00**	

Figure S72. Chiral HPLC chromatogram of **3d** (enantioenriched)

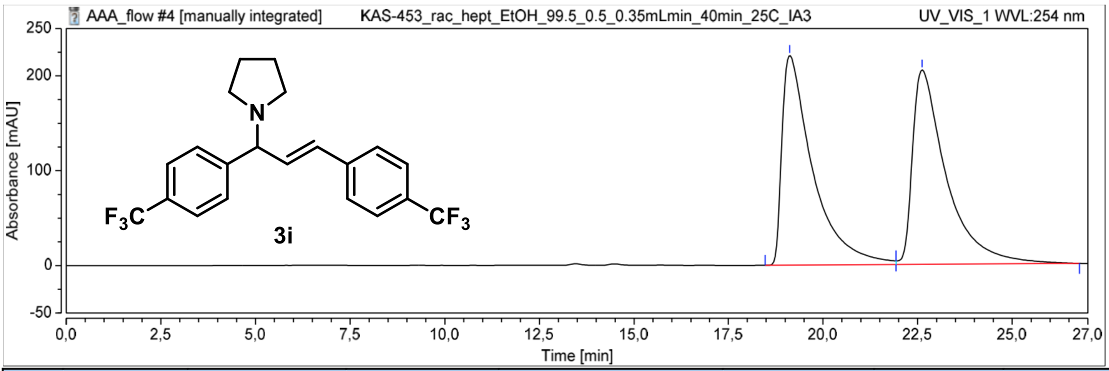

Integration Results							
No.	Peak Name	Retention Time min	Area mAU*min	Height mAU	Relative Area %	Relative Height %	Amount n.a.
1		19,123	208,043	221,024	49,48	51,89	n.a.
2		22,620	212,455	204,925	50,52	48,11	n.a.
**Total:**			**420,498**	**425,949**	**100,00**	**100,00**	

Figure S73. Chiral HPLC chromatogram of **3i** (racemic)

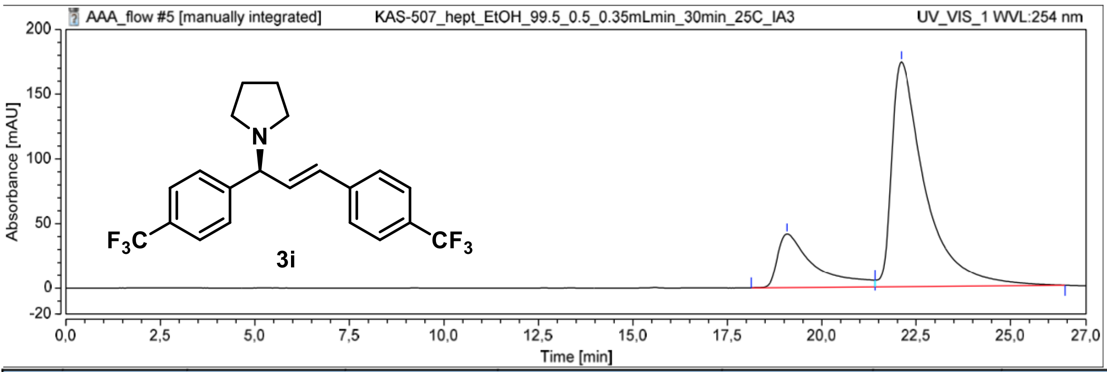

Integration Results							
No.	Peak Name	Retention Time min	Area mAU*min	Height mAU	Relative Area %	Relative Height %	Amount n.a.
1		19,080	47,088	41,698	20,77	19,34	n.a.
2		22,107	179,591	173,890	79,23	80,66	n.a.
**Total:**			**226,679**	**215,587**	**100,00**	**100,00**	

Figure S74. Chiral HPLC chromatogram of **3i** (enantioenriched)

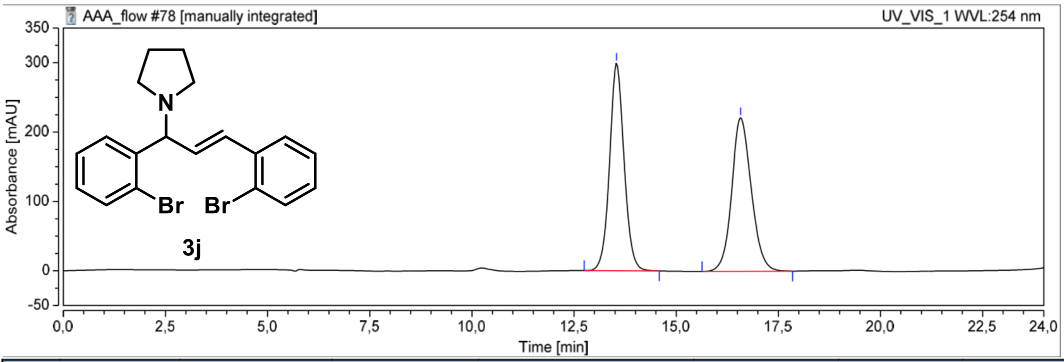

Integration Results							
No.	Peak Name	Retention Time min	Area mAU*min	Height mAU	Relative Area %	Relative Height %	Amount n.a.
1		13,537	123,972	299,347	50,20	57,47	n.a.
2		16,577	122,983	221,559	49,80	42,53	n.a.
**Total:**			**246,956**	**520,906**	**100,00**	**100,00**	

Figure S75. Chiral HPLC chromatogram of **3j** (racemic)

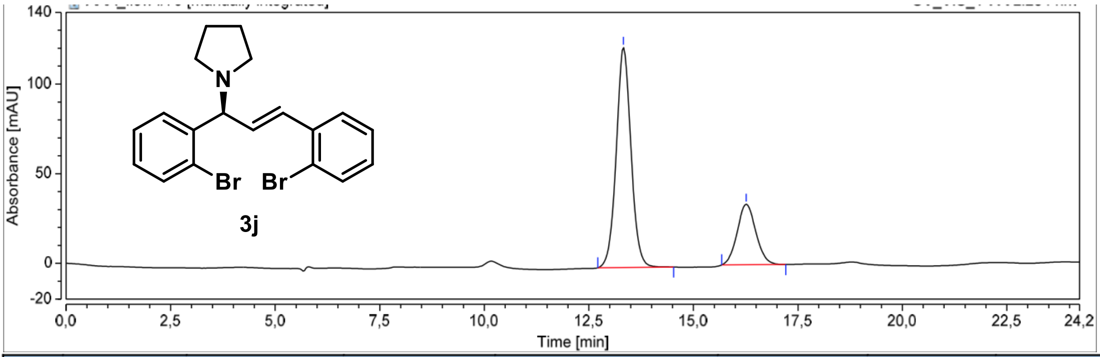

Integration Results							
No.	Peak Name	Retention Time min	Area mAU*min	Height mAU	Relative Area %	Relative Height %	Amount n.a.
1		13,327	50,315	122,897	74,27	78,45	n.a.
2		16,260	17,428	33,754	25,73	21,55	n.a.
**Total:**			**67,743**	**156,650**	**100,00**	**100,00**	

Figure S76. Chiral HPLC chromatogram of **3j** (enantioenriched)

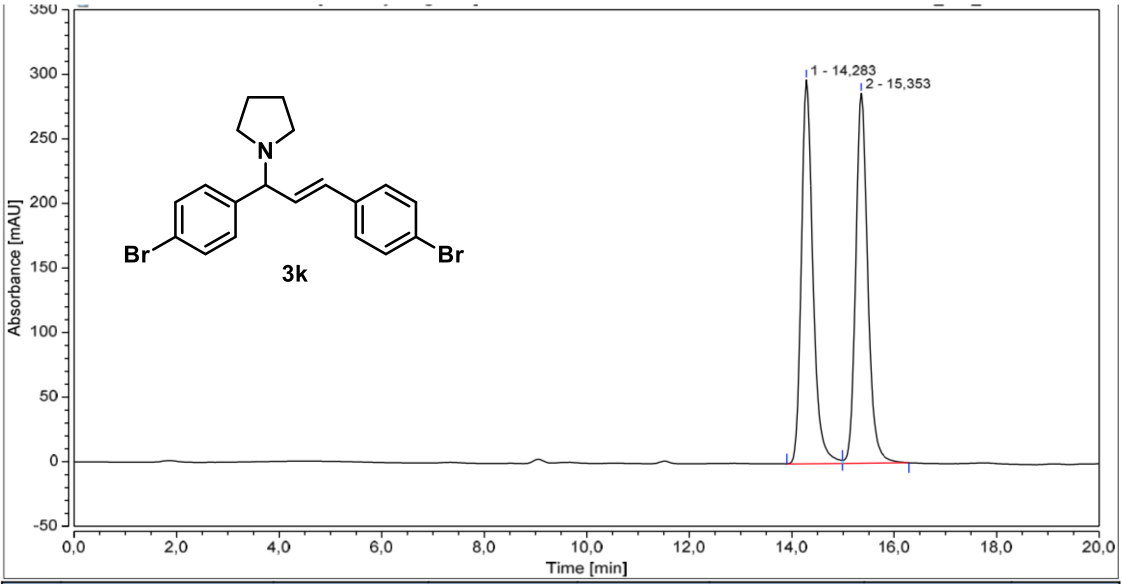

Integration Results							
No.	Peak Name	Retention Time<br>min	Area<br>mAU*min	Height<br>mAU	Relative Area<br>%	Relative Height<br>%	Amount
n.a.<br>1	1	n.a.<br>14,283	n.a.<br>77,738	n.a.<br>297,050	n.a.<br>49,92	n.a.<br>50,91	n.a.<br>n.a.
2		15,353	77,973	286,422	50,08	49,09	n.a.
**Total:**			**155,711**	**583,472**	**100,00**	**100,00**	

Figure S77. HPLC chromatogram of **3k** (racemic)

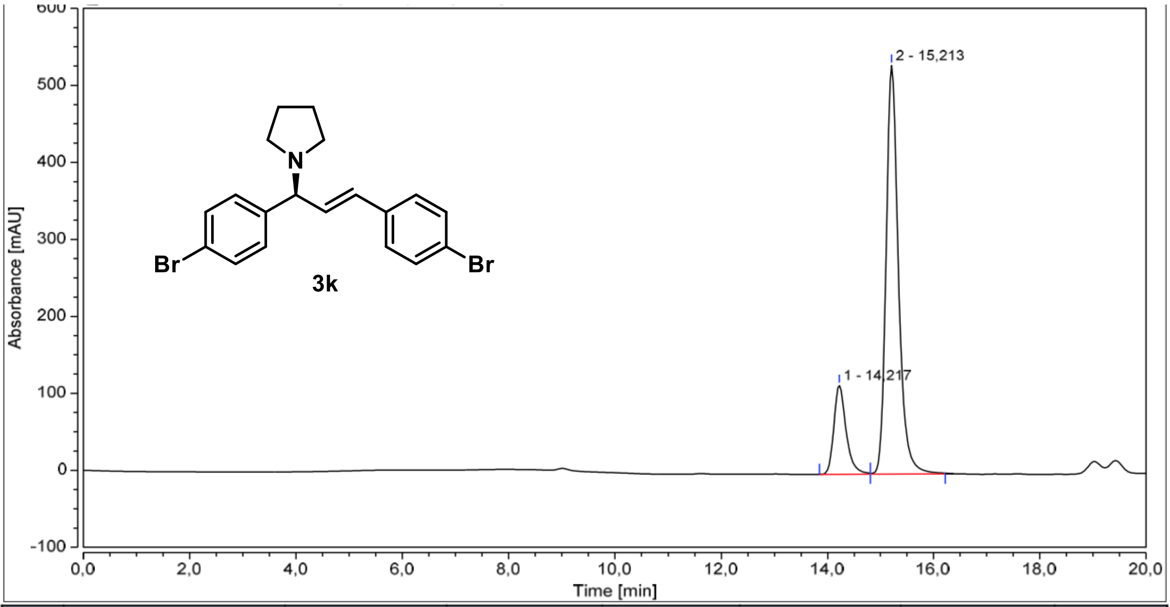

Integration Results							
No.	Peak Name	Retention Time<br>min	Area<br>mAU*min	Height<br>mAU	Relative Area<br>%	Relative Height<br>%	Amount
n.a.<br>1	1	n.a.<br>14,217	n.a.<br>30,896	n.a.<br>115,523	n.a.<br>17,72	n.a.<br>17,87	n.a.<br>n.a.
2		15,213	143,430	530,817	82,28	82,13	n.a.
**Total:**			**174,326**	**646,340**	**100,00**	**100,00**	

Figure S78. HPLC chromatogram of **3k** (enantioenriched)

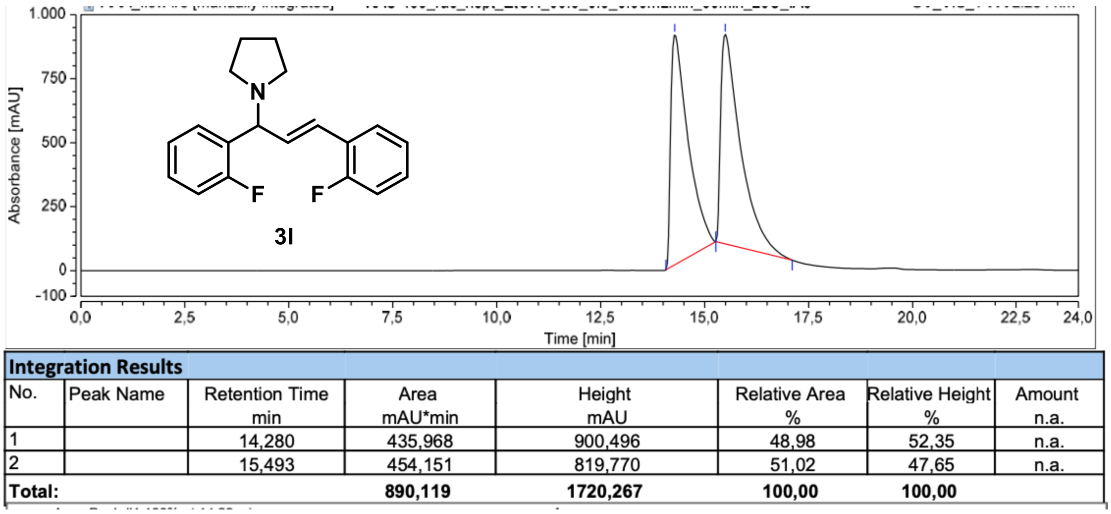

Integration Results

No.	Peak Name	Retention Time min	Area mAU*min	Height mAU	Relative Area %	Relative Height %	Amount n.a.
1		14,280	435,968	900,496	48,98	52,35	n.a.
2		15,493	454,151	819,770	51,02	47,65	n.a.
**Total:**			**890,119**	**1720,267**	**100,00**	**100,00**	

Figure S79. Chiral HPLC chromatogram of **3l** (racemic)

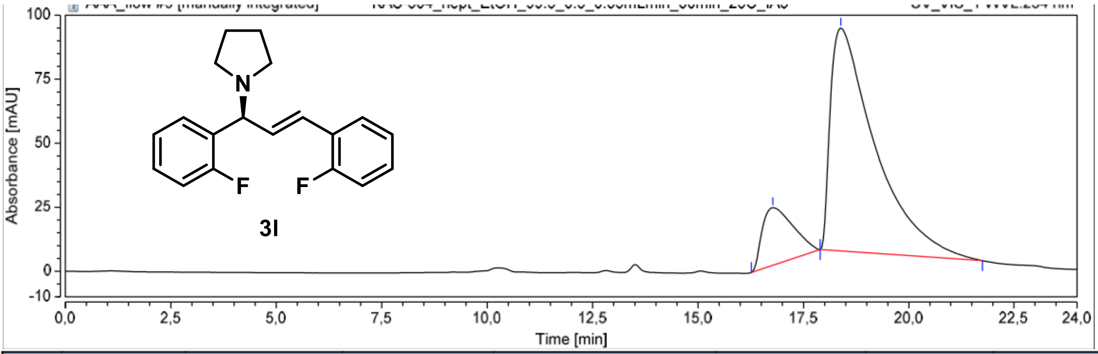

Integration Results

No.	Peak Name	Retention Time min	Area mAU*min	Height mAU	Relative Area %	Relative Height %	Amount n.a.
1		16,773	18,952	22,521	15,00	20,57	n.a.
2		18,383	107,393	86,977	85,00	79,43	n.a.
**Total:**			**126,345**	**109,498**	**100,00**	**100,00**	

Figure S80. Chiral HPLC chromatogram of **3l** (enantioenriched)

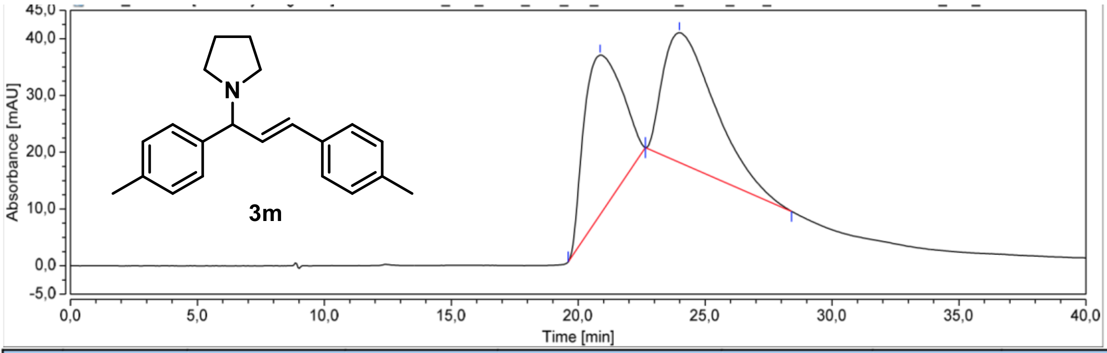

Integration Results							
No.	Peak Name	Retention Time min	Area mAU*min	Height mAU	Relative Area %	Relative Height %	Amount n.a.
1		20,863	48,322	28,055	46,48	55,11	n.a.
2		23,990	55,634	22,855	53,52	44,89	n.a.
**Total:**			**103,956**	**50,910**	**100,00**	**100,00**	

Figure S81. HPLC chromatogram of **3m** (racemic)

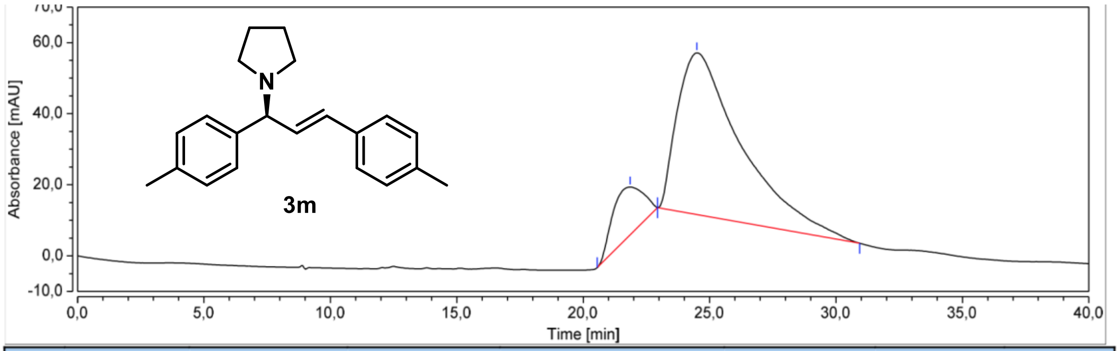

Integration Results							
No.	Peak Name	Retention Time min	Area mAU*min	Height mAU	Relative Area %	Relative Height %	Amount n.a.
1		21,857	19,977	13,495	13,03	22,87	n.a.
2		24,497	133,336	45,519	86,97	77,13	n.a.
**Total:**			**153,314**	**59,015**	**100,00**	**100,00**	

Figure S82. HPLC chromatogram of **3m** (enantioenriched)

14. References

[1] M. Nascimento de Oliveira, S. Arseniyadis, J. Cossy, *Chem. Eur. J.* **2018**, *24*, 4810-4814.

[2] A.-M. Carroll, M. McCarthy, P. M. Lacey, C. P. Saunders, D. J. Connolly, A. Farrell, B. V. Rokade, R. Goddard, P. Fristrup, P.-O. Norrby, P. J. Guiry, *Tetrahedron* **2020**, *76*, 130780.

[3] N. Boufroua, E. Dunach, F. Fontaine-Vive, S. Achouche-Bouzroura, S. Poulain-Martini, *New J. Chem.* **2020**, *44*, 6042-6052.

[4] H. Jin, X. Jiang, H. Yoo, T. Wang, C. G. Sung, U. Choi, C. R. Lee, H. Yu, S. Koo, *ChemistrySelect* **2020**, *5*, 12421-12424.

[5] P.-S. Gao, F. Ye, X.-Y. Dong, Y. Chen, Z.-W. Gao, W.-Q. Zhang, L.-W. Xu, *RSC Adv.* **2015**, *5*, 33818-33822.

[6] J. Jing, X. Huo, J. Shen, J. Fu, Q. Meng, W. Zhang, *Chem. Commun.* **2017**, *53*, 5151-5154.

[7] K. N. Gavrilov, S. V. Zheglov, M. N. Gavrilova, I. M. Novikov, M. G. Maksimova, N. N. Groshkin, E. A. Rastorguev, V. A. Davankov, *Tetrahedron* **2012**, *68*, 1581-1589.

[8] T. H. West, D. S. B. Daniels, A. M. Z. Slawin, A. D. Smith, *J. Am. Chem. Soc.* **2014**, *136*, 4476-4479.

[9] D. Krishnan, M. Wu, M. Chiang, Y. Li, P.-H. Leung, S. A. Pullarkat, *Organometallics* **2013**, *32*, 2389-2397.

[10] T. Ohshima, Y. Miyamoto, J. Ipposhi, Y. Nakahara, M. Utsunomiya, K. Mashima, *J. Am. Chem. Soc.* **2009**, *131*, 14317-14328.

[11] D. Banerjee, R. V. Jagadeesh, K. Junge, H. Junge, M. Beller, *ChemSusChem* **2012**, *5*, 2039-2044.

[12] B. M. Trost, D. L. Van Vranken, C. Bingel, *J. Am. Chem. Soc.* **1992**, *114*, 9327-9343.

[13] Y. K. Kim, S. J. Lee, K. H. Ahn, *J. Org. Chem.* **2000**, *65*, 7807-7813.

[14] M. Marinova, M. Torres-Werlé, G. Taupier, A. Maisse-François, T. Achard, A. Boeglin, K. D. H. Dorkenoo, S. Bellemin-Laponnaz, *ACS Omega* **2019**, *4*, 2676-2683.
